# Substituted l-tryptophan-l-phenyllactic acid conjugates produced by an endophytic fungus *Aspergillus aculeatus* using an OSMAC approach†

**DOI:** 10.1039/c8ra00200b

**Published:** 2018-02-19

**Authors:** Hao Wang, Peter M. Eze, Simon-Patrick Höfert, Christoph Janiak, Rudolf Hartmann, Festus B. C. Okoye, Charles O. Esimone, Raha S. Orfali, Haofu Dai, Zhen Liu, Peter Proksch

**Affiliations:** Institute of Pharmaceutical Biology and Biotechnology, Heinrich-Heine-University Düsseldorf Universitätsstrasse 1 40225 Düsseldorf Germany zhenfeizi0@sina.com proksch@uni-duesseldorf.de; Department of Pharmaceutical Microbiology and Biotechnology, Faculty of Pharmaceutical Sciences, Nnamdi Azikiwe University Awka Nigeria; Institute of Inorganic and Structural Chemistry, Heinrich-Heine-University Düsseldorf Universitätsstrasse 1 40225 Düsseldorf Germany; Institute of Complex Systems: Structural Biochemistry, Forschungszentrum Juelich Wilhelm-Johnen-Straße, 52428 Juelich Germany; Department of Pharmaceutical and Medicinal Chemistry, Faculty of Pharmaceutical Sciences, Nnamdi Azikiwe University Awka Nigeria; Department of Pharmacognosy, Faculty of Pharmacy, King Saud University Riyadh Saudi Arabia; Key Laboratory of Biology and Genetic Resources of Tropical Crops, Ministry of Agriculture, Institute of Tropical Bioscience and Biotechnology, Chinese Academy of Tropical Agricultural Sciences Haikou 571101 China

## Abstract

The endophytic fungus *Aspergillus aculeatus* isolated from leaves of the papaya plant *Carica papaya* was fermented on solid rice medium, yielding a new l-tryptophan-l-phenyllactic acid conjugate (1) and thirteen known compounds (11, 14–25). In addition, an OSMAC approach was employed by adding eight different sodium or ammonium salts to the rice medium. Addition of 3.5% NaNO_3_ caused a significant change of the metabolite pattern of the fungus as indicated by HPLC analysis. Subsequent isolation yielded several new substituted l-tryptophan-l-phenyllactic acid conjugates (1–10) in addition to three known compounds (11–13), among which compounds 2–10, 12–13 were not detected in the rice control culture. All structures were unambiguously elucidated by one and two dimensional NMR spectroscopy and by mass spectrometry. The absolute configuration of the new compounds was determined by Marfey's reaction and X-ray single crystal diffraction. Compounds 19–22 showed cytotoxicity against the L5178Y mouse lymphoma cell line with IC_50_ values of 3.4, 1.4, 7.3 and 23.7 μM, respectively.

## Abstract 2

Indole-derived alkaloids from *Aspergillus aculeatus* using an OSMAC approach.

## Graphical abstract

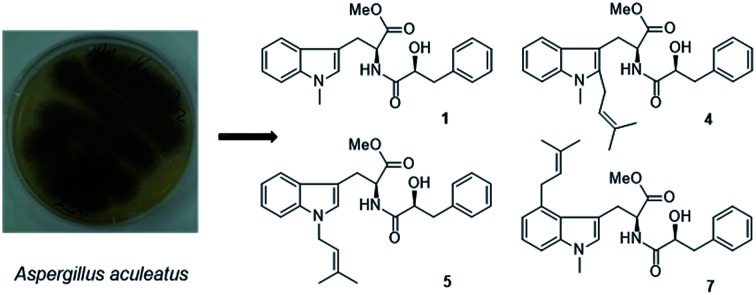

## Introduction

Endophytic fungi thrive widely in different healthy tissues of living plants, and have significant influence on the growth of their hosts.^1,2^ Endophytes also comprise a large reservoir of structurally diverse secondary metabolites including alkaloids, steroids, terpenoids, xanthones, peptides and quinones, which exhibit a variety of biological activities including anticancer, antibacterial, antifungal, anti-inflammatory and antidepressant activity.^2,3^ For example, 14-membered macrolides isolated from the endophytic fungus *Pestalotiopsis microspora* showed significant cytotoxicity against the murine lymphoma cell line L5178Y while fusaric acid derivatives produced by the fungal endophyte *Fusarium oxysporum* showed significant phytotoxicity to leaves of barley.^4,5^ However, the high rediscovery rate of known compounds from fungi is a severe obstacle for the search for new drug leads.^6^ One of the approaches to increase the diversity of metabolites from fungi involves the application of the OSMAC (One Strain Many Compounds) method which is based on systematic variations of the cultivation parameters (media type and composition, pH value, temperature *etc.*).^7–9^ For instance, the fermentation of the fungus *Gymnascella dankaliensis* on solid rice medium following addition of 3.5% NaBr led to the isolation of ten brominated tyrosine-derived alkaloids, which showed cytotoxicity against the L5178Y mouse lymphoma cell line.^10,11^ In addition, cultivation of *Fusarium tricinctum* on fruit and vegetable juice-supplemented solid rice media that led to the production of the new fusarielins K and L.^12^

The fungus *Aspergillus aculeatus* has been reported to produce several bioactive secondary metabolites, such as secalonic acids D and F, aculeatusquinone B and D, aculeacins A–G and aspergillusol A.^13–17^ In this study, *A. aculeatus* was isolated from leaves of *Carica papaya* collected in Awka in Nigeria. The fungus was fermented on solid rice medium, yielding a new l-tryptophan-l-phenyllactic acid conjugate (1) together with thirteen known compounds including *N*-[(2*S*)-2-hydroxy-1-oxo-3-phenylpropyl]-l-tryptophan methyl ester (11),^18^ okaramine A (14),^19^ oxaline (15),^20^ emindole SB (16),^21^ 16-keto-aspergillimide (17),^22^ JBIR 75 (18),^23^ secalonic acids D and F (19 and 20),^13^ asperdichrome (21),^24^ RF 3192C (22),^25^ pannorin (23),^26^ altechromone A (24),^27^ and variecolactone (25).^28^ Due to the pronounced chemical diversity of this fungus we decided to subject *A. aculeatus* to an OSMAC approach by adding either 3.5% NaCl, 3.5% NaBr, 3.5% NaI, 1% NaF, 3.5% NaNO_3_, 3.5% NH_4_Cl, 3.5% (NH_4_)_2_SO_4_ or 3.5% NH_4_OAc to the rice medium. The selection of these salts for the OSMAC study was based on previous experiments with other fungi that had indicated the usefulness of these chemical stimuli for the accumulation of cryptic metabolites.^10,11,29^ The fungus did not grow on rice medium containing 1% NaF or 3.5% NH_4_OAc. Addition of 3.5% NaNO_3_, however, caused a significant change of the metabolite pattern as indicated by HPLC analysis. Addition of the remaining salts had no influence. Subsequent workup of the extract resulting from addition of 3.5% NaNO_3_ yielded several new substituted l-tryptophan-l-phenyllactic acid conjugates (1–10) in addition to three known analogues *N*-[(2*S*)-2-hydroxy-1-oxo-3-phenylpropyl]-l-tryptophan methyl ester (11), *N*-[(2*S*)-2-hydroxy-1-oxo-3-phenylpropyl]-l-tryptophan (12) and acu-dioxomorpholine (13).^18^ Compounds 2–10, 12–13 were not detected in the rice control culture. On the other hand, from all compounds isolated from the rice control culture, only oxaline (15), JBIR 75 (18), secalonic acid F (20) and RF 3192C (22) could be detected in the fungal culture after addition of 3.5% NaNO_3_. In this study we report the structure elucidation and the biological activities of the isolated compounds.

## Results and discussion

**Fig. 1:**
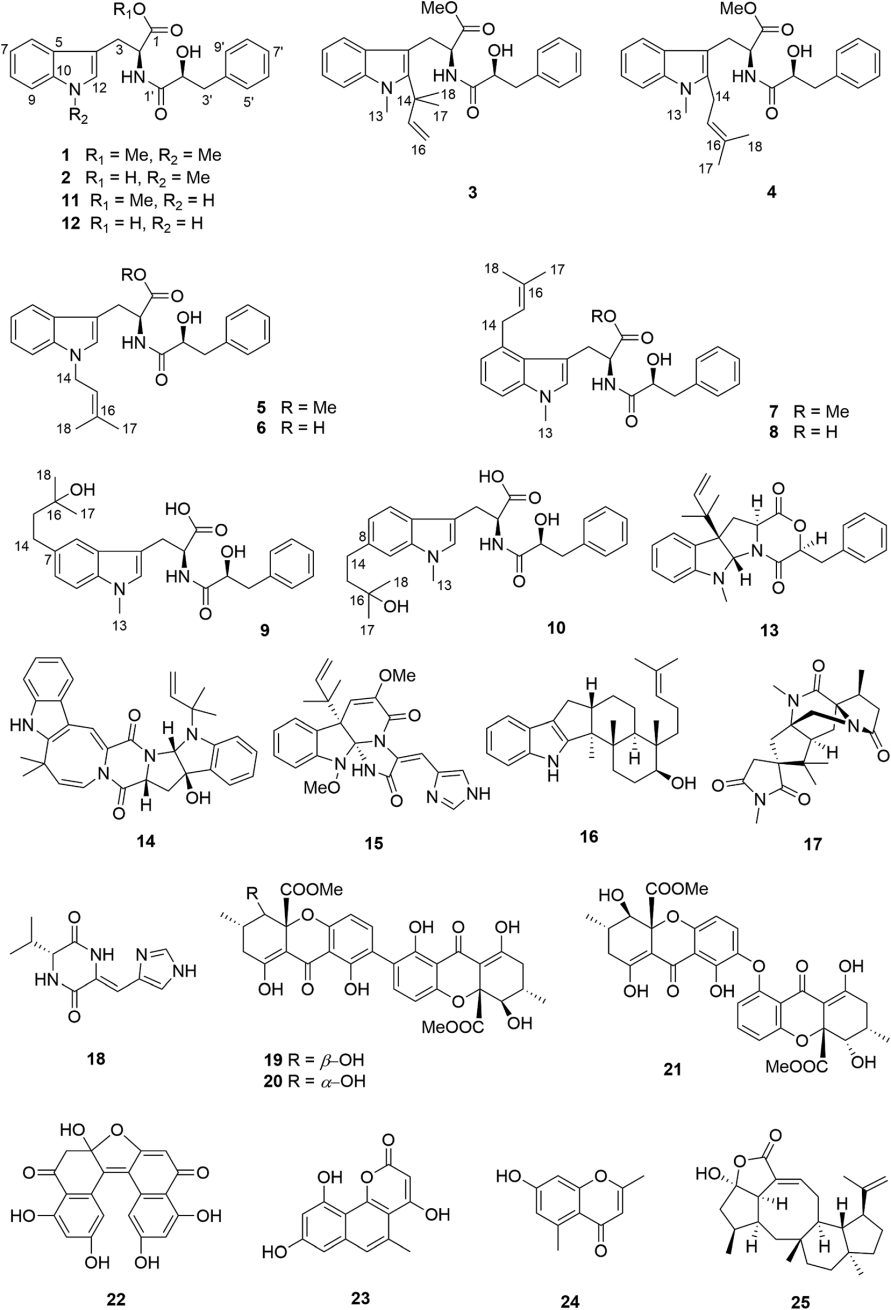
Structures of compounds 1–25 isolated from *A. aculeatus*.

**Fig. 2:**
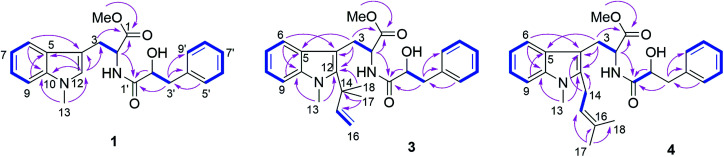
COSY and key HMBC correlations of compounds 1, 3 and 4.

**Fig. 3:**
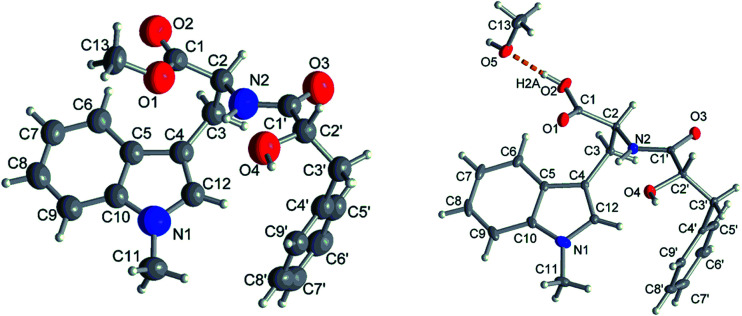
Molecular structures of 1 and 2 from single-crystal X-ray diffractometry (50% thermal ellipsoids, H-atoms with arbitrary radii). The structure drawing of 2 shows also the hydrogen bond (orange dashed line) to the methanol crystal solvent molecule.

Compound 1 had the molecular formula C_22_H_24_O_4_N_2_ as established by HRESIMS exhibiting 12 degrees of unsaturation. The ^13^C NMR spectrum of 1 (Table 1) included 22 signals, corresponding to two methyls, two methylenes, two alphatic methines and ten aromatic methines as well as six quaternary carbons. The ^1^H NMR spectrum of 1 (Table 2) showed the presence of a monosubstituted benzene ring and an indole moiety as indicated by signals between *δ*_H_ 6 and 8, two –CH_2_–CH– units and two methyl groups (*δ*_H_ 3.70 and 3.62). These data were similar to those of the co-isolated known compound *N*-[(2*S*)-2-hydroxy-1-oxo-3-phenylpropyl]-l-tryptophan methyl ester (11).^18^ However, the detection of an additional methyl substituent at *δ*_C_ 32.7 and *δ*_H_ 3.70 and the HMBC correlation from the protons of this methyl group to C-10 (*δ*_C_ 138.5) and C-12 (*δ*_C_ 129.0) indicated its attachment to N-11. Detailed analysis of the 2D NMR spectra of 1 (Fig. 2) revealed that its remaining substructure was identical to that of 11. The absolute configuration of 1 was determined by X-ray single crystal analysis as 2*S*, 2′*S* (Fig. 3), being identical to that of 11. Thus, the structure of 1, for which the trial name aculeatine A is proposed, was elucidated as shown in Fig. 1.

**Table 1:** ^13^C NMR data of compounds 1–5 (CD_3_OD, 150 MHz)

No.	1	2	3	4	5
1	173.4, C	174.8, C	173.9, C	173.8, C	173.5, C
2	53.7, CH	53.7, CH	55.3, CH	54.3, CH	53.6, CH
3	28.4, CH_2_	28.4, CH_2_	29.2, CH_2_	28.0, CH_2_	28.5, CH_2_
4	109.2, C	109.5, C	107.5, C	106.0, C	109.4, C
5	129.1, C	129.4, C	130.3, C	129.1, C	129.4, C
6	119.6, CH	119.8, CH	119.2, CH	119.0, CH	119.6, CH
7	119.9, CH	119.9, CH	120.1, CH	120.0, CH	120.0, CH
8	122.6, CH	122.5, CH	122.7, CH	122.0, CH	122.5, CH
9	110.2, CH	110.1, CH	109.7, CH	109.7, CH	110.6, CH
10	138.5, C	138.5, C	139.0, C	138.4, C	137.8, C
12	129.0, CH	129.0, CH	142.2, C	138.8, C	127.6, CH
13	32.7, CH_3_	32.7, CH_3_	33.8, CH_3_	30.0, CH_3_	
14			41.9, C	24.8, CH_2_	44.8, CH_2_
15			149.1, CH	122.4, CH	121.5, CH
16			112.8, CH_2_	133.9, C	137.1, C
17			30.0, CH_3_	25.8, CH_3_	25.8, CH_3_
18			29.9, CH_3_	18.1, CH_3_	18.1, CH_3_
1′	175.8, C	175.8, C	176.0, C	176.1, C	175.9, C
2′	73.4, CH	73.6, CH	73.7, CH	73.7, CH	73.5, CH
3′	41.4, CH_2_	41.5, CH_2_	41.2, CH_2_	41.4, CH_2_	41.6, CH_2_
4′	138.8, C	139.0, C	139.1, C	138.9, C	138.9, C
5′, 9′	130.9, CH	130.9, CH	130.6, CH	130.6, CH	130.9, CH
6′, 8′	129.1, CH	129.1, CH	129.0, CH	129.0, CH	129.1, CH
7′	127.5, CH	127.5, CH	127.3, CH	127.4, CH	127.5, CH
OMe	52.8, CH_3_		52.6, CH_3_	52.8, CH_3_	52.8, CH_3_

**Table 2:** ^1^H NMR data of compounds 1–5 (CD_3_OD, 600 MHz)

No.	1	2	3	4	5
2	4.74, dd (6.2, 5.4)	4.71, dd (6.2, 5.2)	4.69, dd (8.5, 7.3)	4.65, dd (7.2, 6.6)	4.74, dd (6.1, 5.4)
3	3.21, dd (14.6, 6.2), 3.07, dd (14.6, 5.4)	3.24, dd (14.7, 6.2), 3.14, dd (14.7, 5.2)	3.45, dd (14.9, 7.3), 3.26, dd (14.9, 8.5)	3.15, dd (14.7, 6.6), 3.10, dd (14.7, 7.2)	3.22, dd (14.7, 6.1), 3.05, dd (14.7, 5.4)
6	7.41, d (7.9)	7.49, d (7.9)	7.50, d (7.8)	7.40, d (7.8)	7.40, d (7.9)
7	7.03, dd (7.9, 7.2)	7.02, dd (7.9, 7.2)	7.07, dd (7.8, 7.2)	7.02, dd (7.8, 7.1)	7.02, dd (7.9, 7.1)
8	7.15, dd (8.2, 7.2)	7.14, dd (8.2, 7.2)	7.14, dd (8.1, 7.2)	7.10, dd (8.2, 7.1)	7.13, dd (8.2, 7.1)
9	7.29, d (8.2)	7.29, d (8.2)	7.25, d (8.1)	7.26, d (8.2)	7.29, d (8.2)
12	6.61, s	6.66, s			6.71, s
13	3.70, s	3.70, s	3.72, s	3.60, s	
14				3.48, dd (16.6, 6.6), 3.44, dd (16.6, 6.6)	4.66, d (6.9)
15			6.23, dd (17.5, 10.6)	5.11, br t (6.6)	5.32, br t (6.9)
16			5.11, d (10.6), 4.98, d (17.5)		
17			1.66, s	1.74, s	1.74, s
18			1.66, s	1.82, s	1.83, s
2′	4.24, dd (7.1, 4.0)	4.22, dd (7.4, 3.8)	4.10, dd (8.1, 3.7)	4.17, dd (7.8, 3.8)	4.22, dd (7.3, 3.9)
3′	2.97, dd (13.9, 4.0), 2.78, dd (13.9, 7.1)	2.96, dd (13.9, 3.8), 2.73, dd (13.9, 7.4)	2.64, dd (13.9, 3.7), 2.24, dd (13.9, 8.1)	2.85, dd (13.9, 3.8), 2.56, dd (13.9, 7.8)	2.96, dd (13.9, 3.9), 2.76, dd (13.9, 7.3)
5′, 9′	7.20, d (7.0)	7.19, t (7.1)	7.05, d (7.1)	7.11, d (7.2)	7.20, d (7.2)
6′, 8′	7.24, t (7.0)	7.22, d (7.1)	7.15, t (7.1)	7.16, t (7.2)	7.23, t (7.2)
7′	7.17, t (7.0)	7.17, t (7.1)	7.12, t (7.1)	7.12, t (7.2)	7.17, t (7.2)
OMe	3.62, s		3.49, s	3.60, s	3.63, s

Compound 2 possessed the molecular formula C_21_H_22_O_4_N_2_ as determined by HRESIMS, which indicated the loss of a methyl group compared to 1. This was confirmed by the absence of the ^1^H and ^13^C signals of the methoxy group attached to C-1 in the NMR spectra of 2 (Tables 1 and 2). The structure of 2 was confirmed by detailed analysis of the 2D NMR spectra. Its absolute configuration was determined as 2*S*, 2′*S* by X-ray single crystal analysis (Fig. 3).

The molecular formula of compound 3 was determined as C_27_H_32_O_4_N_2_ by HRESIMS. The ^13^C NMR spectrum of 3 exhibited five additional carbons compared to 1. The ^1^H NMR spectrum of 3 was similar to that of compound 1 except for the observation of two singlet methyls at *δ*_H_ 1.66 (Me-17 and 18) and signals of a terminal double bond at *δ*_H_ 6.23 (H-15), 5.11 and 4.98 (H_2_-16) as well as the absence of the singlet aromatic proton at C-12. The COSY correlations between H-15 and H_2_-16 together with the HMBC correlations from Me-17 and Me-18 to C-12 (*δ*_C_ 142.2), C-14 (*δ*_C_ 41.9) and C-15 (*δ*_C_ 149.1) and from Me-13 (*δ*_H_ 3.72) to C-12 and C-10 (*δ*_C_ 139.0) indicated the attachment of a 1,1-dimethylprop-2-en-1-yl side chain at C-12. Detailed analysis of the 2D NMR spectra of 3 (Fig. 2) revealed that its remaining substructure was identical to that of 1.

Compound 4 had the same molecular formula as 3. The ^1^H and ^13^C NMR data were also similar to those of 3. The appearance of a broad triplet proton (*δ*_H_ 5.11, H-15) and one methylene group (*δ*_H_ 3.48 and 3.44, H_2_-14) together with two olefinic methyl groups (*δ*_H_ 1.74 and 1.82, Me-17 and 18) in the ^1^H NMR spectrum of 4 suggested the existence of a 3-methyl-but-2-en-1-yl group at C-12, which was confirmed by the COSY correlations between H-15 and H_2_-14 together with the HMBC correlations from Me-17 and 18 to C-15 (*δ*_C_ 122.4) and C-16 (*δ*_C_ 133.9), and from H_2_-14 to C-4 (*δ*_C_ 106.0) and C-12 (*δ*_C_ 138.8) (Fig. 2).

**Fig. 4:**
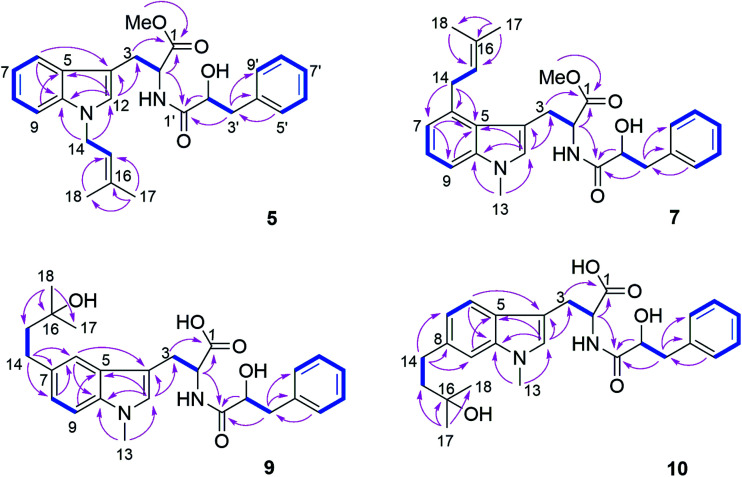
COSY and key HMBC correlations of compounds 5, 7, 9 and 10.

Compound 5 was isolated as white powder. Its molecular formula was established as C_26_H_30_O_4_N_2_ by HRESIMS. The ^1^H and ^13^C NMR spectra of 5 were similar to those of 4 except for the appearance of a singlet methine at *δ*_H_ 6.70 (H-12) and the absence of the methyl group attached to N-11. The COSY correlations between H-15 (*δ*_H_ 5.32) and H_2_-14 (*δ*_H_ 4.66) along with the HMBC correlations from H-14 to C-10 (*δ*_C_ 137.8) and C-12 (*δ*_C_ 127.6), and from both Me-17 (*δ*_H_ 1.74) and Me-18 (*δ*_H_ 1.83) to C-15 (*δ*_C_ 121.5) and C-16 (*δ*_C_ 137.1) indicated the presence of a 3-methyl-but-2-en-1-yl residue and its attachment to N-11 of compound 5 (Fig. 4).

The molecular formula of 6 was determined as C_25_H_28_O_4_N_2_, indicating the lack of a methyl group compared to 5, which was confirmed by the absence of signals of the methoxy group at C-1 compared to 5 (Tables 3 and 4). The remaining substructure of 6 was unambiguously elucidated to be identical to that of 5 by detailed analysis of 2D NMR spectra.

**Table 3:** ^13^C NMR data of compounds 6–10 (CD_3_OD, 150 MHz)

No.	6^a^	7	8	9	10
1	176.4, C	173.7, C	175.0, C	175.8, C	175.6, C
2	54.3, CH	54.7, CH	54.4, CH	54.3, CH	54.1, CH
3	28.5, CH_2_	30.3, CH_2_	30.4, CH_2_	28.3, CH_2_	28.4, CH_2_
4	110.0, C	110.1, C	110.4, C	109.4, C	109.7, C
5	129.6, C	126.8, C	127.0, C	129.8, C	127.7, C
6	119.8, CH	135.4, C	135.5, C	118.9, CH	119.8, CH
7	119.5, CH	120.7, CH	120.7, CH	134.4, C	121.0, CH
8	122.0, CH	122.7, CH	122.6, CH	123.5, CH	137.7, C
9	110.1, CH	108.5, CH	108.4, CH	110.0, CH	109.4, CH
10	137.4, C	139.3, C	139.3, C	137.1, C	138.8, C
12	127.3, CH	129.4, CH	129.3, CH	129.1, CH	128.5, CH
13		32.9, CH_3_	32.9, CH_3_	32.7, CH_3_	32.7, CH_3_
14	44.5, CH_2_	33.3, CH_2_	33.4, CH_2_	32.0, CH_2_	32.2, CH_2_
15	121.4, CH	125.5, CH	125.5, CH	47.8, CH_2_	47.8, CH_2_
16	136.5, C	132.9, C	132.9, C	71.4, C	71.4, C
17	25.5, CH_3_	25.9, CH_3_	25.9, CH_3_	29.3, CH_3_	29.3, CH_3_
18	17.8, CH_3_	18.2, CH_3_	18.3, CH_3_	29.2, CH_3_	29.2, CH_3_
1′	175.4, C	176.1, C	176.1, C	175.9, C	175.8, C
2′	73.6, CH	73.8, CH	73.8, CH	73.8, CH	73.6, CH
3′	41.3, CH_2_	41.7, CH_2_	41.7, CH_2_	41.6, CH_2_	41.4, CH_2_
4′	138.9, C	138.9, C	139.0, C	139.1, C	139.1, C
5′, 9′	130.5, CH	130.6, CH	130.6, CH	130.8, CH	130.8, CH
6′, 8′	128.8, CH	129.1, CH	129.1, CH	129.1, CH	129.1, CH
7′	127.1, CH	127.4, CH	127.4, CH	127.4, CH	127.4, CH
OCH_3_		52.7, CH_3_			

a Measured at 175 MHz.

**Table 4:** ^1^H NMR data of compounds 6–10 (CD_3_OD, 600 MHz)

No.	6^a^	7	8	9	10
2	4.66, m	4.71, dd (8.7, 5.6)	4.70, dd (8.9, 4.8)	4.67, m	4.67, m
3	3.23, dd (14.5, 6.1), 3.17, dd (14.5, 4.7)	3.38, dd (15.0, 5.6), 3.18, dd (15.0, 8.7)	3.45, dd (15.1, 4.8), 3.20, dd (15.1, 8.9)	3.21, dd (14.8, 5.8), 3.16, dd (14.8, 4.7)	3.20, dd (14.8, 5.9), 3.13, dd (14.8, 4.7)
6	7.53, d (7.8)			7.36, s	7.40, d (8.0)
7	7.01, dd (7.8, 7.1)	6.82, d (7.2)	6.82, d (7.0)		6.91, d (8.0)
8	7.11, dd (8.3, 7.1)	7.06, dd (8.6, 7.2)	7.06, dd (8.3, 7.0)	7.02, d (8.3)	
9	7.27, d (8.3)	7.16, d (8.6)	7.15, d (8.3)	7.20, d (8.3)	7.11, s
12	6.80, s	6.82, s	6.84, s	6.68, s	6.63, s
13		3.70, s	3.69, s	3.68, s	3.68, s
14	4.65, m	3.73, m	3.77, dd (16.3, 6.8), 3.73, dd (16.3, 6.8)	2.77, m	2.78, m
15	5.32, br t (6.8)	5.31, br t (6.6)	5.32, br t (6.8)	1.80, m	1.79, m
17	1.72, s	1.76, s	1.76, s	1.24, s	1.26, s
18	1.83, s	1.77, s	1.77, s	1.25, s	1.26, s
2′	4.16, dd (8.0, 3.5)	4.19, dd (7.8, 3.9)	4.18, dd (8.0, 3.8)	4.19, dd (7.8, 3.6)	4.19, dd (7.6, 3.7)
3′	2.93, dd (13.9, 3.5), 2.63, dd (13.9, 8.0)	2.88, dd (13.9, 3.9), 2.62, dd (13.9, 7.8)	2.87, dd (13.9, 3.8), 2.58, dd (13.9, 8.0)	2.95, dd (13.9, 3.6), 2.65, dd (13.9, 7.8)	2.93, dd (13.9, 3.7), 2.66, dd (13.9, 7.6)
5′, 9′	7.16, d (7.2)	7.11, d (6.9)	7.11, d (6.9)	7.17, d (7.2)	7.17, d (7.2)
6′, 8′	7.20, t (7.2)	7.14, t (6.9)	7.12, t (6.9)	7.20, t (7.2)	7.22, t (7.2)
7′	7.15, t (7.2)	7.10, t (6.9)	7.10, t (6.9)	7.15, t (7.2)	7.16, t (7.2)
OCH_3_		3.66, s			

a Measured at 700 MHz.

Compound 7 possessed the molecular formula C_27_H_32_O_4_N_2_ as deduced from the HRESIMS data. The ^1^H and ^13^C NMR spectra of 7 were similar to those of 4, revealing the presence of one monosubstituted benzene ring, an indole moiety and a 3-methyl-but-2-en-1-yl side chain. However, the appearance of an aromatic proton signal at *δ*_H_ 6.82 (H-7), which was split to a doublet, suggested that the 3-methyl-but-2-en1-yl group was substituted on the benzene ring of the tryptophan moiety. The HMBC correlation from H_2_-14 (*δ*_H_ 3.73) to C-5 (*δ*_C_ 126.8), C-6 (*δ*_C_ 135.4) and C-7 (*δ*_C_ 120.7), and from H-7 to C-5, C-9 (*δ*_C_ 108.5) and C-14 (*δ*_C_ 33.3) indicated the 3-methyl-but-2-enyl group to be located at C-6. The structure of 7 was determined by analysis of the 2D NMR spectra as shown in Fig. 4.

Compound 8 possessed the molecular formula C_26_H_30_O_4_N_2_ as established by HRESIMS, thus lacking a methyl group compared to 7. The ^1^H and ^13^C NMR spectra were almost identical to those of 7 except for the absence of signals of the methoxy group at C-1, which was further confirmed by detailed analysis of the 2D NMR data of 8.

Compound 9 had the molecular formula C_26_H_32_O_5_N_2_ as determined by HRESIMS, corresponding to 12 degrees of unsaturation. The ^1^H NMR spectra of 9 (Table 4) revealed the presence of a 1,2,4-trisubstituted benzene ring and a monosubstituted benzene ring, suggesting a substituent at C-7 or C-8 of the indole moiety. A 3-hydroxy-3-methylbutyl moiety was established based on the correlation between H_2_-14 (*δ*_H_ 2.77) and H_2_-15 (*δ*_H_ 1.80) together with the HMBC correlations from Me-17 (*δ*_H_ 1.24) and Me-18 (*δ*_H_ 1.25) to C-16 (*δ*_C_ 71.4) and C-15 (*δ*_C_ 47.8). In addition, the HMBC correlation from H_2_-14 to C-6 (*δ*_C_ 118.9), C-7 (*δ*_C_ 134.4) and C-8 (*δ*_C_ 123.5), from H-6 (*δ*_H_ 7.36) to C-4 (*δ*_C_ 109.4), C-8, C-10 (*δ*_C_ 137.1) and C-14 (*δ*_C_ 32.0), and from H-8 (*δ*_H_ 7.02) to C-6, C-10 and C-14 indicated the 3-hydroxy-3-methylbutyl moiety to be attached at C-7 (Fig. 4).

Compound 10 shared the same molecular formula as 9. The ^1^H NMR data of 10 resembled those of compound 9 except for the chemical shifts of the aromatic protons of the indole moiety. The HMBC correlation from H_2_-14 (*δ*_H_ 2.78) to C-7 (*δ*_C_ 121.0), C-8 (*δ*_C_ 137.7) and C-9 (*δ*_C_ 109.4), from H-7 (*δ*_H_ 6.91) to C-5 (*δ*_C_ 127.7), C-9 and C-14 (*δ*_C_ 32.3), and from H-9 (*δ*_H_ 7.11) to C-5, C-7 and C-14 confirmed that the 3-hydroxy-3-methylbutyl moiety was located at the C-8 position. The remaining substructure of 10 was the same as that of 9 as confirmed by detailed analysis of 2D NMR spectra of 10 (Fig. 4).

Compounds 3–10 share the same core structure with compounds 1 and 2, for which the absolute configuration had been assigned through X-ray analysis. Hence it is concluded on biogenetic terms that the absolute configuration of the former compounds is also 2*S*, 2′*S*. Compounds 11 and 12 were determined to have 2*S* absolute configuration by Marfey's reaction. Based on the close biogenetic similarity, the absolute configuration at C-2′ of the latter two compounds is assumed to be *S*.

**Fig. 5:**
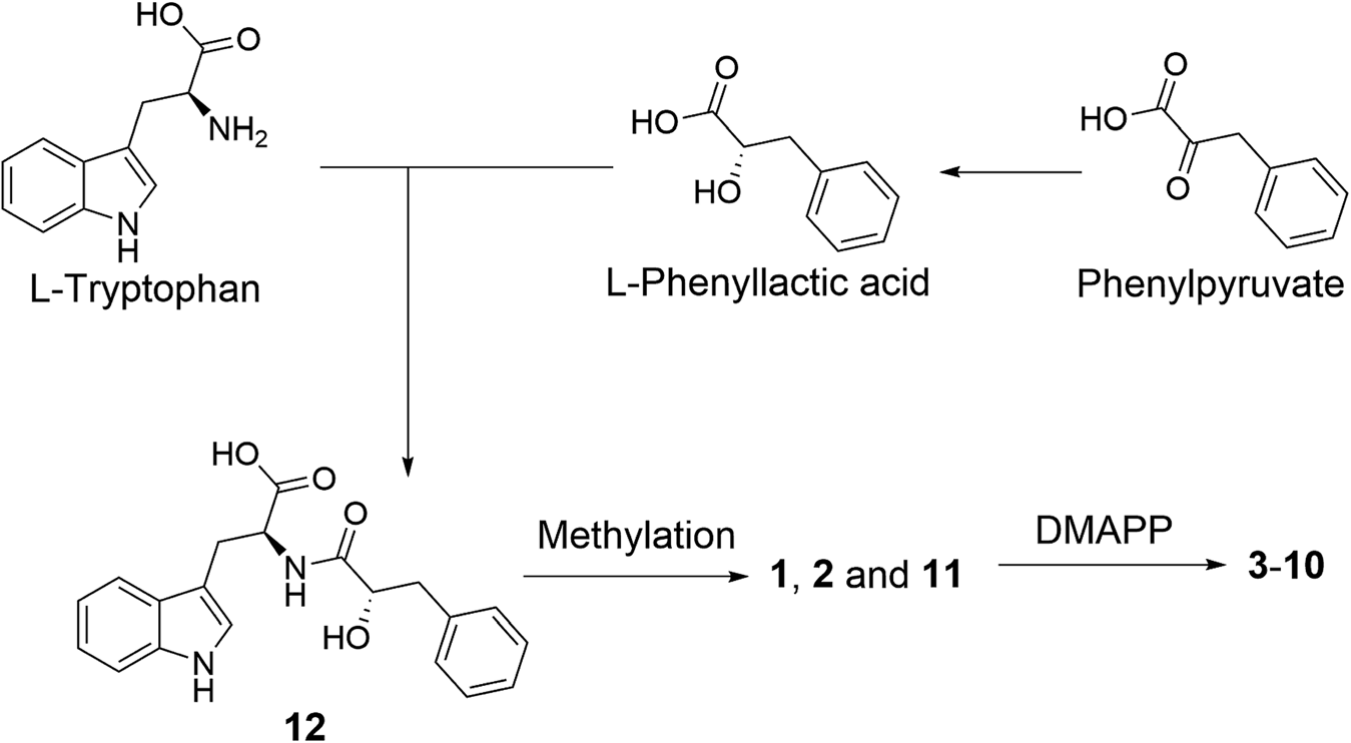
Plausible biosynthesis of substituted l-tryptophan-l-phenyllactic acid conjugates from *A. aculeatus*.

A plausible biosynthetic pathway of the substituted l-tryptophan-l-phenyllactic acid conjugates obtained from *A. aculeatus* is proposed to start from l-tryptophan and phenylpyruvate. The important intermediate 12 is suggested to be formed by a condensation reaction between l-tryptophan and l-phenyllactic acid. The indole metabolites isolated in this study could be produced by further methylation of 12 and prenylation of the indole nucleus of tryptophan as shown in Fig. 5.

All isolated compounds (1–25) were evaluated for their cytotoxicity against the L5178Y mouse lymphoma cell line. Secalonic acids D and F (19 and 20), asperdichrome (21) and RF 3192C (22) showed cytotoxicity with IC_50_ values of 3.4, 1.4, 7.3 and 23.7 μM, respectively, whereas the remaining compounds proved to be inactive when assayed at a dose of 10 μg mL^−1^.

The results of the OSMAC experiments indicated that substituted l-tryptophan-l-phenyllactic acid conjugates were induced by nitrate but not by sodium in the medium. In the absence of nitrogen sources favoured by the fungus including ammonium and glutamine, fungi are able to use secondary nitrogen sources such as nitrate, purines, urea, amines and amides *etc.*, which is commonly known as nitrogen metabolite repression (NMR).^30^ In fungi, the activity of nitrogen regulators for derepression of NMR genes, which also affect secondary metabolites formation, is regulated by the intracellular nitrogen status and extracellular nitrogen availability.^31,32^ For example, the main GATA transcriptional regulator of nitrogen metabolism AreA accumulates in the nucleus in *Fusarium graminearum* with nitrate as sole nitrogen source, which is required for activation of the nitrate assimilation system including the nitrate reductase genes.^33^ Thus, the biosynthesis of fungal secondary metabolites can be affected by the quality and quantity of the nitrogen sources. For example, 67% of secondary metabolites silent gene clusters of *Fusarium fujikuroi* were expressed based on the modification of nitrogen sources.^34^ Furthermore, the natural product beauvericin was accumulated by *Fusarium oxysporum* by utilizing nitrate as sole nitrogen source.^35^ In this study, the production of substituted l-tryptophan-l-phenyllactic acid conjugates was stimulated by the activation of the nitrate assimilation system in *A. aculeatus* due to the presence of sodium nitrate in medium.

The substituted l-tryptophan-l-phenyllactic acid conjugates identified in this study showed no cytotoxic or antibacterial activity. However, the two known compounds (11 and 12) were claimed as plant growth regulators in a patent and showed pronounced rooting promoting effect.^36^ It may hence be hypothesized that a high concentration of nitrate in host plants, as simulated in this study by addition of sodium nitrate to solid rice medium, may induce the production of plant growth stimulating indole metabolites of the endophytic fungus *A. aculeatus*.^37^ This could lead to an increased growth and production of biomass by the host plant. In return, the fungus could receive nutrients, water, minerals and nitrogen from its host. Further studies will be necessary to evaluate this hypothesis.

## Experimental section

### General procedures

A Jasco P-2000 polarimeter was used to measure the optical rotation. 1D and 2D NMR spectra were recorded on Bruker Avance DMX 600 or 700 NMR spectrometers. Chemical shifts were referenced to the solvent residual peaks. Mass spectra were recorded with a LC-MS HP1100 Agilent Finnigan LCQ Deca XP Thermoquest and HRESIMS were measured with a UHR-QTOF maXis 4G (Bruker Daltonics) mass spectrometer. HPLC analysis was performed on a Dionex 3000 RS system coupled with an Ultimate 3000 pump and a photodiode array detector (DAD 300RS). The analytical column (125 × 4 mm) was prefilled with Eurosphere-10 C_18_ (Knauer, Germany), and the following gradient solvent system was used: 0 min (10% MeOH), 5 min (10% MeOH), 35 min (100% MeOH), and 45 min (100% MeOH). Semi-preparative HPLC was performed using a Merck Hitachi HPLC System (UV detector L-7400; pump L-7100; Eurosphere-100 C_18_, 300 × 8 mm, Knauer) with MeOH–H_2_O as mobile phase and a flow rate of 5.0 mL min^−1^. Column chromatography was carried out using Merck MN silica gel 60 M (0.04–0.063 mm). TLC plates with silica gel F_254_ (Merck) were used to monitor and collect fractions under detection at 254 and 366 nm. Distilled and spectral-grade solvents were used for column chromatography and spectroscopic measurements, respectively.

### Fungal material and cultivation

*A. aculeatus* was isolated from leaves of *Carica papaya* collected in Awka in Nigeria and was identified by DNA amplification, sequencing of ITS region and by comparing with GenBank data (GeneBank accession no. KX137846) following standard procedures.^38^

The fungal strain was grown on solid rice medium (100 g rice and 100 mL distilled water autoclaved) in ten Erlenmeyer flasks (1 L each) at 22 °C under static conditions for 14 days. The OSMAC experiments were performed on rice medium containing either 3.5% NaCl, 3.5% NaBr, 3.5% NaI, 1% NaF, 3.5% NaNO_3_, 3.5% NH_4_Cl, 3.5% (NH_4_)_2_SO_4_ or 3.5% NH_4_OAc under static conditions until they reached their stationary phase of growth (16 days except for rice media spiked with 1% NaF or 3.5% NH_4_OAc where the fungus failed to grow).

### Extraction and isolation

Fungal cultures were extracted with EtOAc followed by evaporation under reduced pressure. Initial purification of the EtOAc extract (12.5 g) of the fungal culture fermented on rice was performed by partitioning between *n*-hexane and 90% aqueous MeOH. The 90% aqueous MeOH phase (9.2 g) was centrifuged and then fractionated by vacuum liquid chromatography on reversed-phase silica gel using a gradient elution of H_2_O–MeOH (10 : 90 – 0 : 100) to give 10 fractions (Fr.1 to Fr.10).

Fr.2 (152 mg) was chromatographed on a Sephadex LH-20 column with MeOH followed by further purification using semi-preparative HPLC to give 17 (1.5 mg), 18 (7.0 mg) and 24 (0.5 mg). Compound 19 (30.2 mg) was obtained from Fr.3 (835 mg) by recrystallization. Part of Fr.3 was purified by semi-preparative HPLC to give 20 (20.0 mg). Fr.4 (208 mg) was subjected to a Sephadex LH-20 column with MeOH to give two subfractions (Fr.4-1 and Fr.4-2). Fr.4-1 was further purified by a silica gel column with DCM/MeOH as mobile phase to give 15 (3.8 mg). Compound 1 (13.1 mg) was obtained by recrystallization from Fr.4-2. Fr.5 (160 mg) was separated by a Sephadex LH-20 column followed by purification using semi-preparative HPLC to yield 14 (0.7 mg) and 21 (8.2 mg). Compound 11 (3.2 mg), 22 (1.2 mg) and 25 (2.4 mg) were obtained from Fr.6 (54 mg) by semi-preparative HPLC. Fr.7 (101 mg) was subjected to a Sephadex LH-20 column with MeOH as mobile phase and then purified by semi-preparative HPLC to give 23 (2.0 mg) and 16 (0.8 mg).

The fungal cultures from the OSMAC experiments that were grown on rice medium containing different salts were extracted with EtOAc (2 × 500 mL) followed by solvent evaporation under reduced pressure. The obtained crude extracts were analyzed by HPLC. The EtOAc extracts (28.6 g) of fungal cultures that had been grown on rice medium (30 flasks) after adding 3.5% NaNO_3_ were dissolved in MeOH and then subjected to vacuum filtering. The obtained MeOH solution was evaporated under reduced pressure and fractionated by vacuum liquid chromatography on silica gel using a gradient elution of *n*-hexane–EtOAc to give 24 fractions (Fr.N1 to Fr.N24). Fr.N19 (221 mg) was separated by a Sephadex LH-20 column with MeOH as mobile phase followed by semi-preparative HPLC to give 3 (3.5 mg) and 4 (15.0 mg). Fr.N22 (465 mg) was separated by a Sephadex LH-20 column with MeOH as mobile phase to give 3 fractions. Fr.N22-2 (54 mg) was further purified by semi-preparative HPLC to give 1 (10.1 mg), 2 (10.4 mg), 11 (3.5 mg), 12 (3.4 mg) and 13 (2.5 mg). Fr.N22-3 (380 mg) was further purified by a silica gel column with DCM–MeOH as mobile phase followed by semi-preparative HPLC to give 5 (2.4 mg), 6 (0.9 mg), 7 (4.8 mg), 8 (4.7 mg), 9 (1.2 mg) and 10 (1.2 mg).

Aculeatine A (1), white needle crystals; [*α*]^20^_D_ +23 (*c* 0.25, MeOH); UV (MeOH): *λ*_max_ 221, 288 nm; HRESIMS *m*/*z* 381.1810 [M + H]^+^ (calcd 381.1809 for C_22_H_25_O_4_N_2_); ^1^H and ^13^C NMR data see Tables 1 and 2.

Aculeatine B (2), colorless needle crystals; [*α*]^20^_D_ +3 (*c* 0.40, MeOH); UV (MeOH): *λ*_max_ 227, 288 nm; HRESIMS *m*/*z* 367.1653 [M + H]^+^ (calcd 367.1652 for C_21_H_23_O_4_N_2_); ^1^H and ^13^C NMR data see Tables 1 and 2.

Aculeatine C (3), white amorphous powder; [*α*]^20^_D_ −54 (*c* 0.70, MeOH); UV (MeOH): *λ*_max_ 227, 288 nm; HRESIMS *m*/*z* 449.2439 [M + H]^+^ (calcd 449.2435 for C_27_H_33_O_4_N_2_); ^1^H and ^13^C NMR data see Tables 1 and 2.

Aculeatine D (4), white amorphous solid; [*α*]^20^_D_ −47 (*c* 1.7, MeOH); UV (MeOH): *λ*_max_ 233, 286 nm; HRESIMS *m*/*z* 449.2435 [M + H]^+^ (calcd 449.2435 for C_27_H_33_O_4_N_2_); ^1^H and ^13^C NMR data see Tables 1 and 2.

Aculeatine E (5), white amorphous powder; [*α*]^20^_D_ −2 (*c* 0.48, MeOH); UV (MeOH): *λ*_max_ 228, 287 nm; HRESIMS *m*/*z* 435.2281 [M + H]^+^ (calcd 435.2278 for C_26_H_31_O_4_N_2_); ^1^H and ^13^C NMR data see Tables 1 and 2.

Aculeatine F (6), white amorphous solid; [*α*]^20^_D_ −11 (*c* 0.20, MeOH); UV (MeOH): *λ*_max_ 228, 288 nm; HRESIMS *m*/*z* 421.2122 [M + H]^+^ (calcd 421.2122 for C_25_H_29_O_4_N_2_); ^1^H and ^13^C NMR data see Tables 3 and 4.

Aculeatine G (7), white amorphous powder; [*α*]^20^_D_ −24 (*c* 0.20, MeOH); UV (MeOH): *λ*_max_ 225, 291 nm; HRESIMS *m*/*z* 449.2435 [M + H]^+^ (calcd 435.2435 for C_27_H_33_O_4_N_2_) and *m*/*z* 471.2252 [M + Na]^+^ (calcd 471.2254 for C_27_H_32_O_4_N_2_Na); ^1^H and ^13^C NMR data see Tables 3 and 4.

Aculeatine H (8), white amorphous powder; [*α*]^20^_D_ −41 (*c* 0.94, MeOH); UV (MeOH): *λ*_max_ 226, 291 nm; HRESIMS *m*/*z* 435.2282 [M + H]^+^ (calcd 435.2278 for C_26_H_31_O_4_N_2_) and *m*/*z* 457.2099 [M + Na]^+^ (calcd 457.2098 for C_26_H_30_O_4_N_2_Na); ^1^H and ^13^C NMR data see Tables 3 and 4.

Aculeatine I (9), white amorphous solid; [*α*]^20^_D_ −35 (*c* 0.24, MeOH); UV (MeOH): *λ*_max_ 225, 292 nm; HRESIMS *m*/*z* 453.2383 [M + H]^+^ (calcd 453.2384 for C_26_H_33_O_5_N_2_); ^1^H and ^13^C NMR data see Tables 3 and 4.

Aculeatine J (10), light yellow amorphous solid; [*α*]^20^_D_ −19 (*c* 0.24, MeOH); UV (MeOH): *λ*_max_ 226, 287 nm; HRESIMS *m*/*z* 453.2380 [M + H]^+^ (calcd 453.2384 for C_26_H_33_O_5_N_2_) and *m*/*z* 475.2201 [M + Na]^+^ (calcd 475.2203 for C_26_H_32_O_5_N_2_Na); ^1^H and ^13^C NMR data see Tables 3 and 4.

### X-ray crystallographic analysis of compounds 1 and 2

#### Crystallization conditions

Suitable single crystals of 1 and 2 were obtained by slow evaporation from methanol solution and selected under a polarized light microscope. *Data collection*: compounds 1 and 2 were measured on a Bruker Kappa APEX2 CCD diffractometer with micro focus tube using Cu-Kα radiation (*λ* = 1.54178 Å). APEX2 was used for data collection,^39^ SAINT for cell refinement and data reduction,^39^ and SADABS for experimental absorption correction.^40^ SHELXT was used for the structure solution by intrinsic phasing,^41^ SHELXL-2017 was used for refinement by full-matrix least-squares on *F*^2^.^42^ The hydrogen atoms were positioned geometrically (with C–H = 0.95 Å for aromatic CH, 1.00 Å for tertiary CH, 0.99 Å for CH_2_ and 0.98 Å for CH_3_). The refinement was carried out using riding models (AFIX 43, 13, 23, 137, respectively), with *U*_iso_(H) = 1.2*U*_eq_(CH, CH_2_) and 1.5*U*_eq_(CH_3_). The hydrogen atoms in the hydroxy and amine groups were refined with *U*_iso_(H) = 1.5*U*_eq_(O/N). The hydrogen atoms in the solvent methanol molecule in 2 were refined with *U*_iso_(H) = 1.5*U*_eq_(O).

The absolute structures of compound 1 and 2 were determined using anomalous dispersion from Cu-Kα radiation, resulting in Flack parameters of −0.14(10) (1) and 0.00(3) (2) using Parsons quotient method.^43^ Due to a rather high Flack parameter for 1, its absolute structure was determined using likelihood methods.^44^ DIAMOND was used for the drawing of all graphics.^45^ PLATON for Windows was used for the analyses of hydrogen bonds and CH–π interactions.^46^ The structural data for 2 has been deposited in the Cambridge Crystallographic Data Center (CCDC no. 1589955). Crystals of 1 did not diffract beyond *θ* = 44.9° (*cf.* desired 67.7°) for Cu-Kα radiation, resulting in only 1538 total (1484 observed with *I* > 2*σ*(*I*)) reflections *versus* 263 parameters for anisotropic refinement. Therefore the cif did not meet the requirements for publication and refinement data of compound 1 is only given in the ESI.†

#### Crystal data of 1

C_22_H_24_N_2_O_4_, *M* = 380.43, orthorhombic system, space group *P*2_1_2_1_2_1_, *a* = 11.8099(7) Å, *b* = 11.8099(7) Å, *c* = 27.4081(17) Å, *V* = 1936.7(2) Å^3^, *Z* = 4, *D*_calc_ = 1.305 g cm^−3^, crystal size 0.12 × 0.03 × 0.03 mm^3^, *μ*(Cu-Kα) = 0.73 mm^−1^, 3.2° < *θ* < 44.9°, *N*_t_ = 16 751, *N* = 1538 (*R*_int_ = 0.051), *R*_1_ = 0.022, w*R*_2_ = 0.053, *S* = 1.12, Flack parameter = −0.14(10), Hooft parameter = −0.09(8), probability for correct absolute structure *P*2 = 1.000.

#### Crystal data of 2

C_21_H_22_N_2_O_4_·CH_4_O, *M* = 398.45, monoclinic system, space group *C*2, *a* = 23.52(2) Å, *b* = 5.994(5) Å, *c* = 15.843(13) Å, *V* = 2063(3) Å^3^, *Z* = 4, *D*_calc_ = 1.283 g cm^−3^, crystal size 0.20 × 0.10 × 0.05 mm^3^, *μ*(Cu-Kα) = 0.75 mm^−1^, 5.9° < *θ* < 67.5°, *N*_t_ = 11 796, *N* = 3416 (*R*_int_ = 0.027), *R*_1_ = 0.025, w*R*_2_ = 0.067, *S* = 1.09, Flack parameter = 0.00(3), Hooft parameter = 0.00(3), probability for correct absolute structure *P*2 = 1.000.

### Marfey's reaction for compounds 11 and 12

Compounds 11 and 12 (0.5 mg) were hydrolyzed with 2 mL 6 M HCl containing 0.4% β-mercaptoethanol at 110 °C for 24 h. The hydrolysate was evaporated to dryness and treated separately with 4 M NaOH at room temperature for 4 h. 4 M HCl was used to adjust the pH to 4. The above resulting solutions were evaporated until complete elimination of HCl and then resuspended in 50 μL H_2_O. To 25 μL of each resulting solutions was added 50 μL FDAA (1% 1-fluoro-2-4-dinitrophenyl-5-l-alanine amide in acetone) and 10 μL NaHCO_3_. The reaction tubes were covered with an aluminum paper and heated over a hot plate at 40 °C for 1 h. After cooling to room temperature, 5 μL of 2 M HCl was added and then evaporated to dryness. The residue was dissolved in 500 μL MeOH. l-Tryptophan and d-tryptophan were treated separately with FDAA in the same manner. The analysis of FDAA derivatives were carried out using HPLC and LC-MS by comparison of the retention time and molecular weight.

### Cytotoxicity assay

Cytotoxicity was tested against the L5178Y mouse lymphoma cell line (European Collection of Authenticated Cell Cultures, Catalogue no. 87111908) using the MTT method as described before.^11^ Kahalalide F was used as positive control with a IC_50_ value of 4.3 μM.

## Conflicts of interest

There are no conflicts to declare.

## Supplementary Material

RA-008-C8RA00200B-s001

RA-008-C8RA00200B-s002
