# Supplementary material for: Substituted l-tryptophan-l-phenyllactic acid conjugates produced by an endophytic fungus Aspergillus aculeatus using an OSMAC approach
Source: RSC Adv. 2018 Feb 19;8(14):7863–72. doi: 10.1039/c8ra00200b (PMC9078508; doi:10.1039/c8ra00200b)
Supplement: RA-008-C8RA00200B-s001 [file RA-008-C8RA00200B-s001.pdf]

Electronic Supplementary Material (ESI) for RSC Advances.
This journal is © The Royal Society of Chemistry 2018

Supplementary information

Substituted L-tryptophan-L-phenyllactic acid conjugates produced by an endophytic fungus *Aspergillus aculeatus* using an OSMAC approach

Hao Wang [a], Peter M. Eze [b], Simon-Patrick Höfert [c], Christoph Janiak [c], Rudolf Hartmann [d], Festus B.C. Okoye [e], Charles O. Esimone [b], Raha S. Orfali [f], Haofu Dai [g], Zhen Liu [a,*], Peter Proksch [a,*]

[a]*Institute of Pharmaceutical Biology and Biotechnology, Heinrich-Heine-University Düsseldorf, Universitätsstrasse 1, 40225 Düsseldorf, Germany*

[b]*Department of Pharmaceutical Microbiology and Biotechnology, Faculty of Pharmaceutical Sciences, Nnamdi Azikiwe University, Awka, Nigeria*

[c]*Institute of Inorganic and Structural Chemistry, Heinrich-Heine-University Düsseldorf, Universitätsstrasse 1, 40225 Düsseldorf, Germany*

[d]*Institute of Complex Systems: Structural Biochemistry, Forschungszentrum Juelich, Wilhelm-Johnen-Straße, 52428 Juelich, Germany*

[e]*Department of Pharmaceutical and Medicinal Chemistry, Faculty of Pharmaceutical Sciences, Nnamdi Azikiwe University, Awka, Nigeria*

[f]*Department of Pharmacognosy, Faculty of Pharmacy, King Saud University, Riyadh, Saudi Arabia*

[g]*Key Laboratory of Biology and Genetic Resources of Tropical Crops, Ministry of Agriculture, Institute of Tropical Bioscience and Biotechnology, Chinese Academy of Tropical Agricultural Sciences, Haikou 571101, China*

Table of Contents

Figure S1. UV spectrum of **1**....4
Figure S2. HRESIMS of **1**....4
Figure S3. ^{1}H NMR (600 MHz, methanol-d_4) spectrum of **1**....5
Figure S4. ^{13}C NMR (150 MHz, methanol-d_4) spectrum of **1**....5
Figure S5. ^{1}H-^{1}H COSY (600 MHz, methanol-d_4) spectrum of **1**....6
Figure S6. HSQC (600 and 150 MHz, methanol-d_4) spectrum of **1**....6
Figure S7. HMBC (600 and 150 MHz, methanol-d_4) spectrum of **1**....7
Figure S8. UV spectrum of **2**....7
Figure S9. HRESIMS of **2**....8
Figure S10.^{1}H NMR (600 MHz, methanol-d_4) spectrum of **2**....8
Figure S11. ^{13}C NMR (150 MHz, methanol-d_4) spectrum of **2**....9
Figure S12. ^{1}H-^{1}H COSY (600 MHz, methanol-d_4) spectrum of **2**....9
Figure S13. HSQC (600 and 150 MHz, methanol-d_4) spectrum of **2**....10
Figure S14. HMBC (600 and 150 MHz, methanol-d_4) spectrum of **2**....10
Figure S15. UV spectrum of **3**....11
Figure S16. HRESIMS of **3**....11
Figure S17. ^{1}H NMR (600 MHz, methanol-d_4) spectrum of **3**....12
Figure S18. ^{13}C NMR (150 MHz, methanol-d_4) spectrum of **3**....12
Figure S19. ^{1}H-^{1}H COSY (600 MHz, methanol-d_4) spectrum of **3**....13
Figure S20. HSQC (600 and 150 MHz, methanol-d_4) spectrum of **3**....13
Figure S21. HMBC (600 and 150 MHz, methanol-d_4) spectrum of **3**....14
Figure S22. UV spectrum of **4**....14
Figure S23. HRESIMS of **4**....15
Figure S24. ^{1}H NMR (600 MHz, methanol-d_4) spectrum of **4**....15
Figure S25. ^{13}C NMR (150 MHz, methanol-d_4) spectrum of **4**....16
Figure S26. ^{1}H-^{1}H COSY (600 MHz, methanol-d_4) spectrum of **4**....16
Figure S27. HSQC (600 and 150 MHz, methanol-d_4) spectrum of **4**....17
Figure S28. HMBC (600 and 150 MHz, methanol-d_4) spectrum of **4**....17
Figure S29. UV spectrum of **5**....18
Figure S30. HRESIMS of **5**....18
Figure S31. ^{1}H NMR (600 MHz, methanol-d_4) spectrum of **5**....19
Figure S32. ^{13}C NMR (150 MHz, methanol-d_4) spectrum of **5**....19
Figure S33. ^{1}H-^{1}H COSY (600 MHz, methanol-d_4) spectrum of **5**....20
Figure S34. HSQC (600 and 150 MHz, methanol-d_4) spectrum of **5**....20
Figure S35. HMBC (600 and 150 MHz, methanol-d_4) spectrum of **5**....21
Figure S36. UV spectrum of **6**....21
Figure S37. HRESIMS of **6**....22
Figure S38. ^{1}H NMR (700 MHz, methanol-d_4) spectrum of **6**....22
Figure S39. ^{1}H-^{1}H COSY (700 MHz, methanol-d_4) spectrum of **6**....23
Figure S40. HSQC (700 and 175 MHz, methanol-d_4) spectrum of **6**....23
Figure S41. HMBC (700 and 175 MHz, methanol-d_4) spectrum of **6**....24
Figure S42. UV spectrum of **7**....24

Figure S43. HRESIMS of **7**....25
Figure S44. ^{1}H NMR (600 MHz, methanol-d_4) spectrum of **7**....25
Figure S45. ^{13}C NMR (150 MHz, methanol-d_4) spectrum of **7**....26
Figure S46. ^{1}H-^{1}H COSY (600 MHz, methanol-d_4) spectrum of **7**....26
Figure S47. HSQC (600 and 150 MHz, methanol-d_4) spectrum of **7**....27
Figure S48. HMBC (600 and 150 MHz, methanol-d_4) spectrum of **7**....27
Figure S49. UV spectrum of **8**....28
Figure S50. HRESIMS of **8**....28
Figure S51. ^{1}H NMR (600 MHz, methanol-d_4) spectrum of **8**....29
Figure S52. ^{13}C NMR (150 MHz, methanol-d_4) spectrum of **8**....29
Figure S53. ^{1}H-^{1}H COSY (600 MHz, methanol-d_4) spectrum of **8**....30
Figure S54. HSQC (600 and 150 MHz, methanol-d_4) spectrum of **8**....30
Figure S55. HMBC (600 and 150 MHz, methanol-d_4) spectrum of **8**....31
Figure S56. UV spectrum of **9**....31
Figure S57. HRESIMS of **9**....32
Figure S58. ^{1}H NMR (600 MHz, methanol-d_4) spectrum of **9**....32
Figure S59. ^{1}H-^{1}H COSY (600 MHz, methanol-d_4) spectrum of **9**....33
Figure S60. HSQC (600 and 150 MHz, methanol-d_4) spectrum of **9**....33
Figure S61. HMBC (600 and 150 MHz, methanol-d_4) spectrum of **9**....34
Figure S62. UV spectrum of **10**....34
Figure S63. HRESIMS of **10**....35
Figure S64. ^{1}H NMR (600 MHz, methanol-d_4) spectrum of **10**....35
Figure S65. ^{1}H-^{1}H COSY (600 MHz, methanol-d_4) spectrum of **10**....36
Figure S66. HSQC (600 and 150 MHz, methanol-d_4) spectrum of **10**....36
Figure S67. HMBC (600 and 150 MHz, methanol-d_4) spectrum of **10**....37
Results of X-ray analysis of compound 1....37
Results of X-ray analysis of compound 2....45

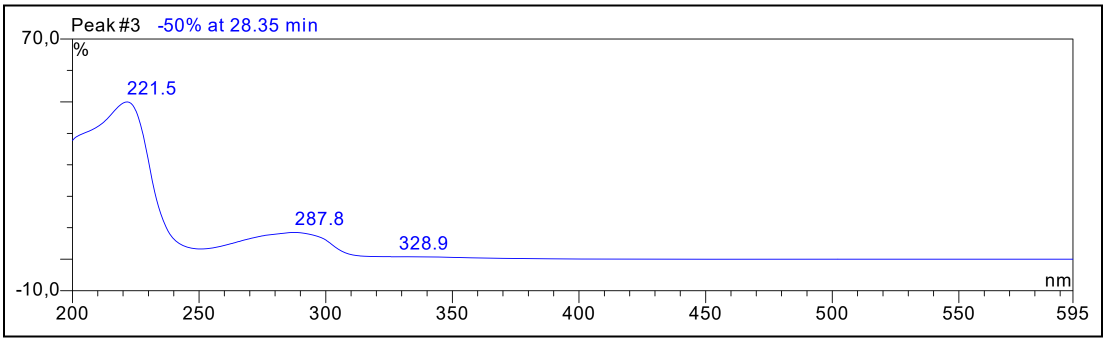

Figure S1. UV spectrum of **1**

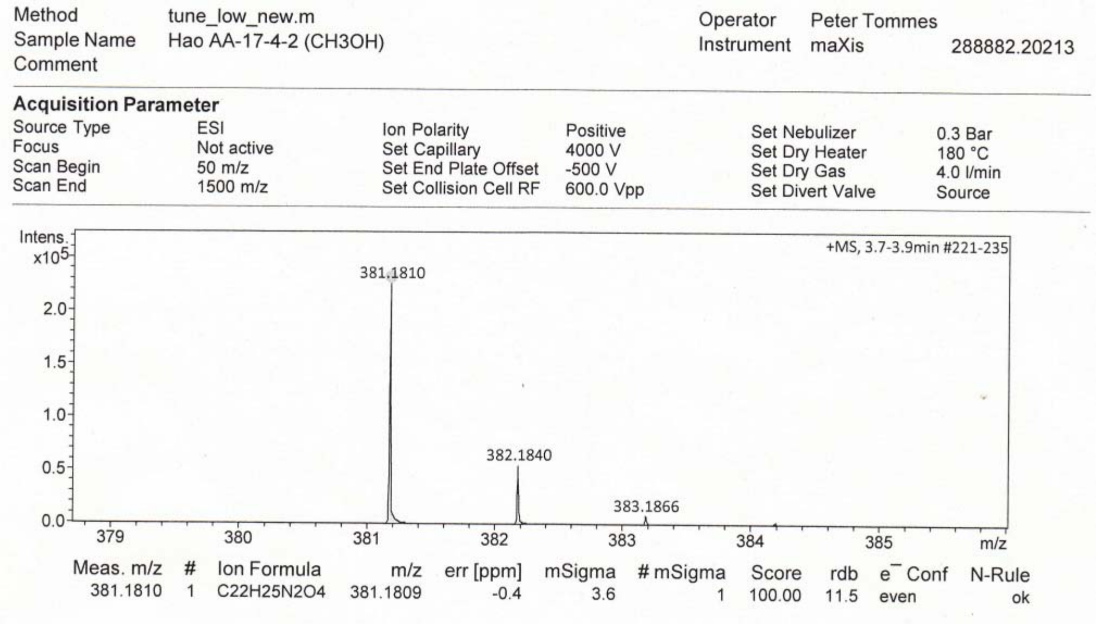

Figure S2. HRESIMS of **1**

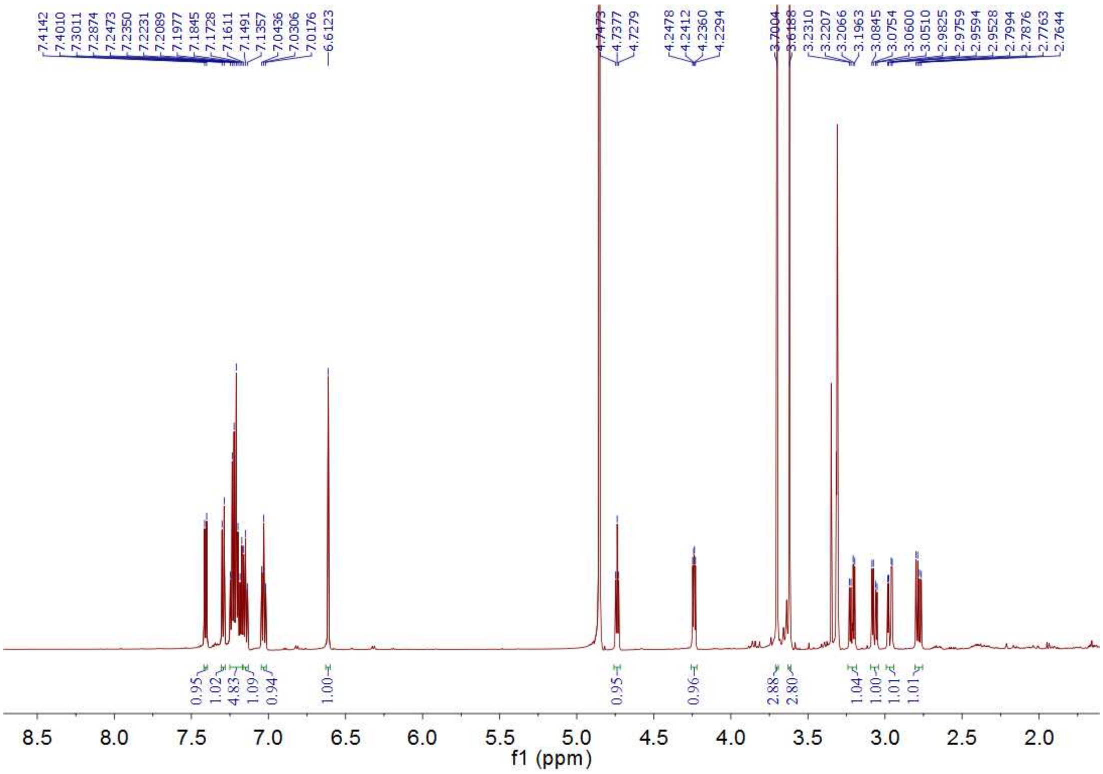

Figure S3. ^{1}H NMR (600 MHz, methanol-d_4) spectrum of **1**

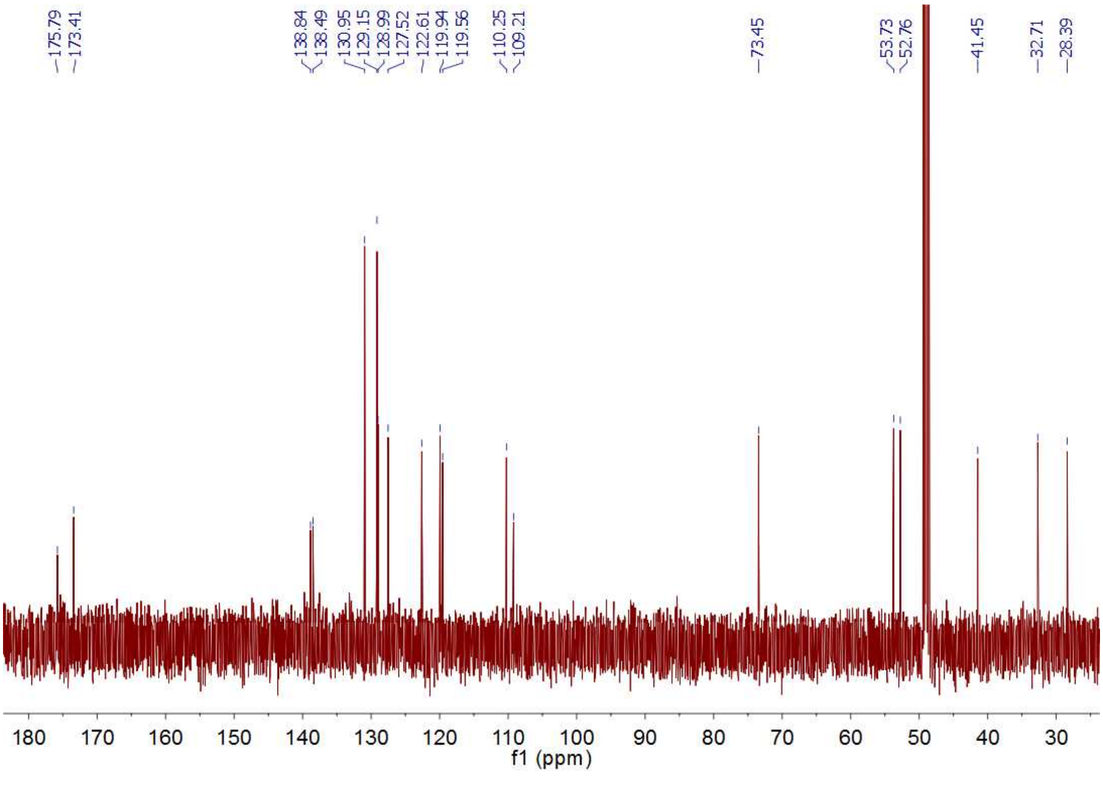

Figure S4. ^{13}C NMR (150 MHz, methanol-d_4) spectrum of **1**

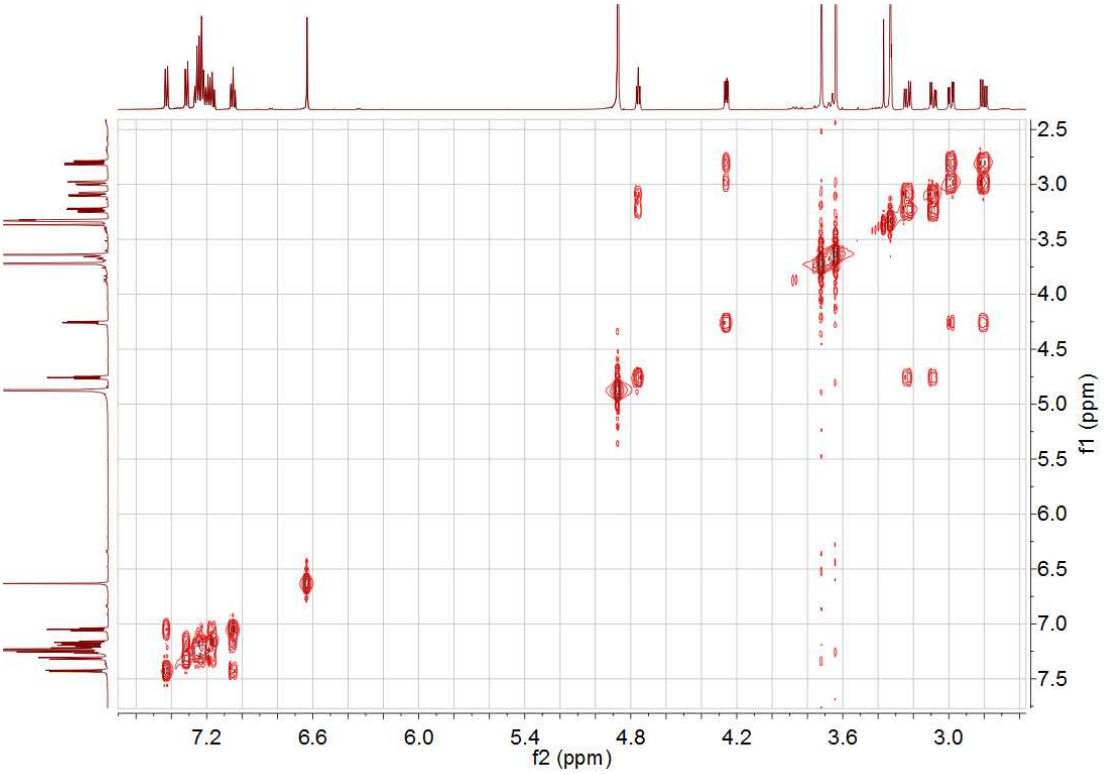

Figure S5. ^{1}H-^{1}H COSY (600 MHz, methanol-d_4) spectrum of **1**

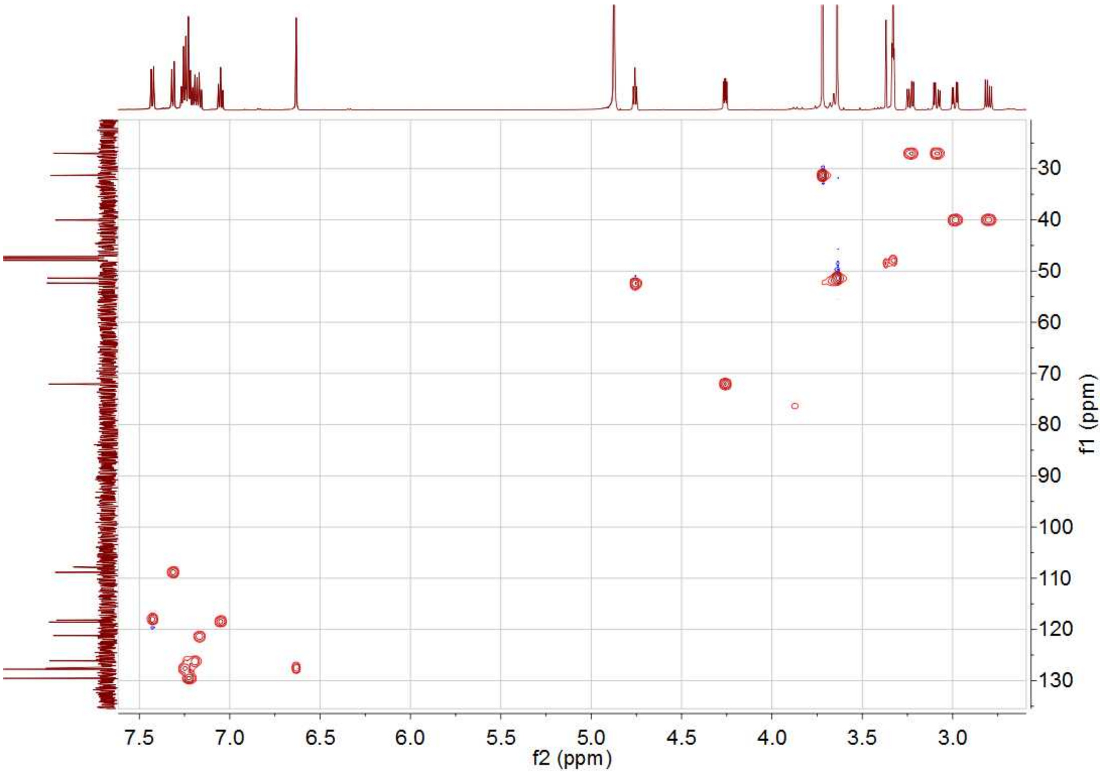

Figure S6. HSQC (600 and 150 MHz, methanol-d_4) spectrum of **1**

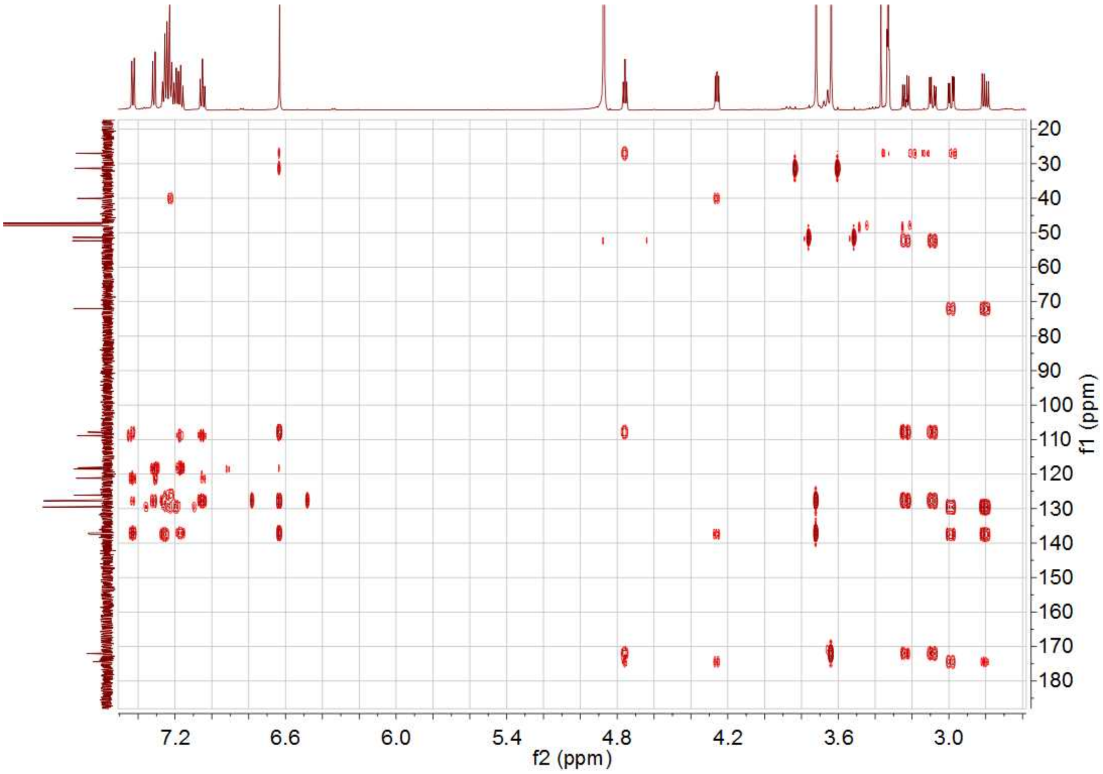

Figure S7. HMBC (600 and 150 MHz, methanol-d_4) spectrum of **1**

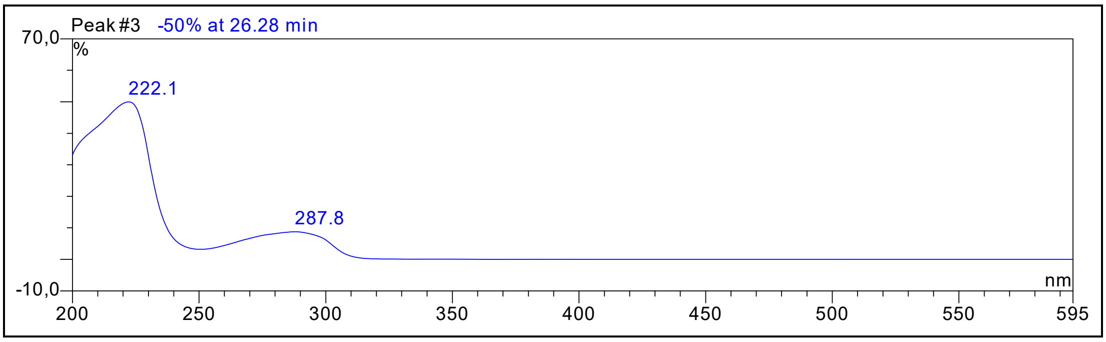

Figure S8. UV spectrum of **2**

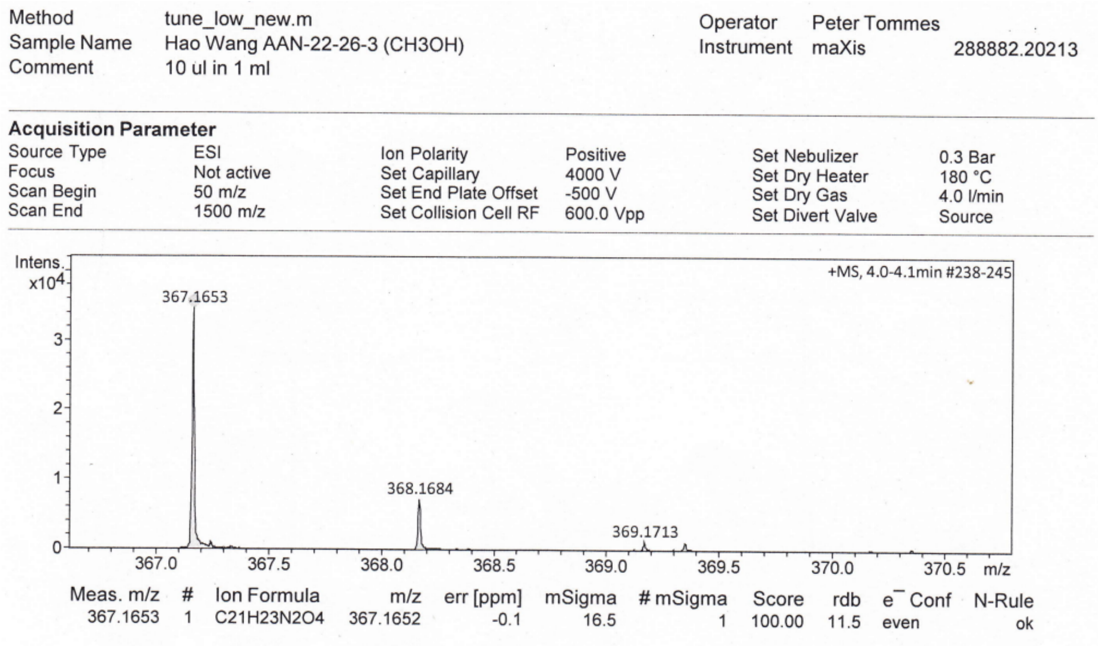

Figure S9. HRESIMS of **2**

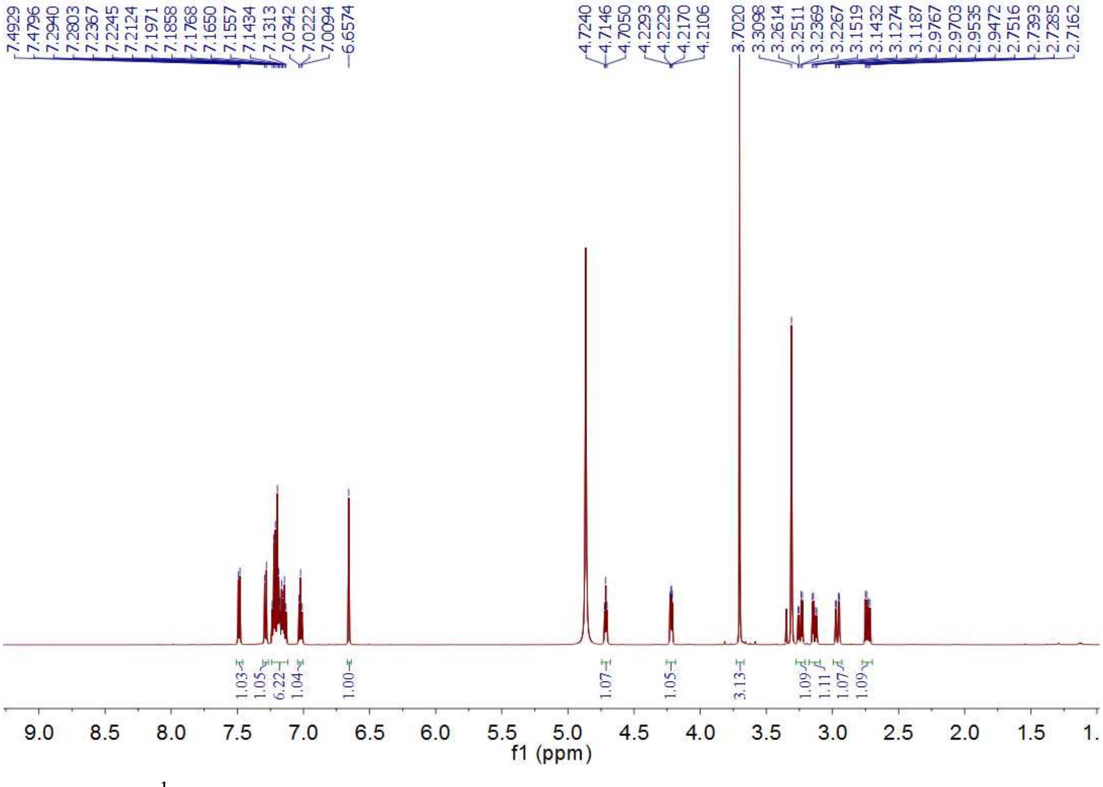

Figure S10.^{1}H NMR (600 MHz, methanol-d_4) spectrum of **2**

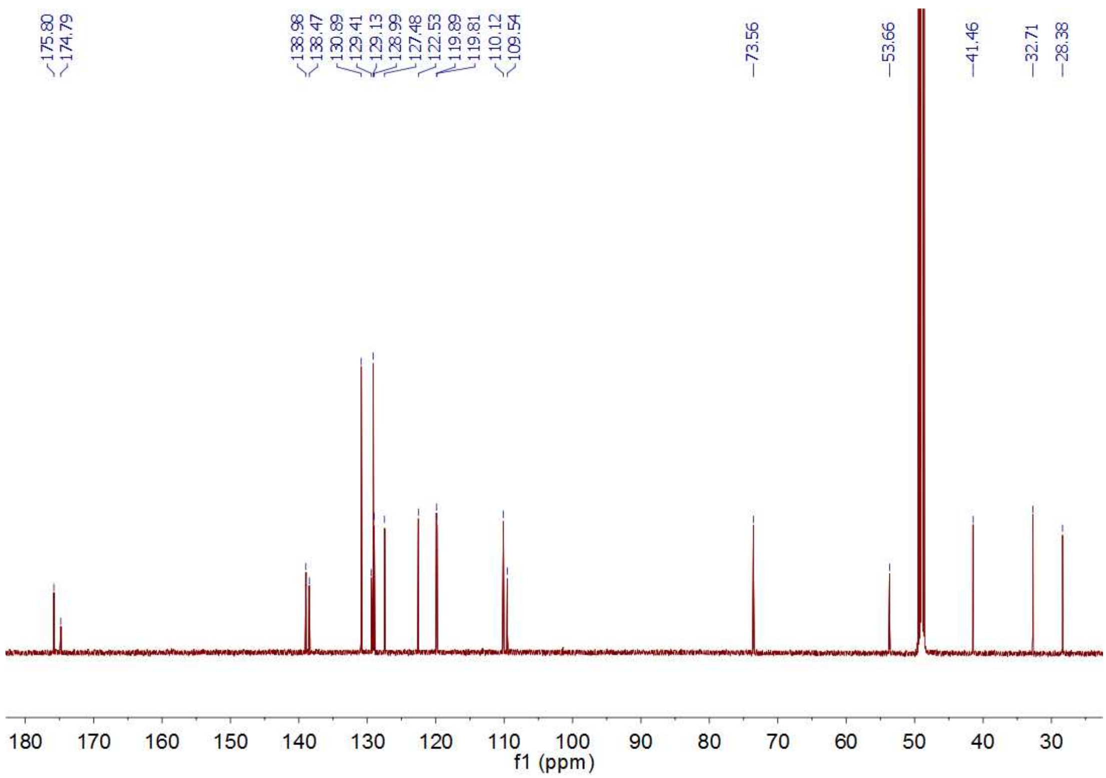

Figure S11. ^{13}C NMR (150 MHz, methanol-d_4) spectrum of **2**

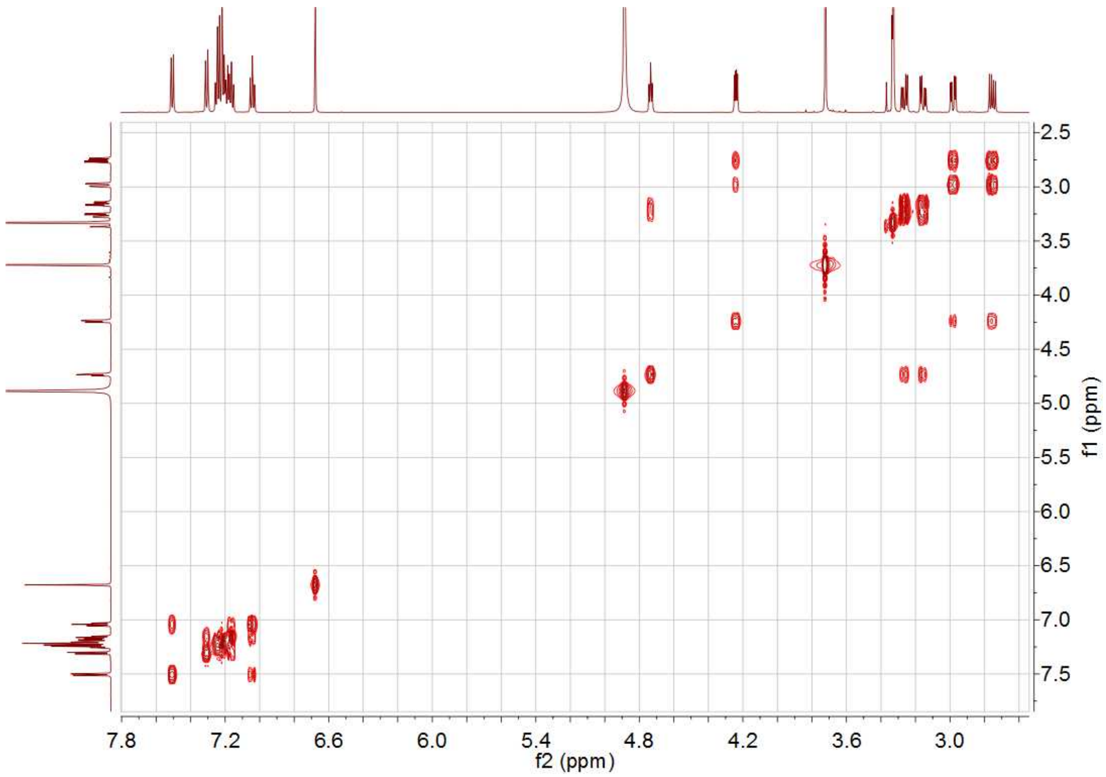

Figure S12. ^{1}H-^{1}H COSY (600 MHz, methanol-d_4) spectrum of **2**

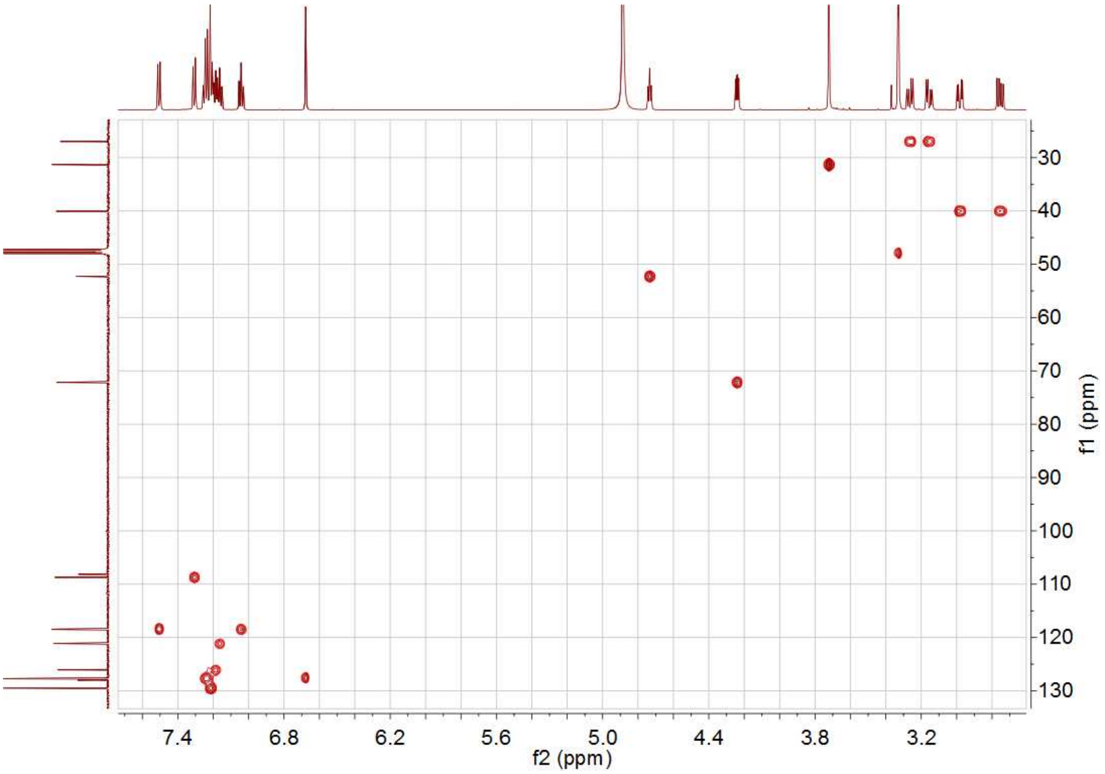

Figure S13. HSQC (600 and 150 MHz, methanol-d_4) spectrum of **2**

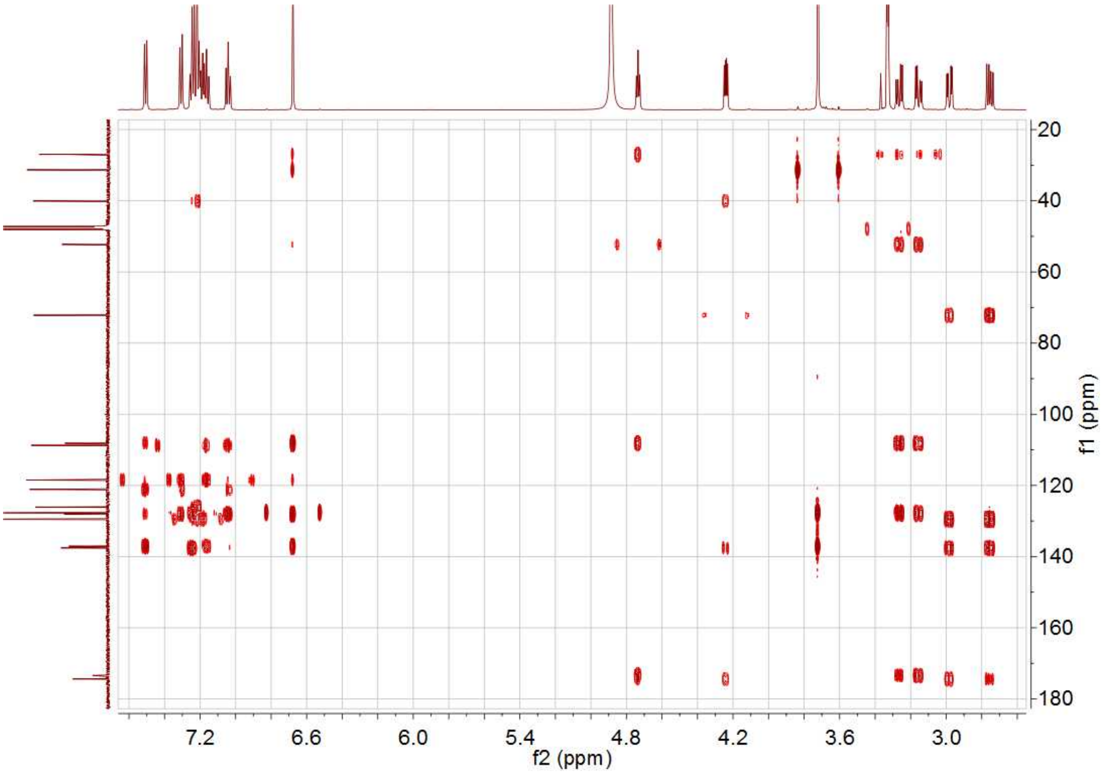

Figure S14. HMBC (600 and 150 MHz, methanol-d_4) spectrum of **2**

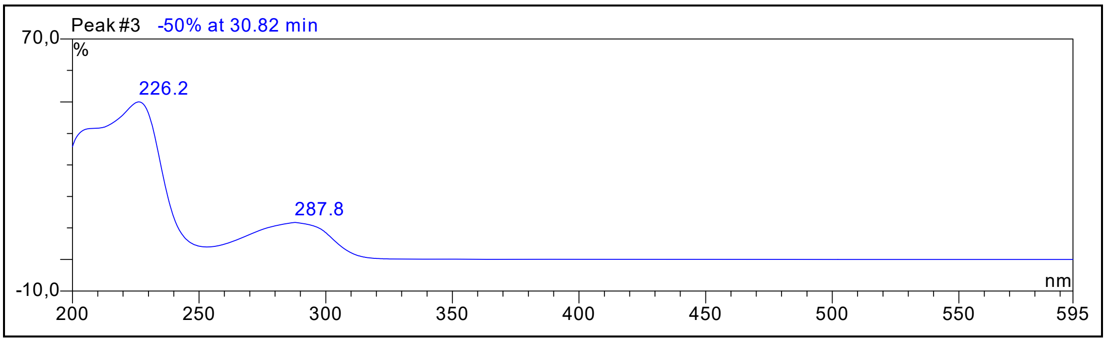

Figure S15. UV spectrum of **3**

Method	tune_low_new.m	Operator	Peter Tommes
Sample Name	Hao Wang AAN-19-5 (CH3OH)	Instrument	maXis 288882.20213
Comment			

Acquisition Parameter

Source Type	ESI	Ion Polarity	Positive	Set Nebulizer	0.3 Bar
Focus	Not active	Set Capillary	4000 V	Set Dry Heater	180 °C
Scan Begin	50 m/z	Set End Plate Offset	-500 V	Set Dry Gas	4.0 l/min
Scan End	1500 m/z	Set Collision Cell RF	600.0 Vpp	Set Divert Valve	Source

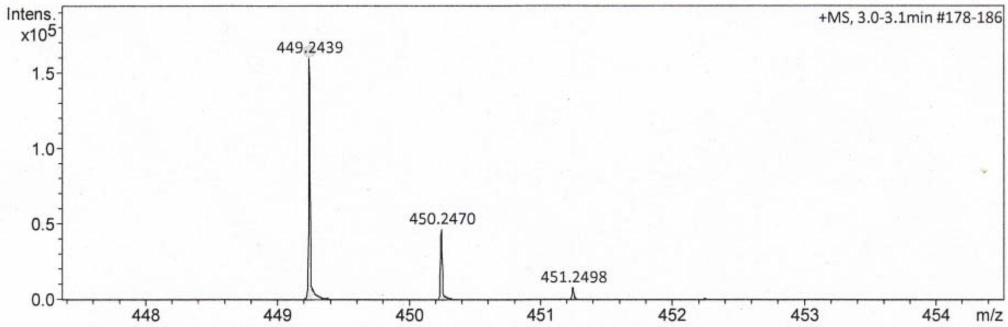

Meas. m/z	#	Ion Formula	m/z	err [ppm]	mSigma	# mSigma	Score	rdb	e¯ Conf	N-Rule
449.2439	1	C27H33N2O4	449.2435	-1.0	8.0	1	100.00	12.5	even	ok
	2	C28H29N6	449.2448	2.0	21.3	2	61.33	17.5	even	ok

Figure S16. HRESIMS of **3**

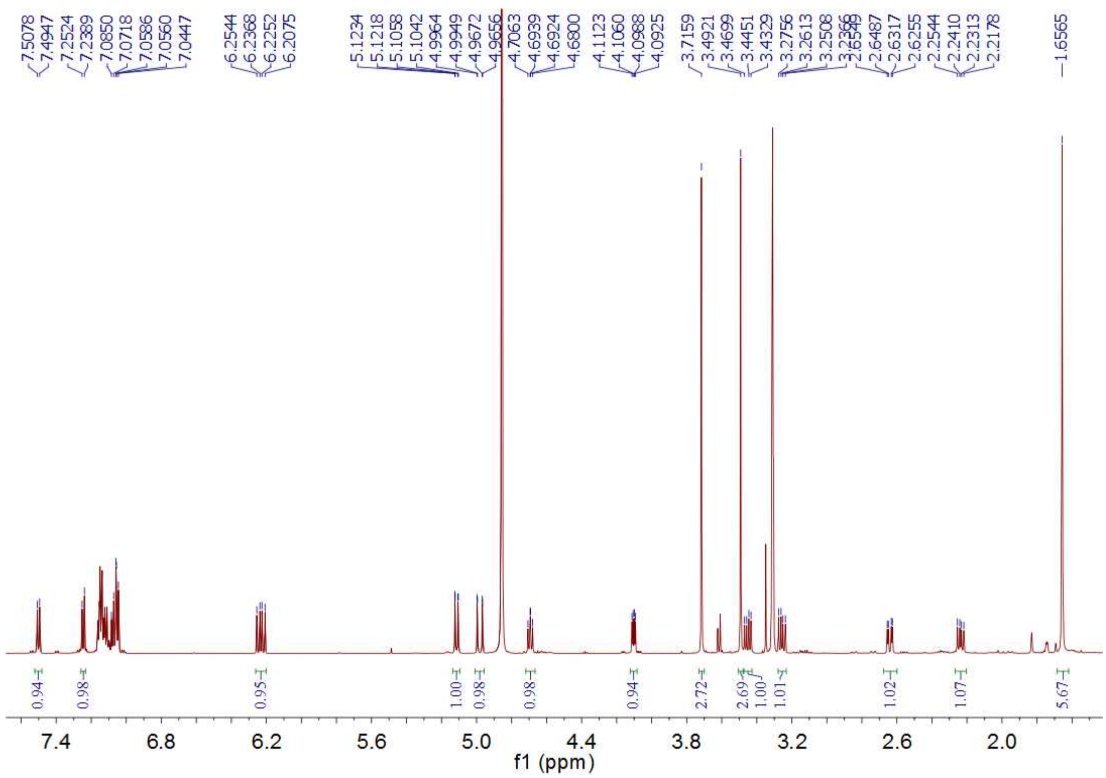

Figure S17. ^{1}H NMR (600 MHz, methanol-d_4) spectrum of **3**

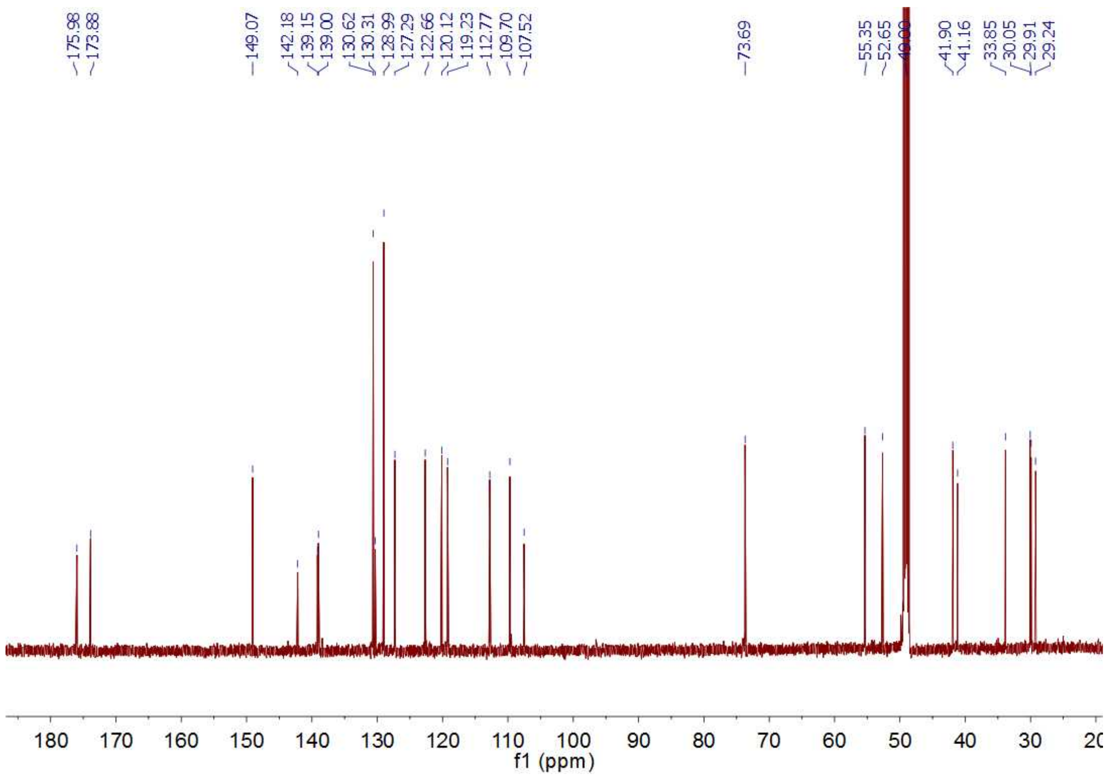

Figure S18. ^{13}C NMR (150 MHz, methanol-d_4) spectrum of **3**

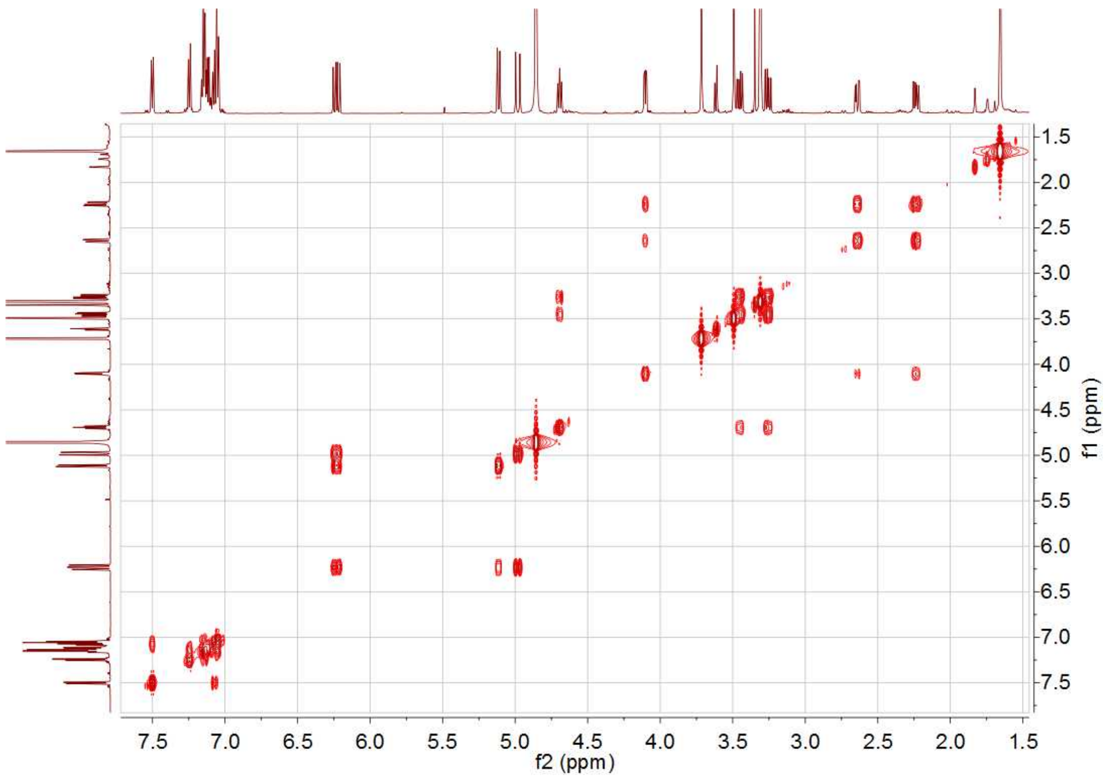

Figure S19. ^{1}H-^{1}H COSY (600 MHz, methanol-d_4) spectrum of **3**

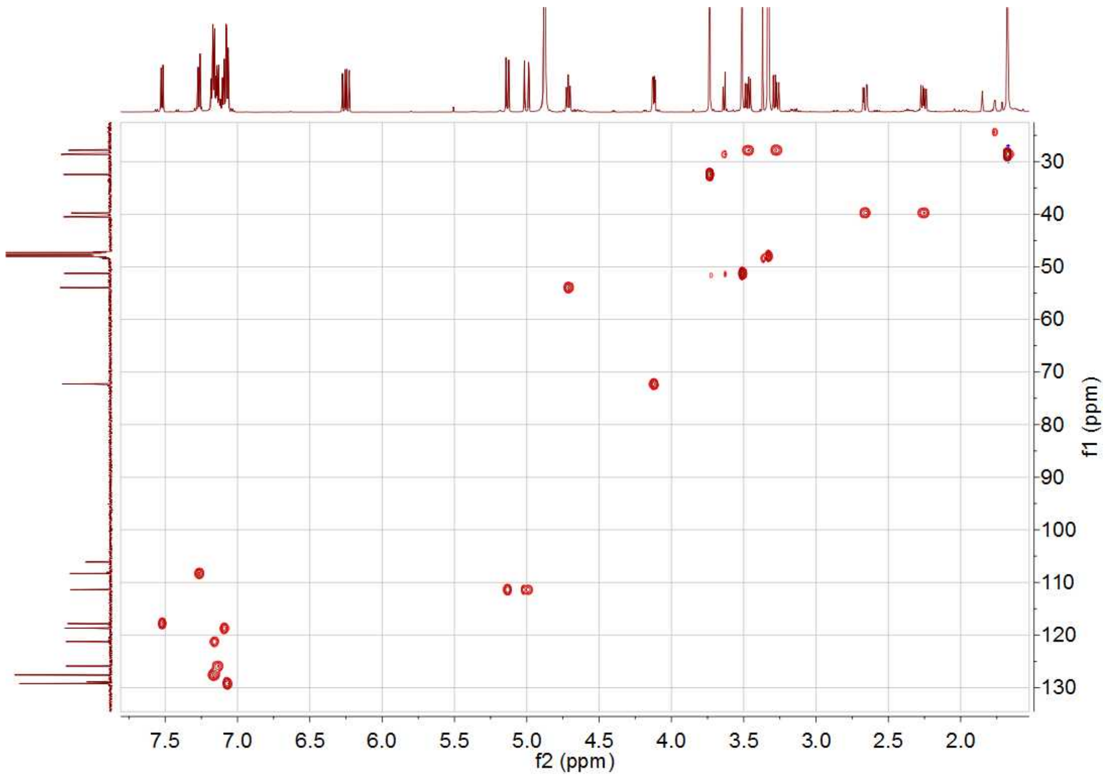

Figure S20. HSQC (600 and 150 MHz, methanol-d_4) spectrum of **3**

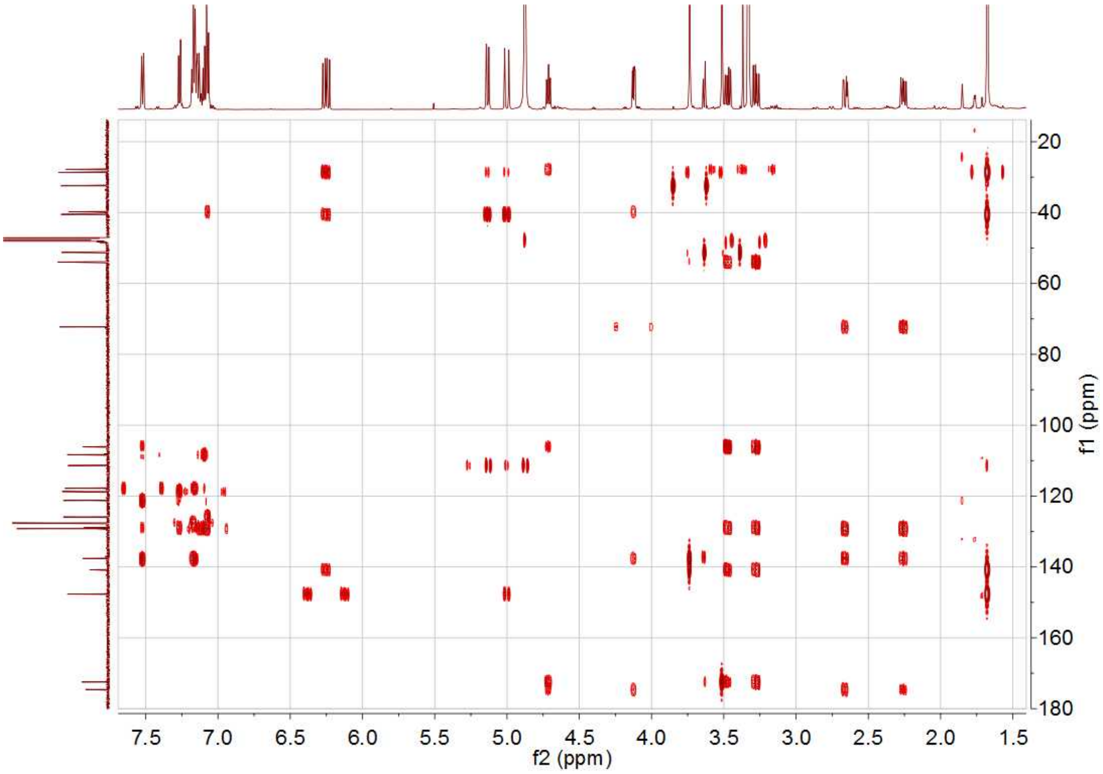

Figure S21. HMBC (600 and 150 MHz, methanol-d_4) spectrum of **3**

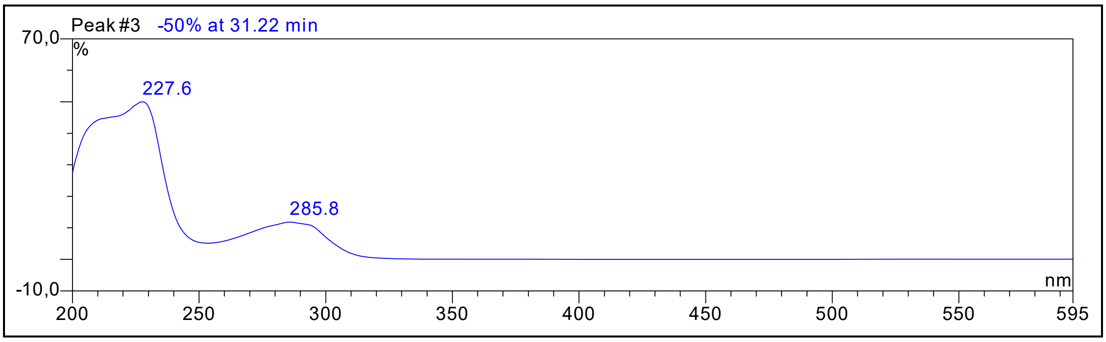

Figure S22. UV spectrum of **4**

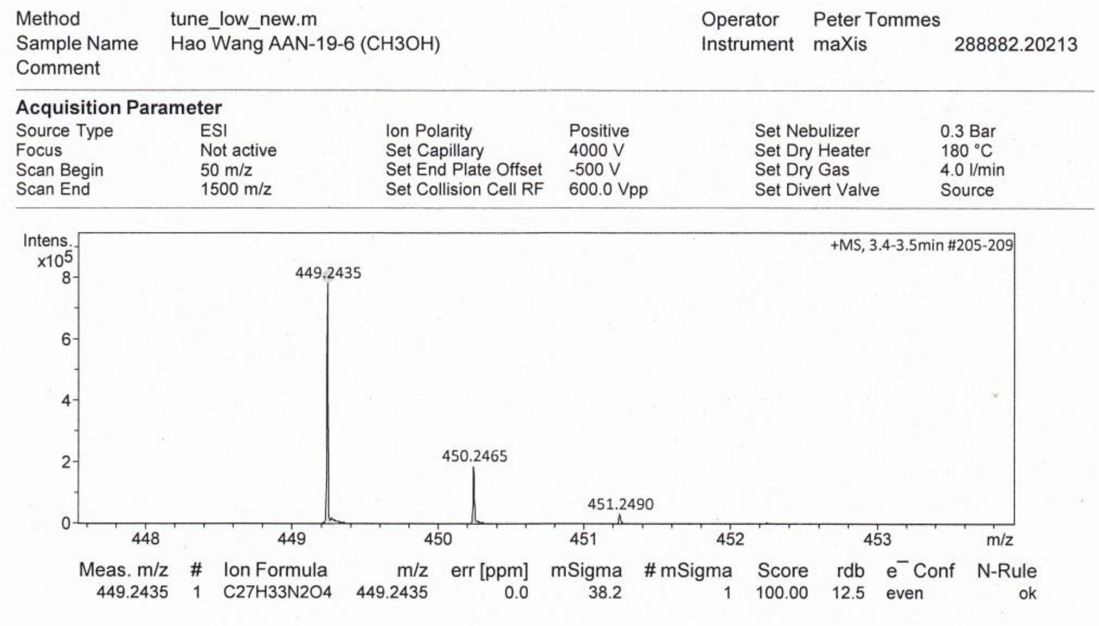

Figure S23. HRESIMS of **4**

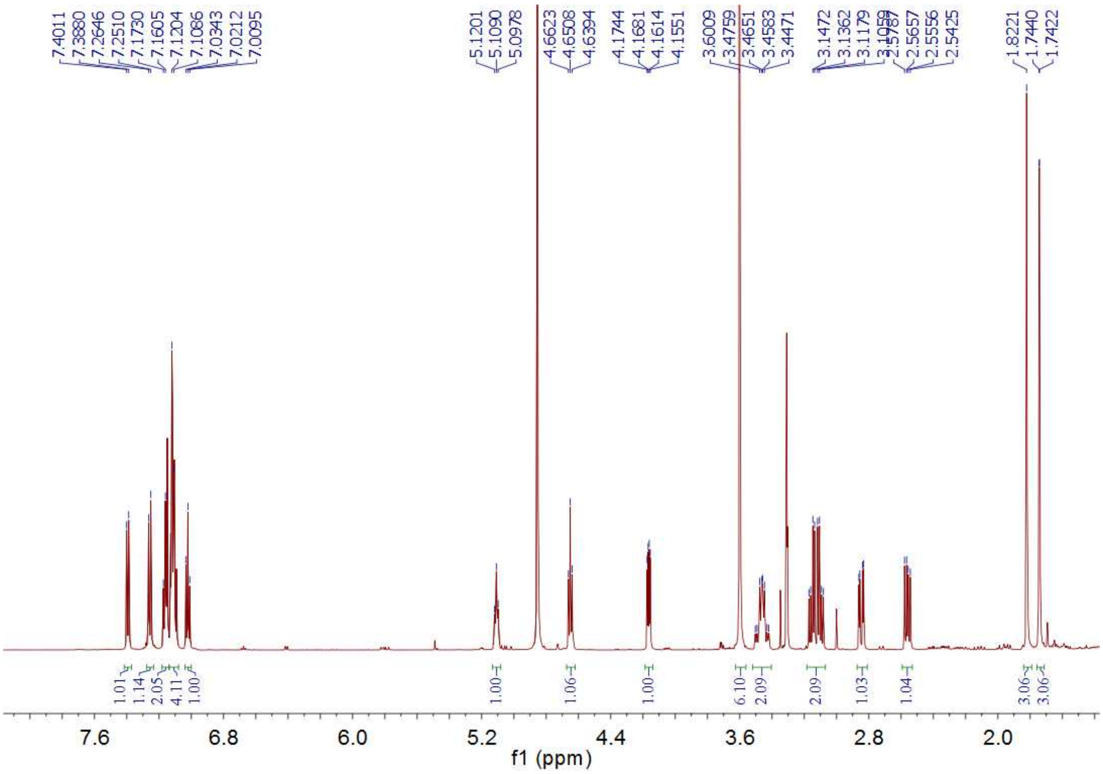

Figure S24. ^{1}H NMR (600 MHz, methanol-d_4) spectrum of **4**

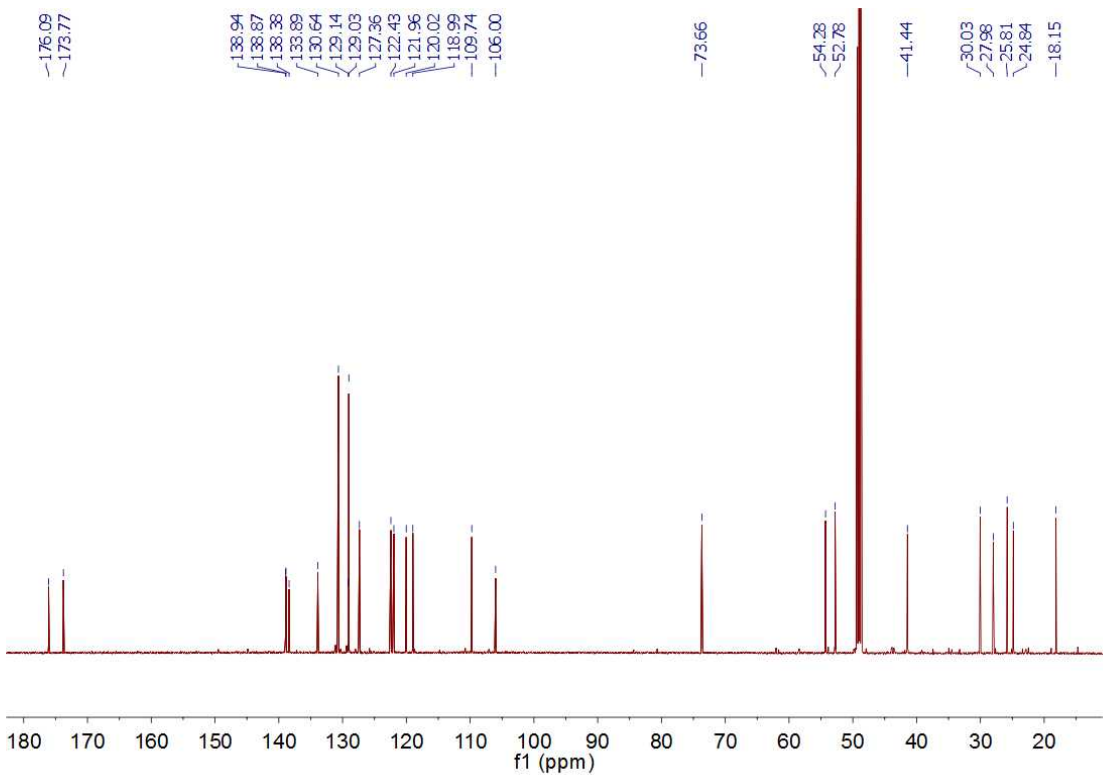

Figure S25. ^{13}C NMR (150 MHz, methanol-d_4) spectrum of **4**

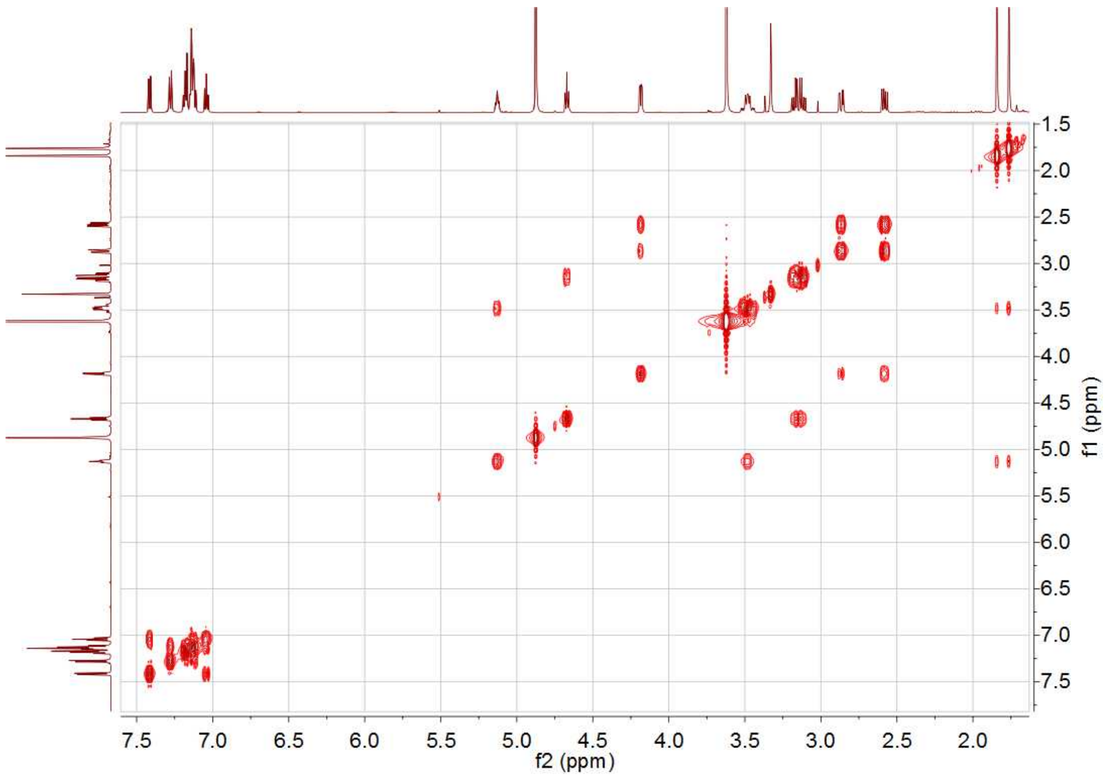

Figure S26. ^{1}H-^{1}H COSY (600 MHz, methanol-d_4) spectrum of **4**

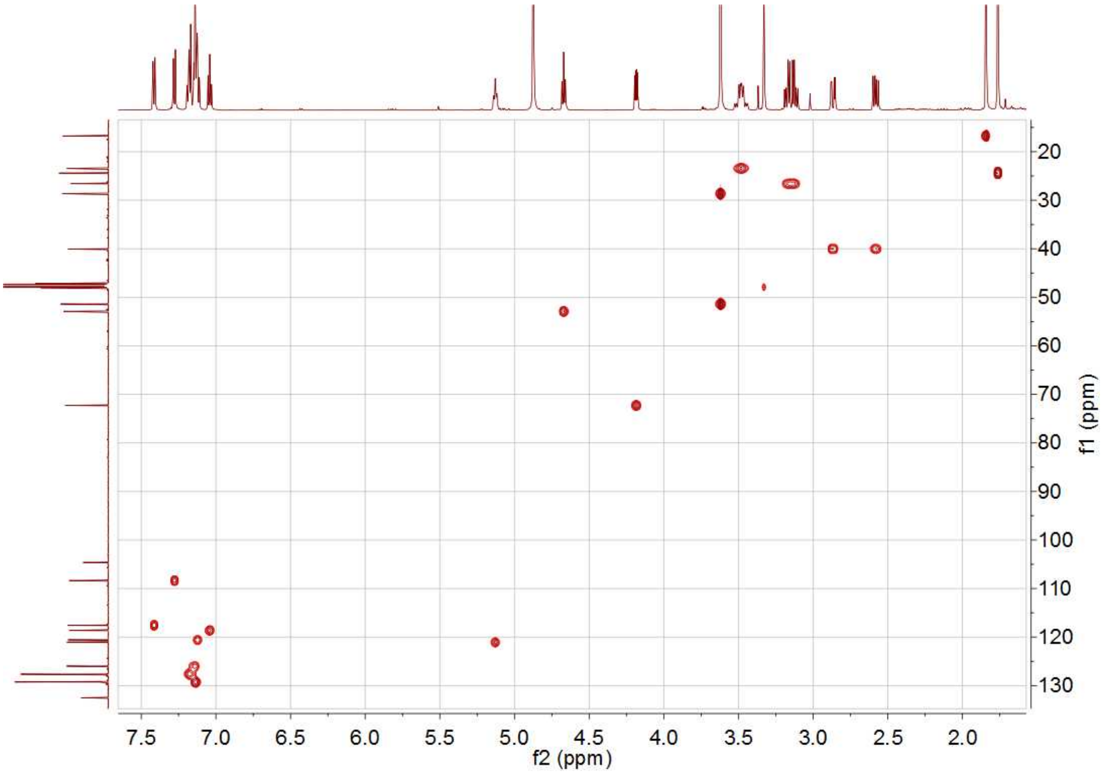

Figure S27. HSQC (600 and 150 MHz, methanol-d_4) spectrum of **4**

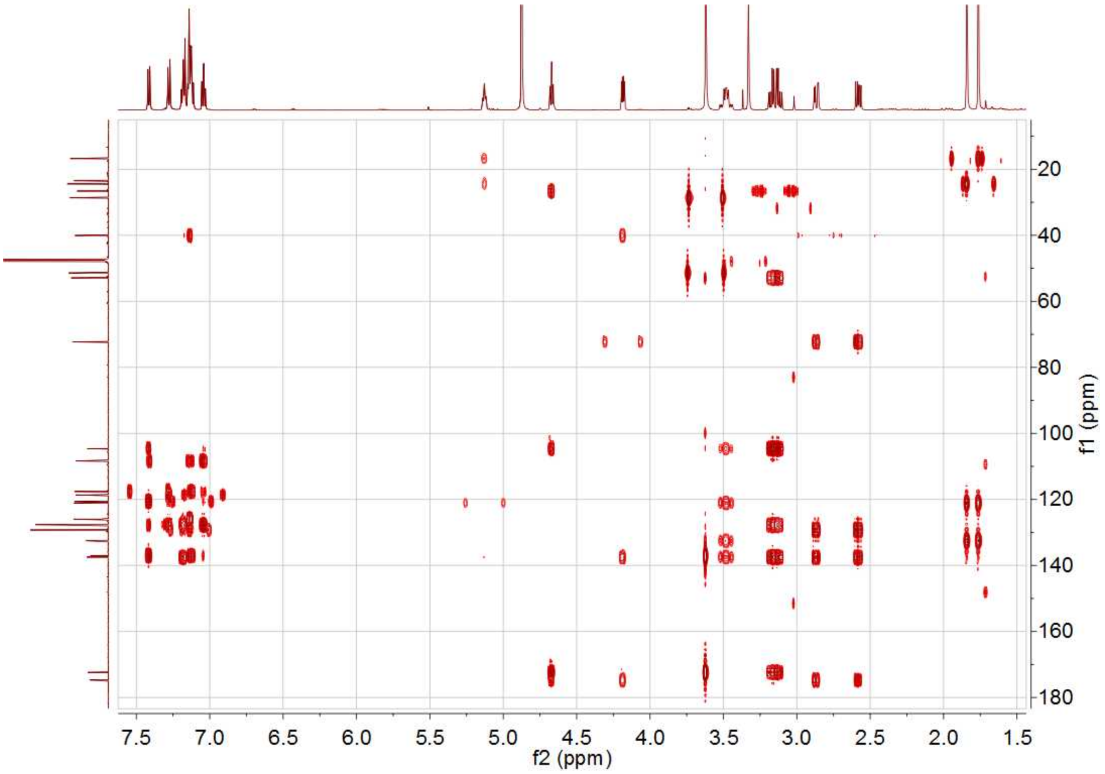

Figure S28. HMBC (600 and 150 MHz, methanol-d_4) spectrum of **4**

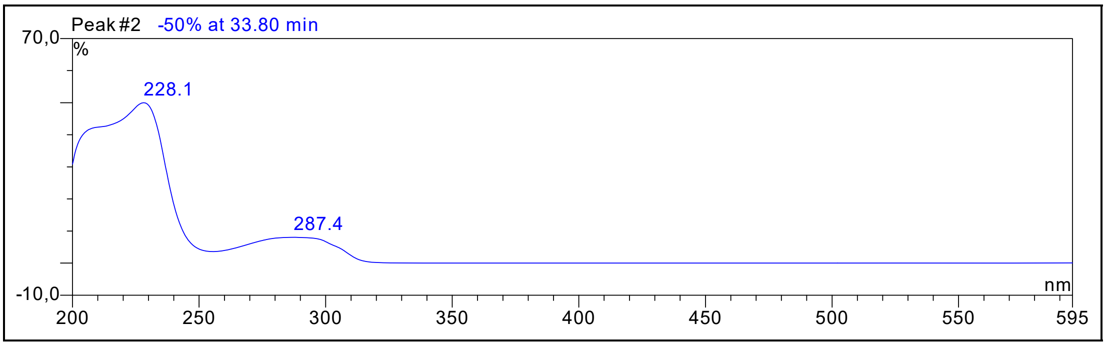

Figure S29. UV spectrum of **5**

Method	tune_low_new.m	Operator	Peter Tommes
Sample Name	Hao Wang AAN-22A--8-1 (CH3OH)	Instrument	maXis 288882.20213
Comment	oder 22A-28-1 ???<br>10 ul in 1 ml		

Acquisition Parameter

Source Type	ESI	Ion Polarity	Positive	Set Nebulizer	0.3 Bar
Focus	Not active	Set Capillary	4000 V	Set Dry Heater	180 °C
Scan Begin	50 m/z	Set End Plate Offset	-500 V	Set Dry Gas	4.0 l/min
Scan End	1500 m/z	Set Collision Cell RF	600.0 Vpp	Set Divert Valve	Source

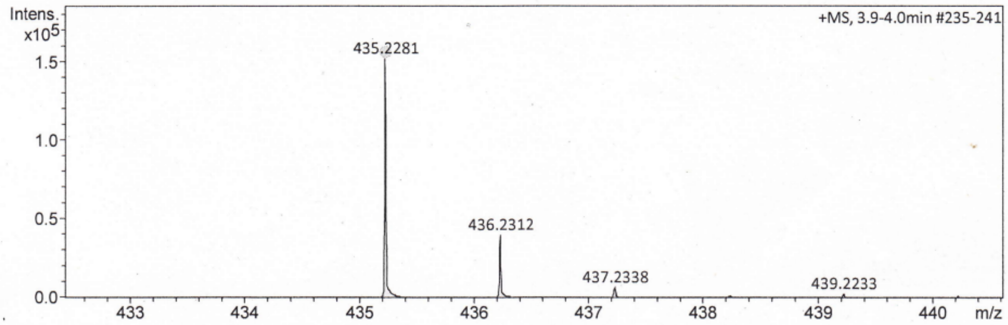

Meas. m/z	#	Ion Formula	m/z	err [ppm]	mSigma	# mSigma	Score	rdb	e⁻ Conf	N-Rule
435.2281	1	C26H31N2O4	435.2278	-0.7	18.9	1	100.00	12.5	even	ok

Figure S30. HRESIMS of **5**

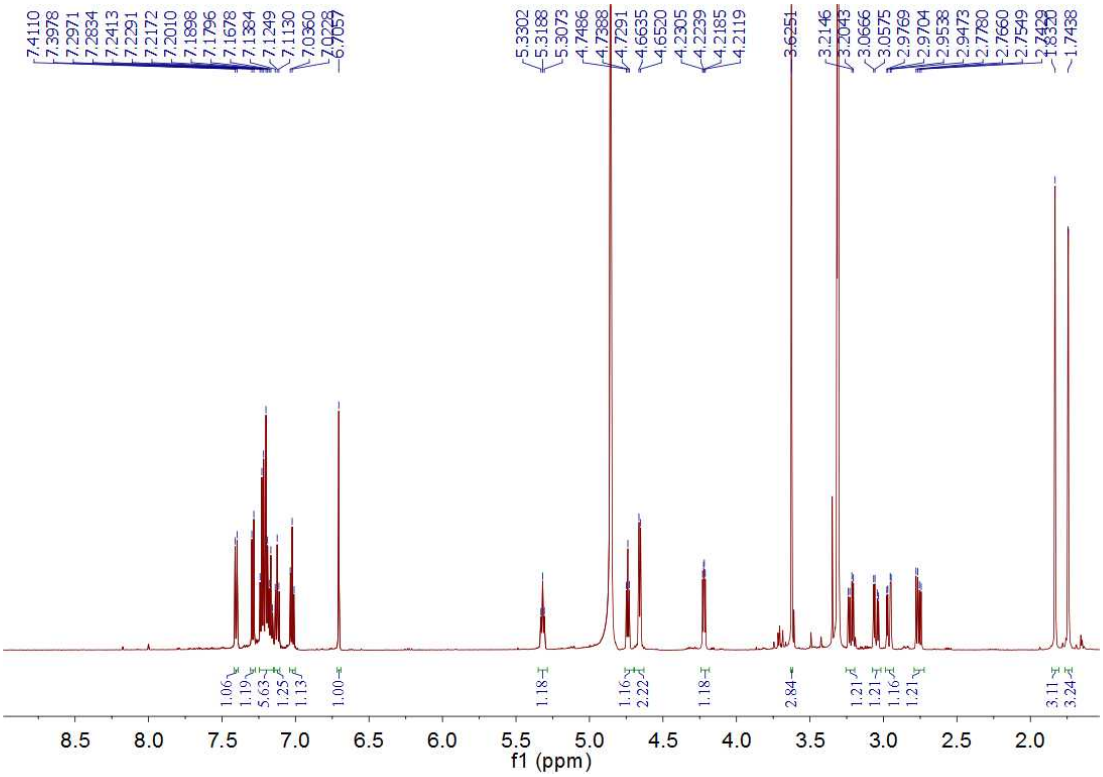

Figure S31. ^{1}H NMR (600 MHz, methanol-d_4) spectrum of **5**

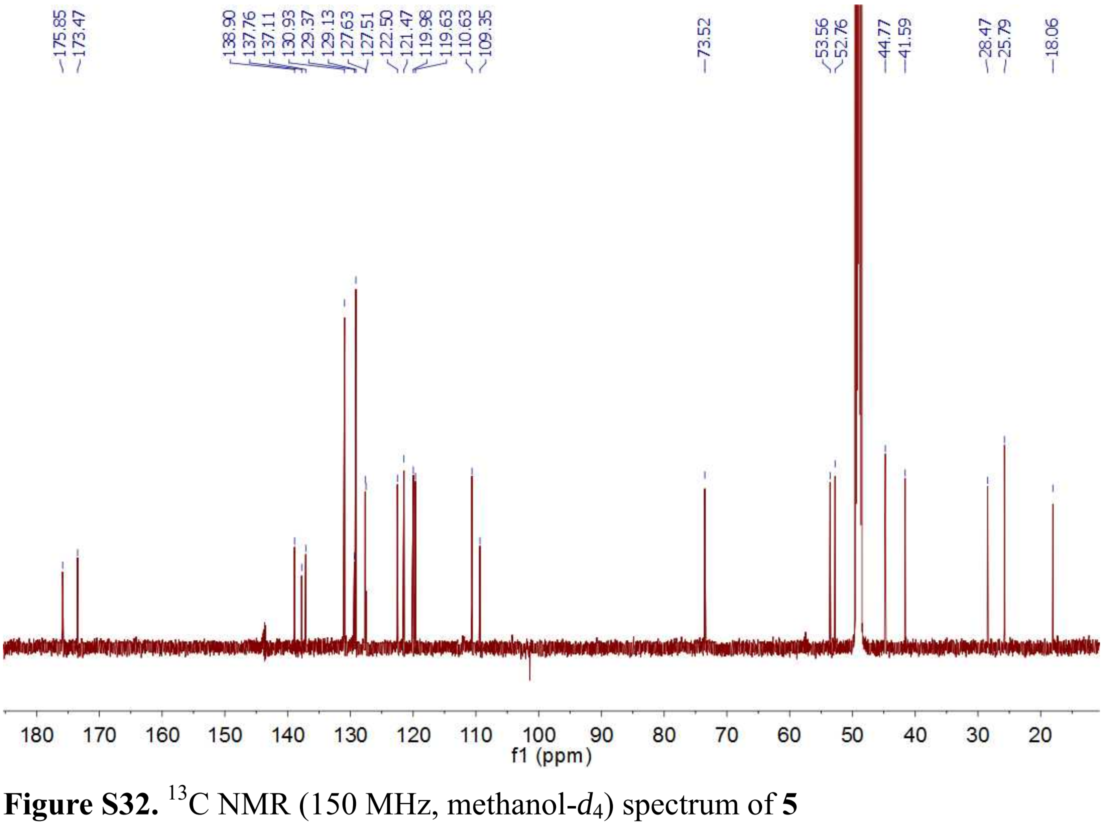

Figure S32. ^{13}C NMR (150 MHz, methanol-d_4) spectrum of **5**

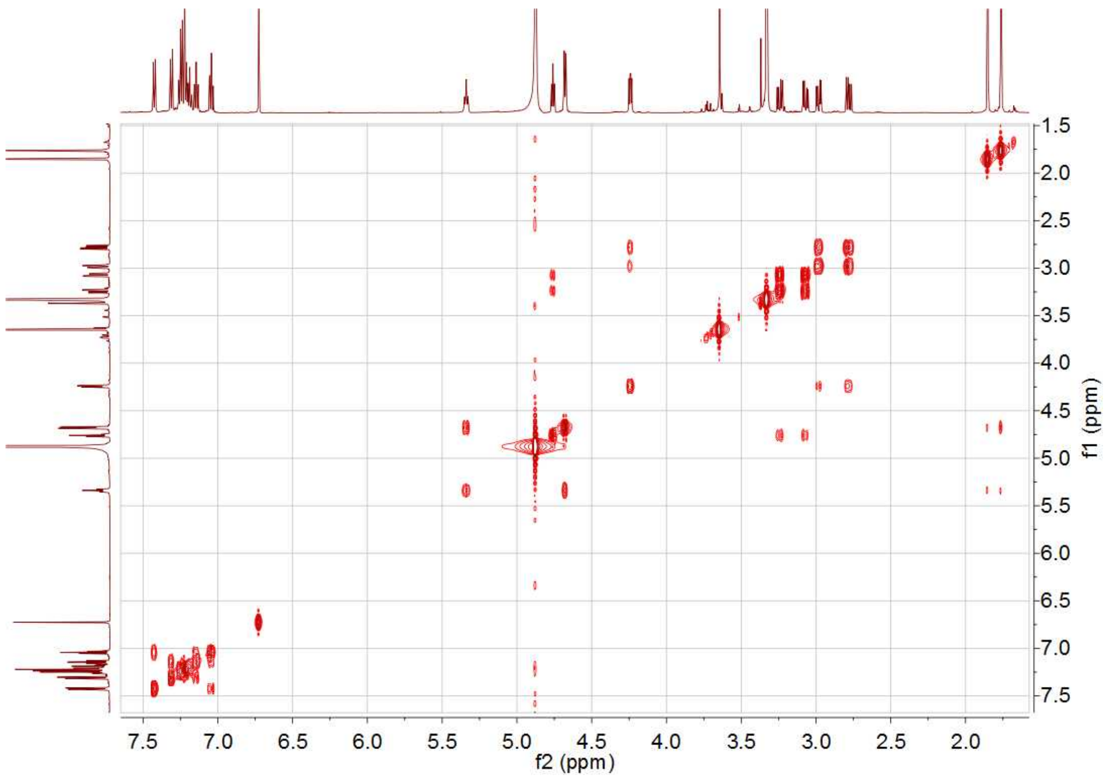

Figure S33. ^{1}H-^{1}H COSY (600 MHz, methanol-d_4) spectrum of **5**

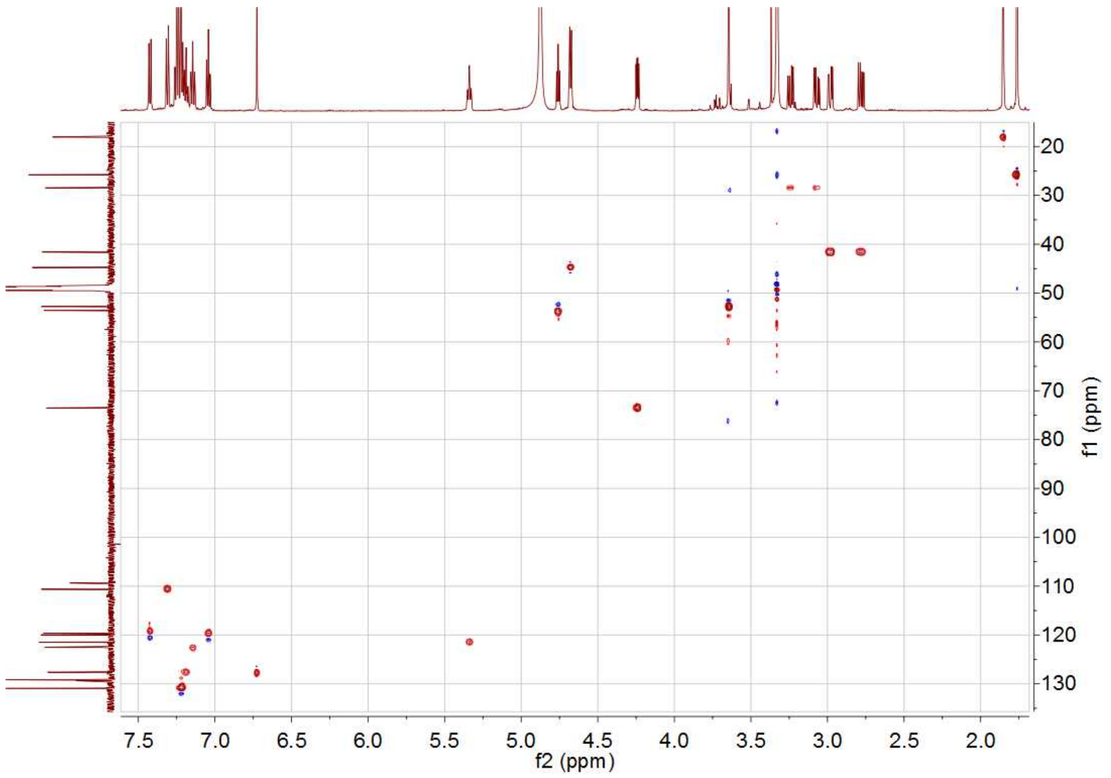

Figure S34. HSQC (600 and 150 MHz, methanol-d_4) spectrum of **5**

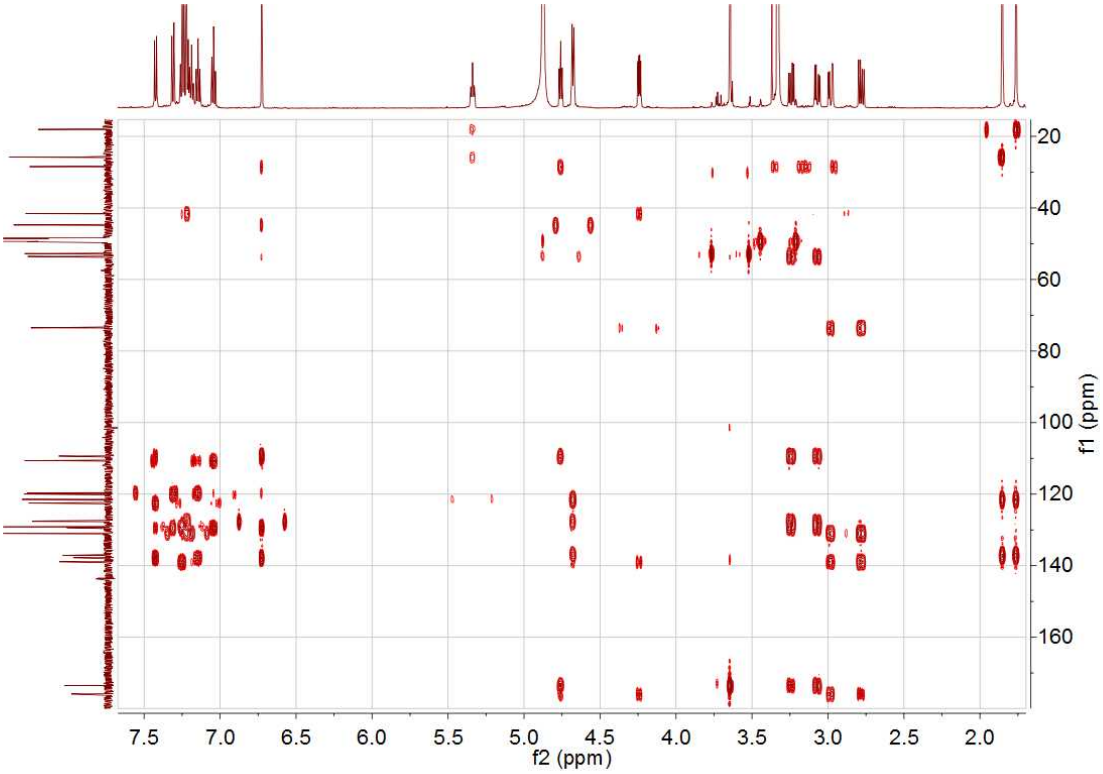

Figure S35. HMBC (600 and 150 MHz, methanol-d_4) spectrum of **5**

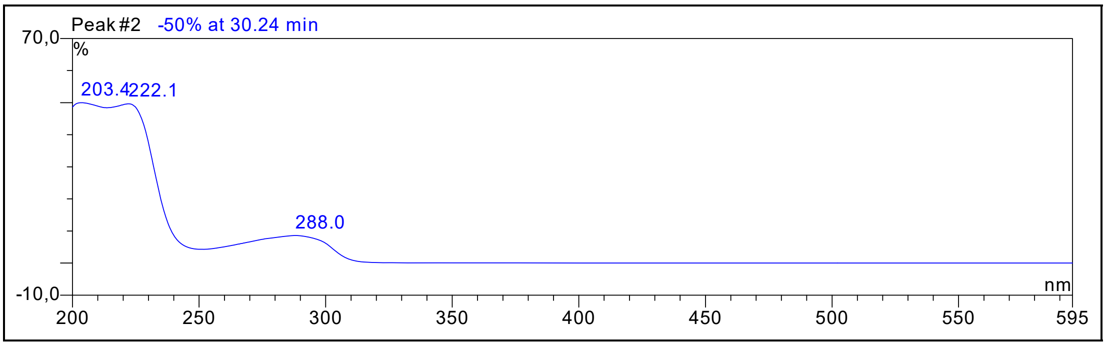

Figure S36. UV spectrum of **6**

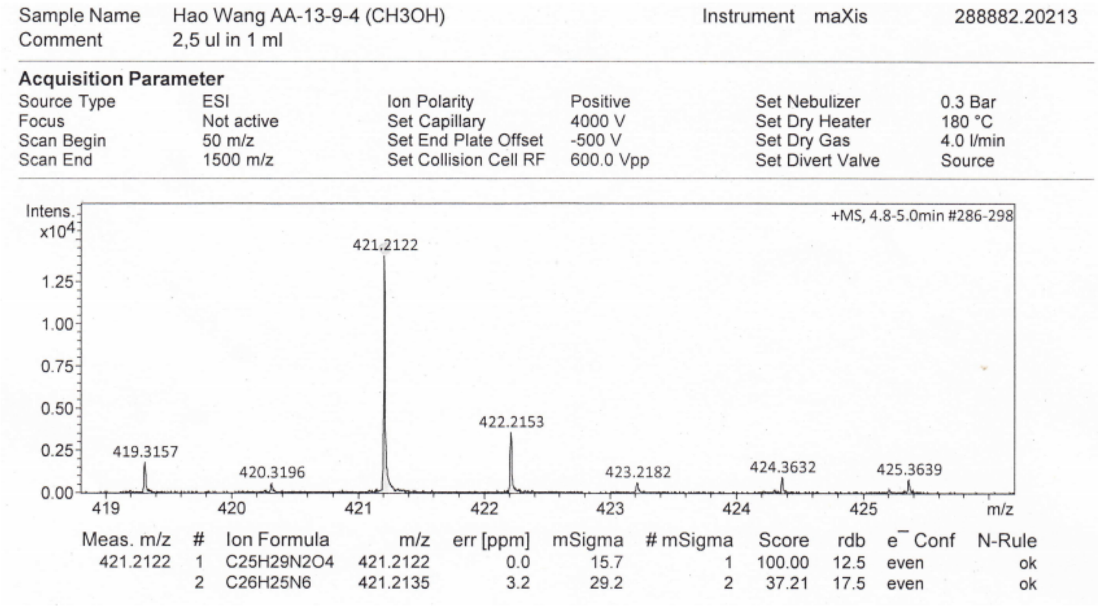

Figure S37. HRESIMS of **6**

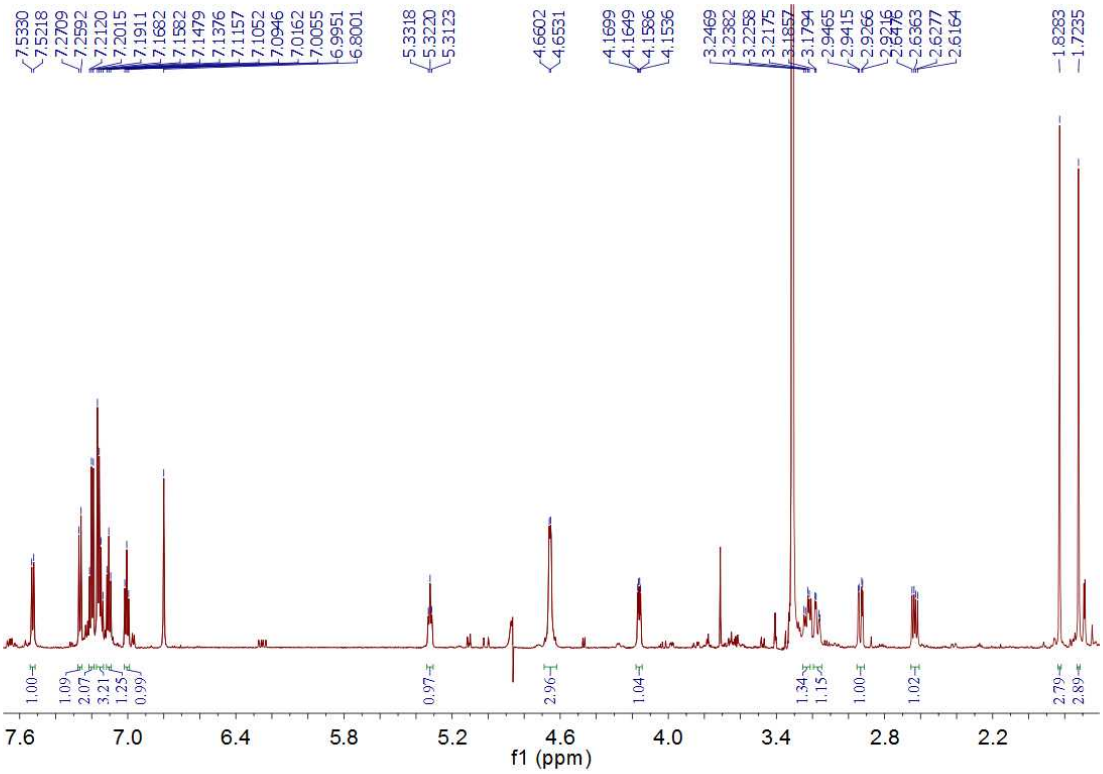

Figure S38. ^{1}H NMR (700 MHz, methanol-d_4) spectrum of **6**

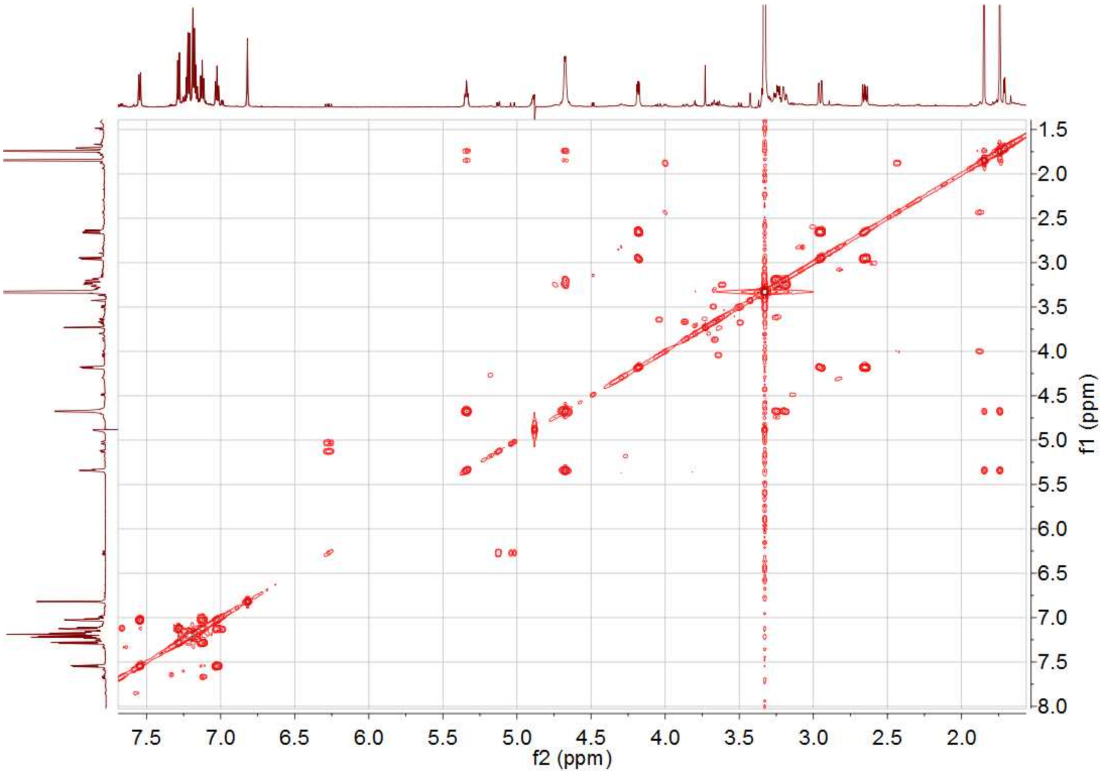

Figure S39. ^{1}H-^{1}H COSY (700 MHz, methanol-d_4) spectrum of **6**

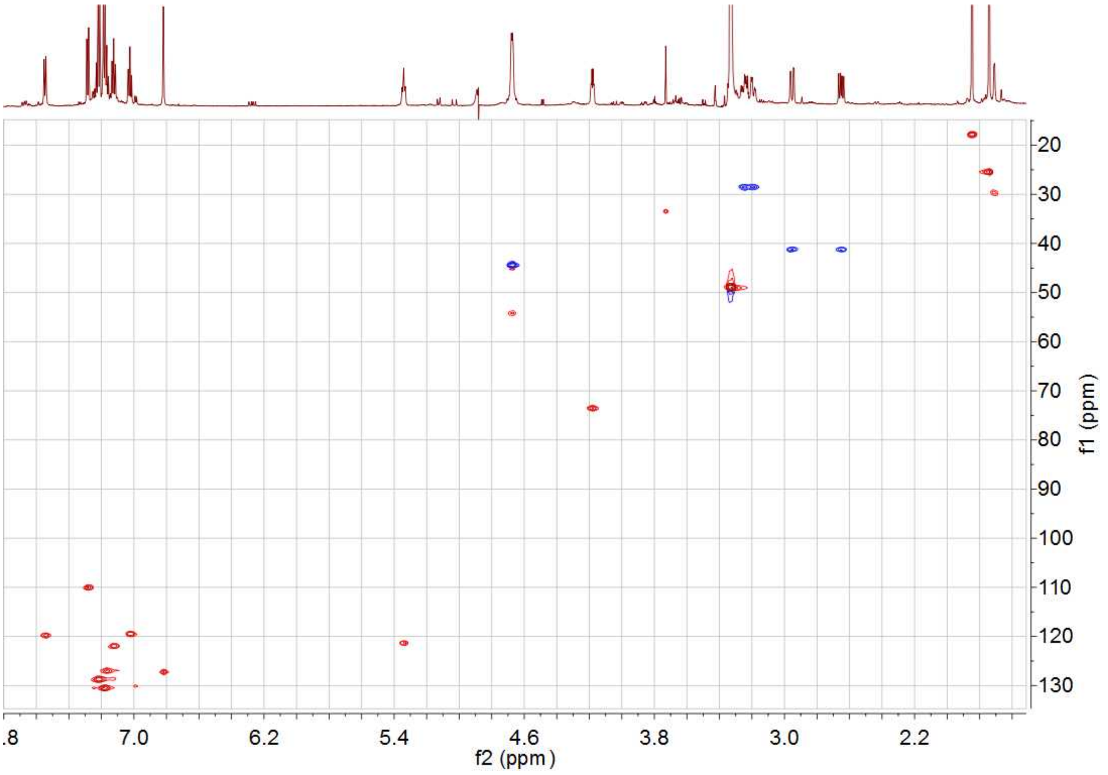

Figure S40. HSQC (700 and 175 MHz, methanol-d_4) spectrum of **6**

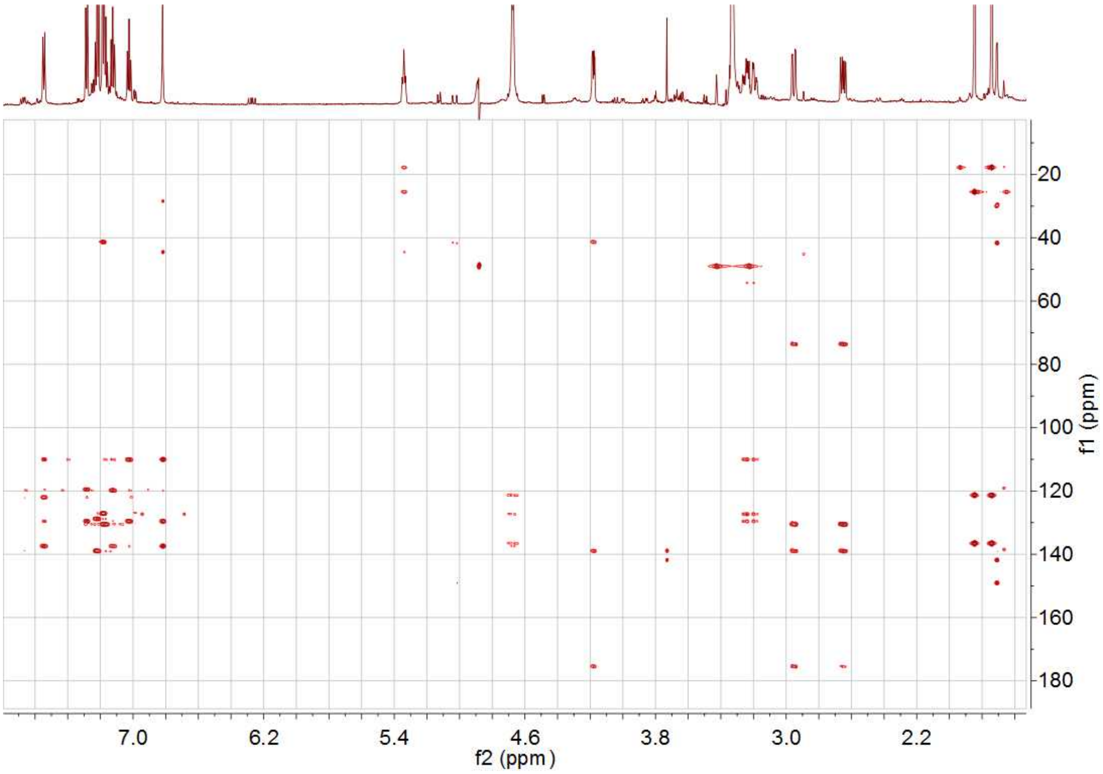

Figure S41. HMBC (700 and 175 MHz, methanol-d_4) spectrum of **6**

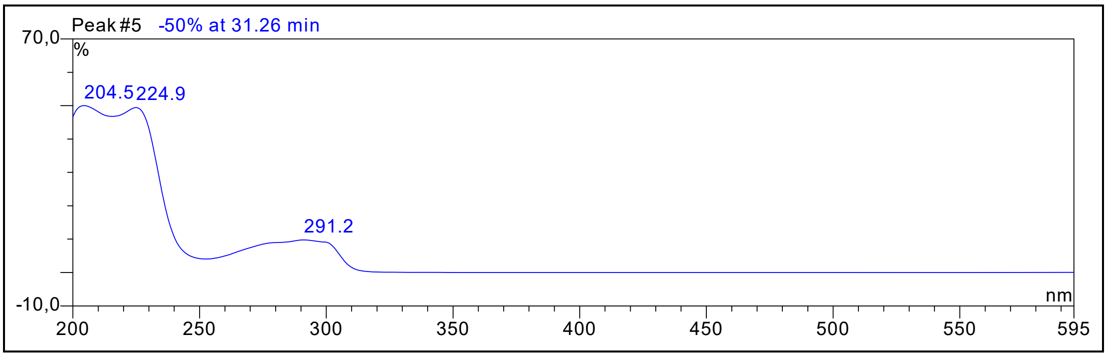

Figure S42. UV spectrum of **7**

Method	tune_low_new.m	Operator	Peter Tommes
Sample Name	Hao Wang AAN-22A-8-2 (CH3OH)	Instrument	maXis 288882.20213
Comment	10 ul in 1 ml		

Acquisition Parameter

Source Type	ESI	Ion Polarity	Positive	Set Nebulizer	0.3 Bar
Focus	Not active	Set Capillary	4000 V	Set Dry Heater	180 °C
Scan Begin	50 m/z	Set End Plate Offset	-500 V	Set Dry Gas	4.0 l/min
Scan End	1500 m/z	Set Collision Cell RF	600.0 Vpp	Set Divert Valve	Source

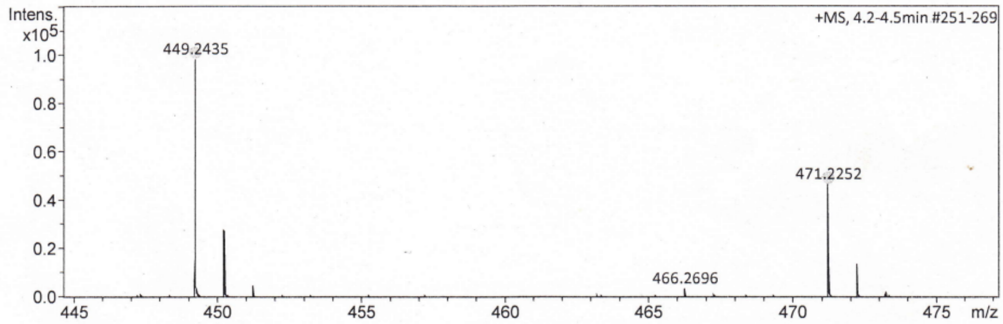

Meas. m/z	#	Ion Formula	m/z	err [ppm]	mSigma	# mSigma	Score	rdb	e⁻ Conf	N-Rule
449.2435	1	C27H33N2O4	449.2435	0.1	15.6	1	100.00	12.5	even	ok
471.2252	1	C27H32N2NaO4	471.2254	0.5	4.5	1	100.00	12.5	even	ok

Figure S43. HRESIMS of **7**

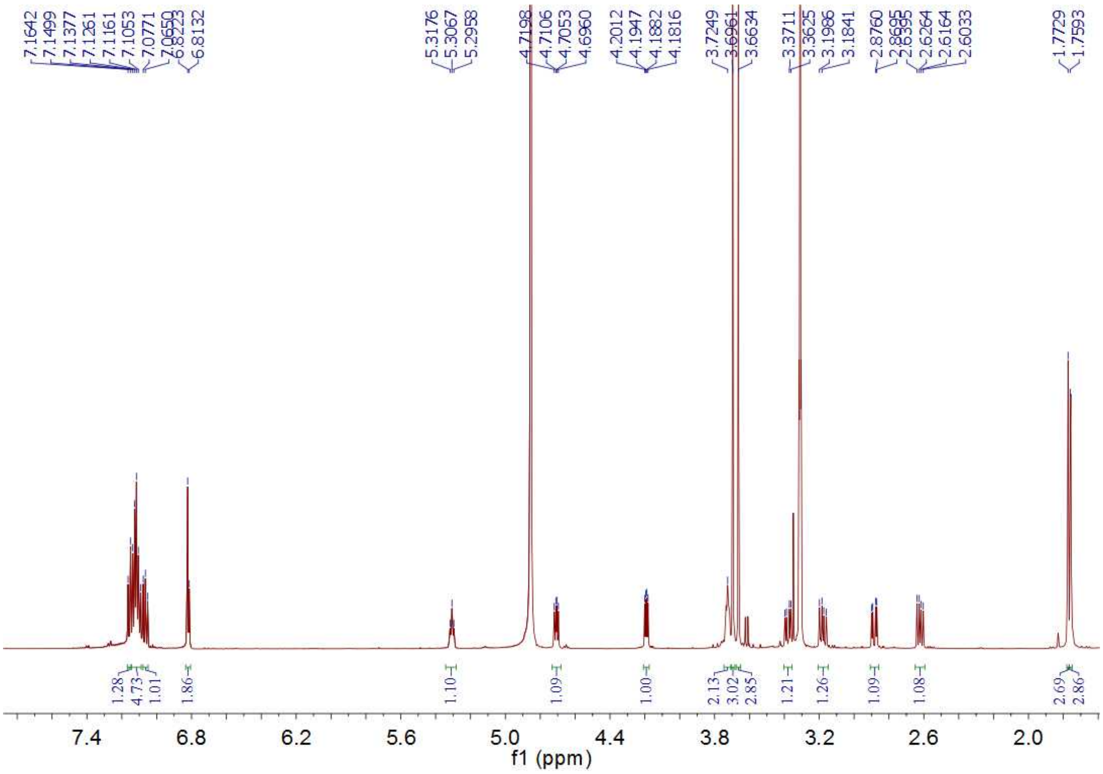

Figure S44. ^{1}H NMR (600 MHz, methanol-d_4) spectrum of **7**

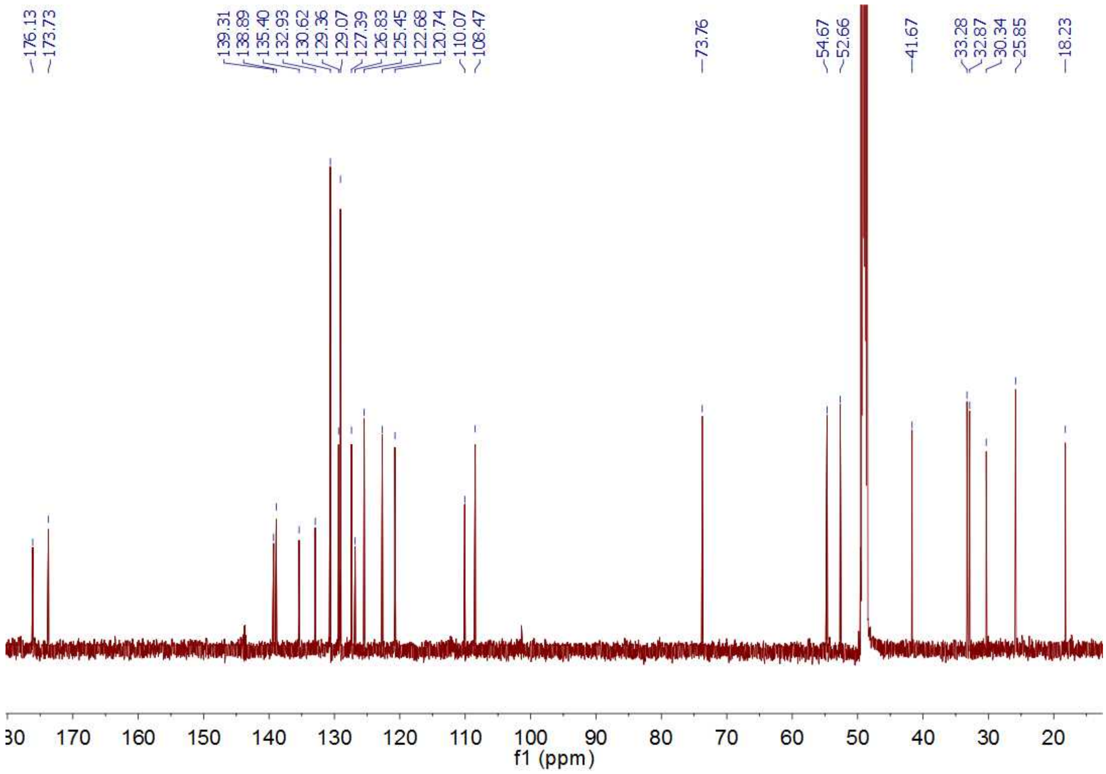

Figure S45. ^{13}C NMR (150 MHz, methanol-d_4) spectrum of **7**

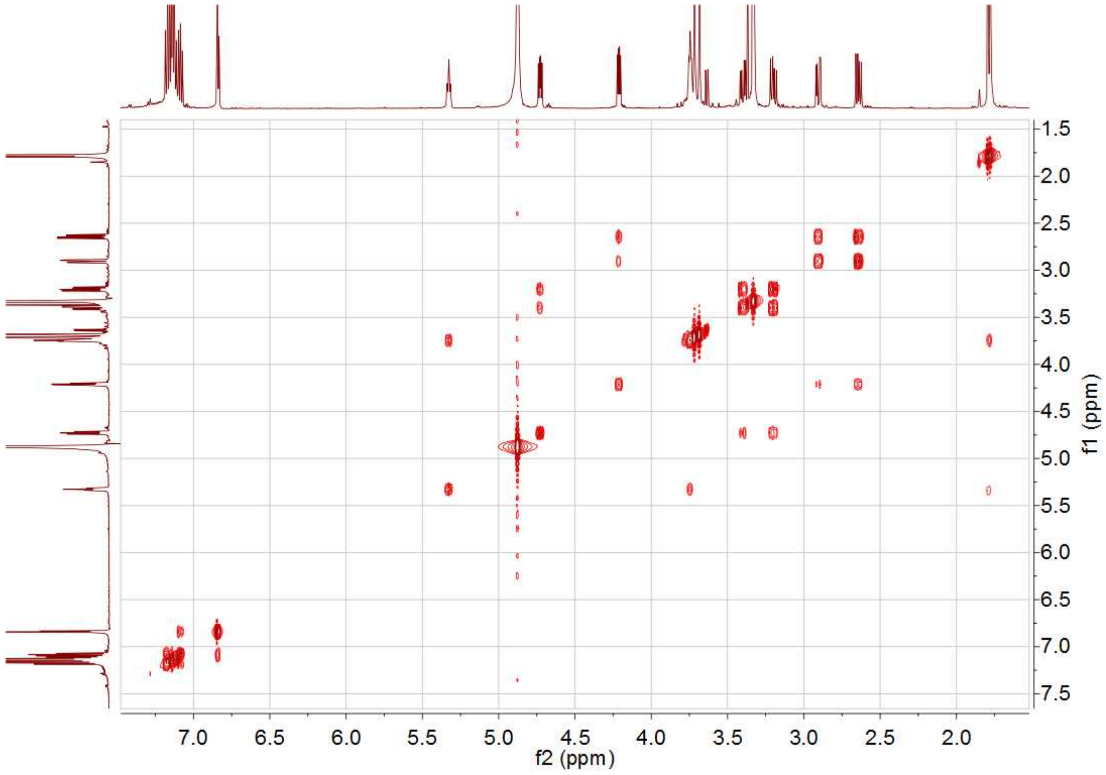

Figure S46. ^{1}H-^{1}H COSY (600 MHz, methanol-d_4) spectrum of **7**

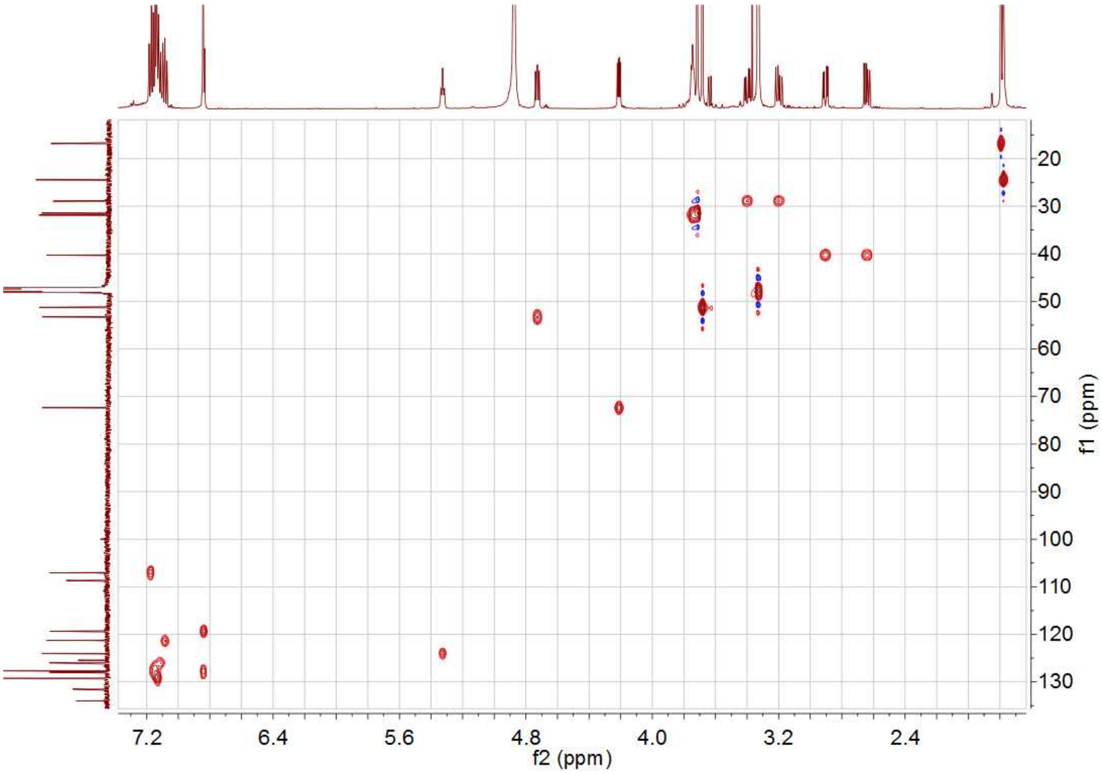

Figure S47. HSQC (600 and 150 MHz, methanol-d_4) spectrum of **7**

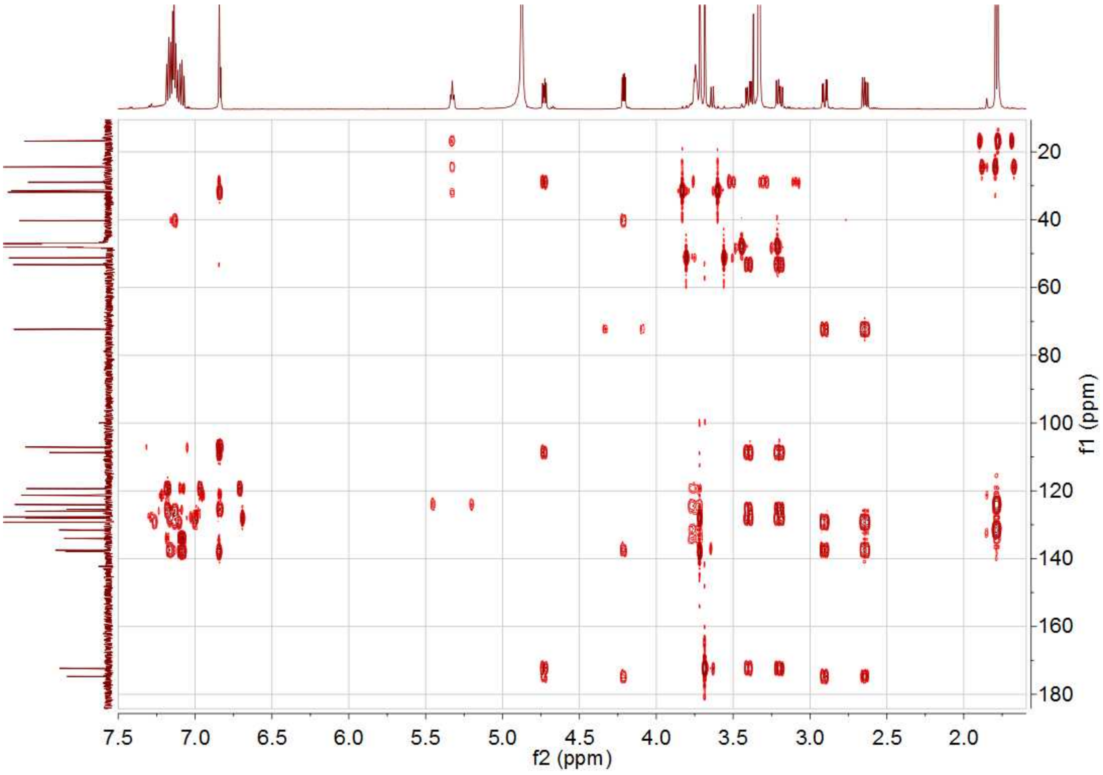

Figure S48. HMBC (600 and 150 MHz, methanol-d_4) spectrum of **7**

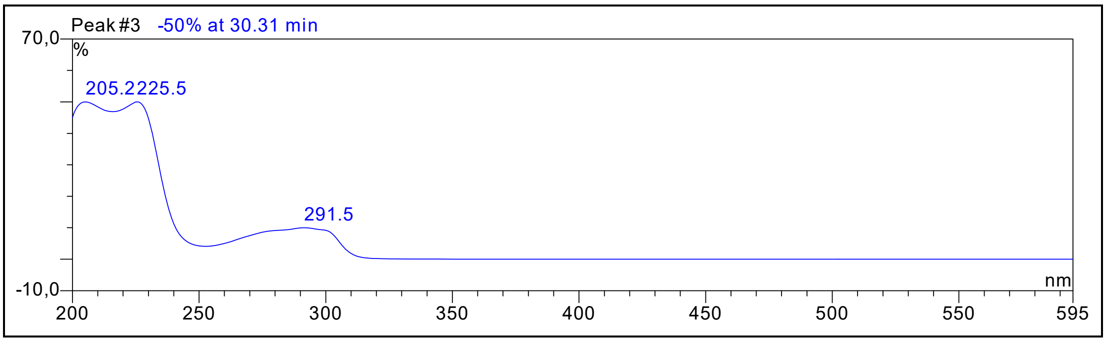

Figure S49. UV spectrum of **8**

Method	tune_low_new.m	Operator	Peter Tommes
Sample Name	Hao Wang AAN-22A-26-3 (CH3OH)	Instrument	maXis 288882.20213
Comment	10 ul in 1 ml		

Acquisition Parameter

Source Type	ESI	Ion Polarity	Positive	Set Nebulizer	0.3 Bar
Focus	Not active	Set Capillary	4000 V	Set Dry Heater	180 °C
Scan Begin	50 m/z	Set End Plate Offset	-500 V	Set Dry Gas	4.0 l/min
Scan End	1500 m/z	Set Collision Cell RF	600.0 Vpp	Set Divert Valve	Source

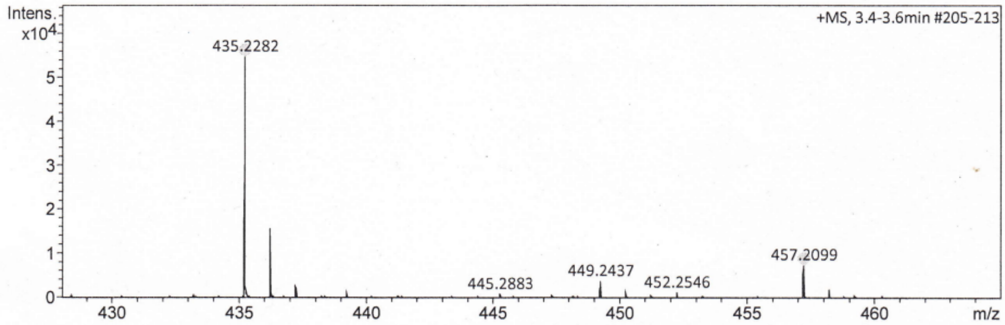

Meas. m/z	#	Ion Formula	m/z	err [ppm]	mSigma	# mSigma	Score	rdb	e⁻ Conf	N-Rule
435.2282	1	C26H31N2O4	435.2278	-0.8	5.5	1	100.00	12.5	even	ok
457.2099	1	C26H30N2NaO4	457.2098	-0.3	11.3	1	100.00	12.5	even	ok

Figure S50. HRESIMS of **8**

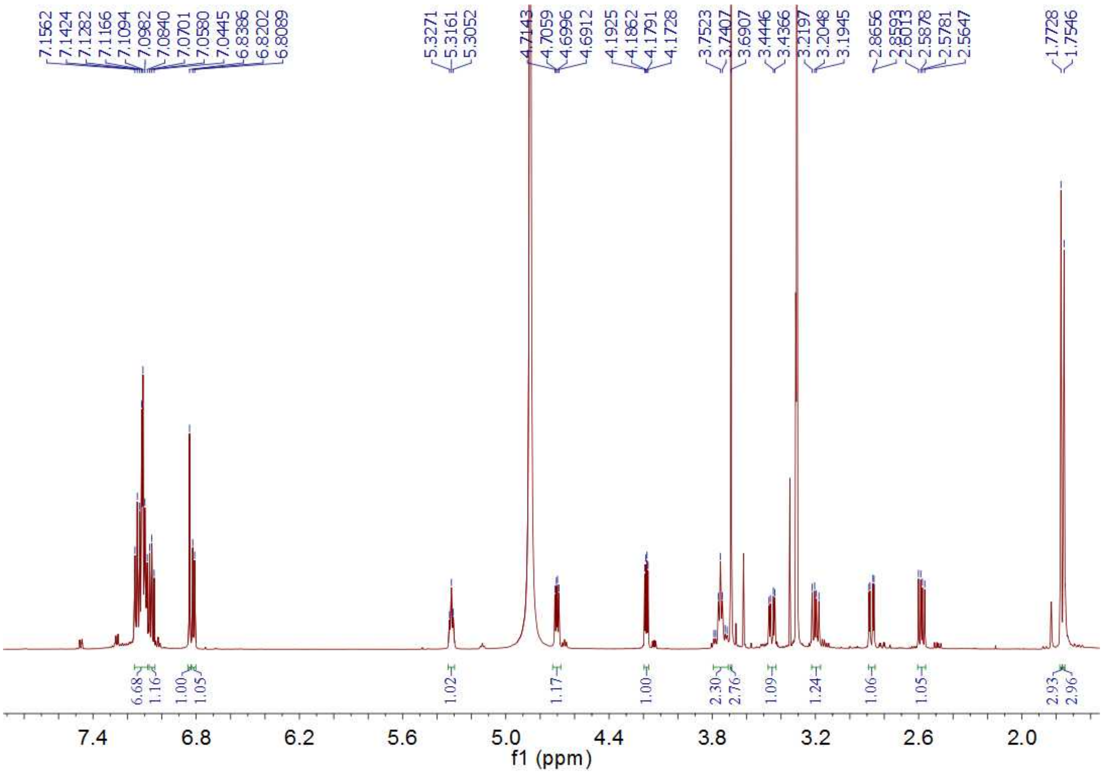

Figure S51. ^{1}H NMR (600 MHz, methanol-d_4) spectrum of **8**

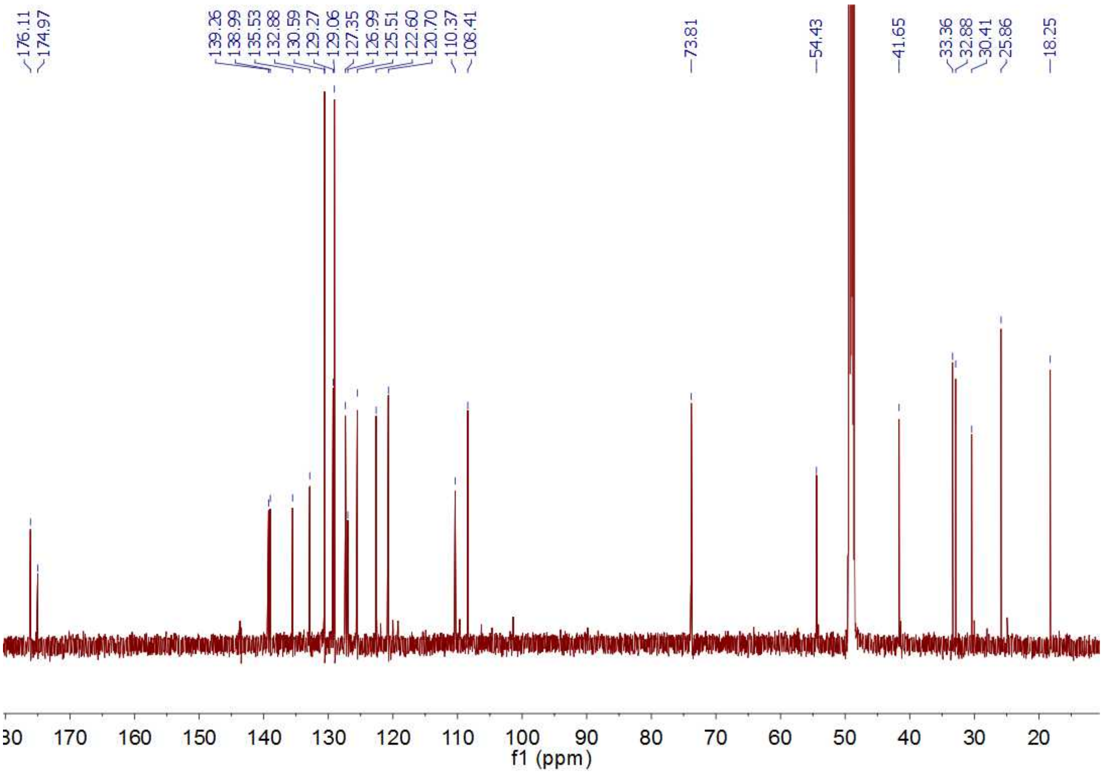

Figure S52. ^{13}C NMR (150 MHz, methanol-d_4) spectrum of **8**

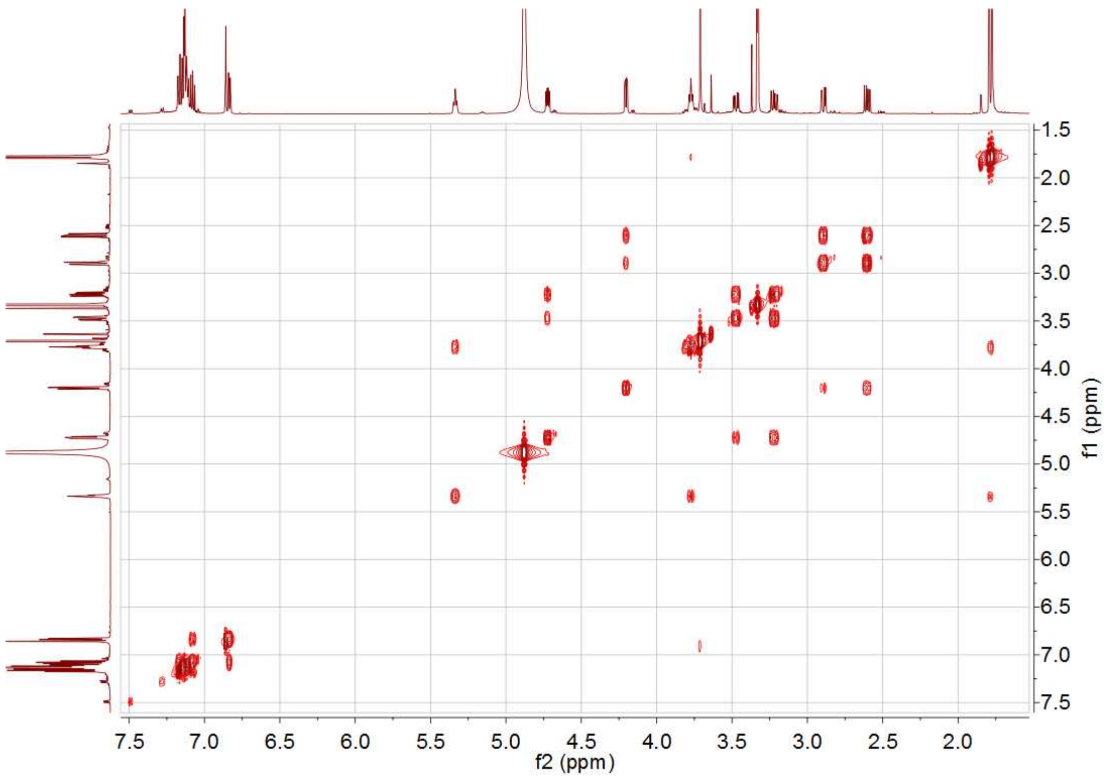

Figure S53. ^1H-^1H COSY (600 MHz, methanol-d_4) spectrum of **8**

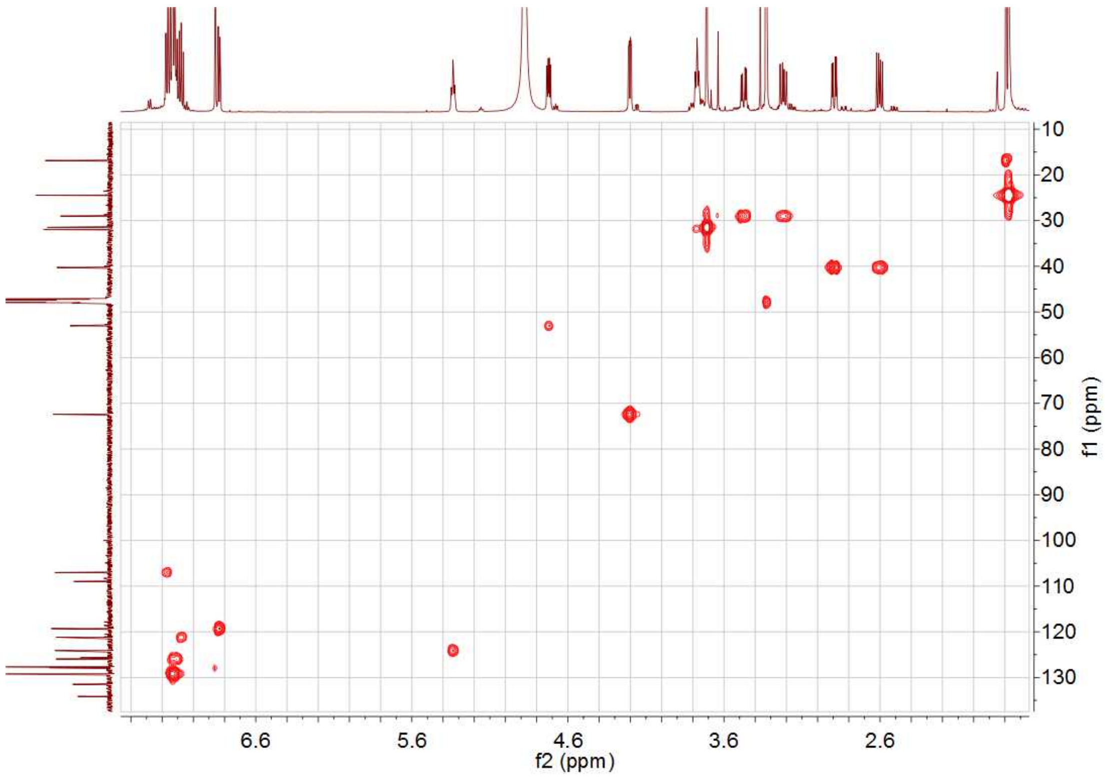

Figure S54. HSQC (600 and 150 MHz, methanol-d_4) spectrum of **8**

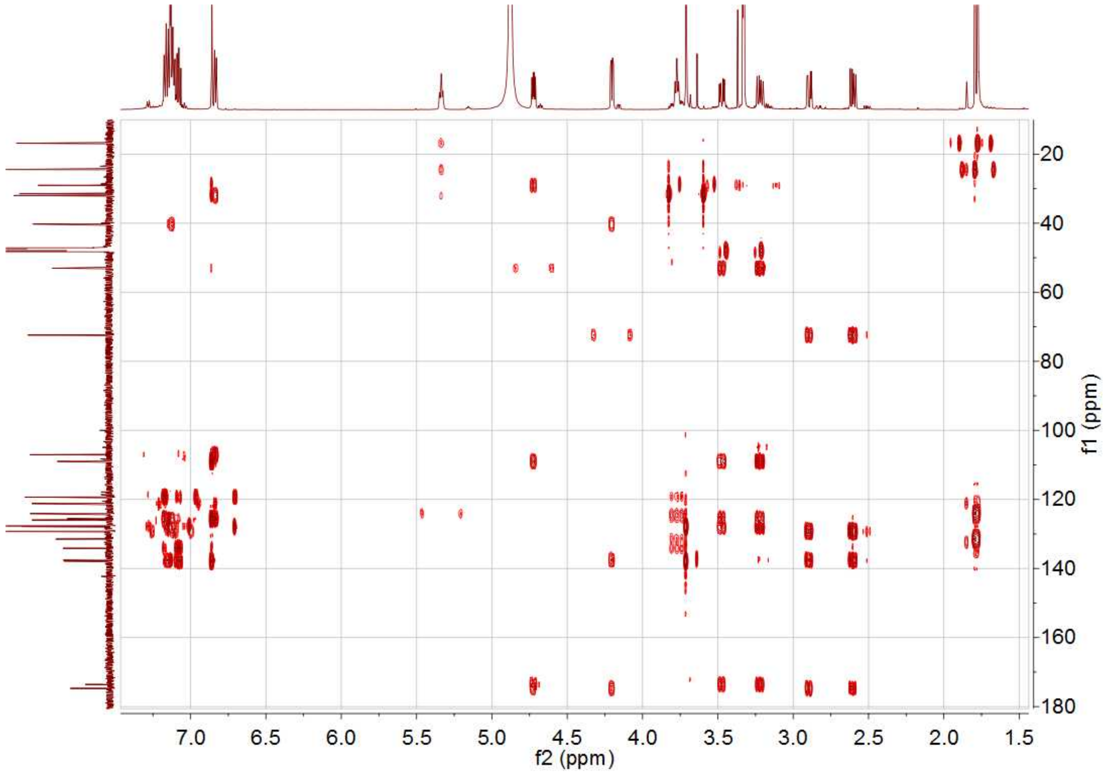

Figure S55. HMBC (600 and 150 MHz, methanol-d_4) spectrum of **8**

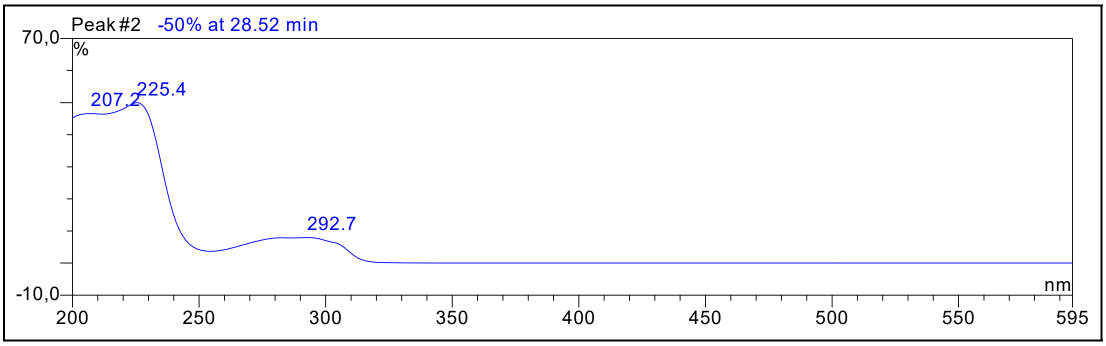

Figure S56. UV spectrum of **9**

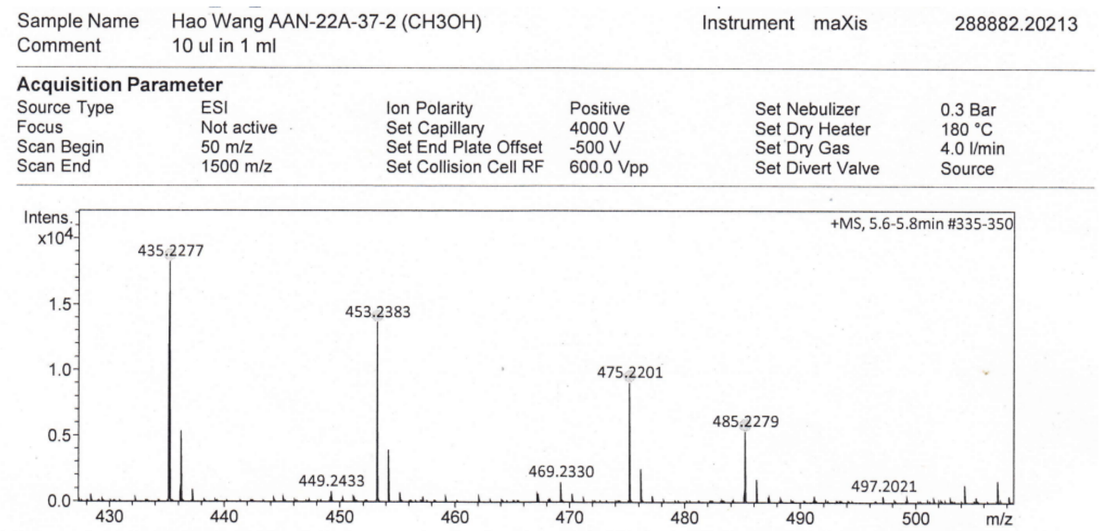

Meas. m/z	#	Ion Formula	m/z	err [ppm]	mSigma	# mSigma	Score	rdb	e⁻ Conf	N-Rule
435.2277	1	C26H31N2O4	435.2278	0.3	1.9	1	100.00	12.5	even	ok
453.2383	1	C26H33N2O5	453.2384	0.2	3.5	1	100.00	11.5	even	ok
475.2201	1	C26H32N2NaO5	475.2203	0.5	8.3	1	100.00	11.5	even	ok
485.2279	1	C26H33N2O7	485.2282	0.7	11.9	1	100.00	11.5	even	ok
	2	C25H30N6NaO3	485.2272	-1.5	13.2	2	59.73	13.5	even	ok

Figure S57. HRESIMS of **9**

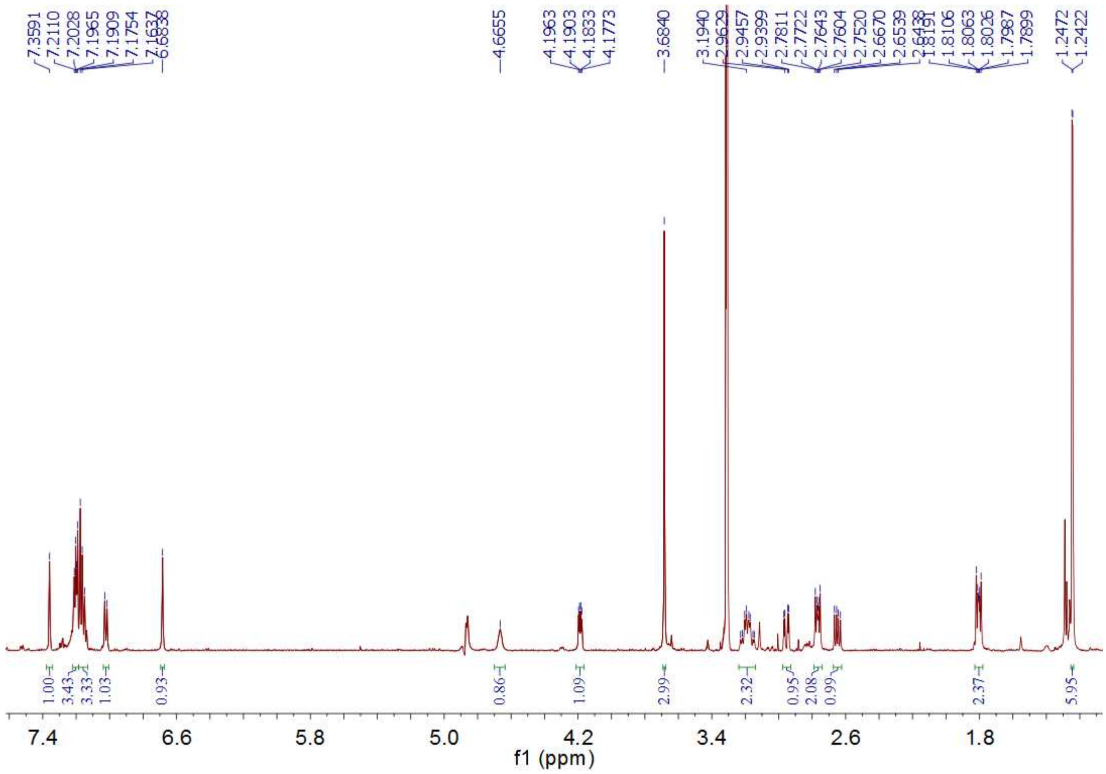

Figure S58. ^{1}H NMR (600 MHz, methanol-d_4) spectrum of **9**

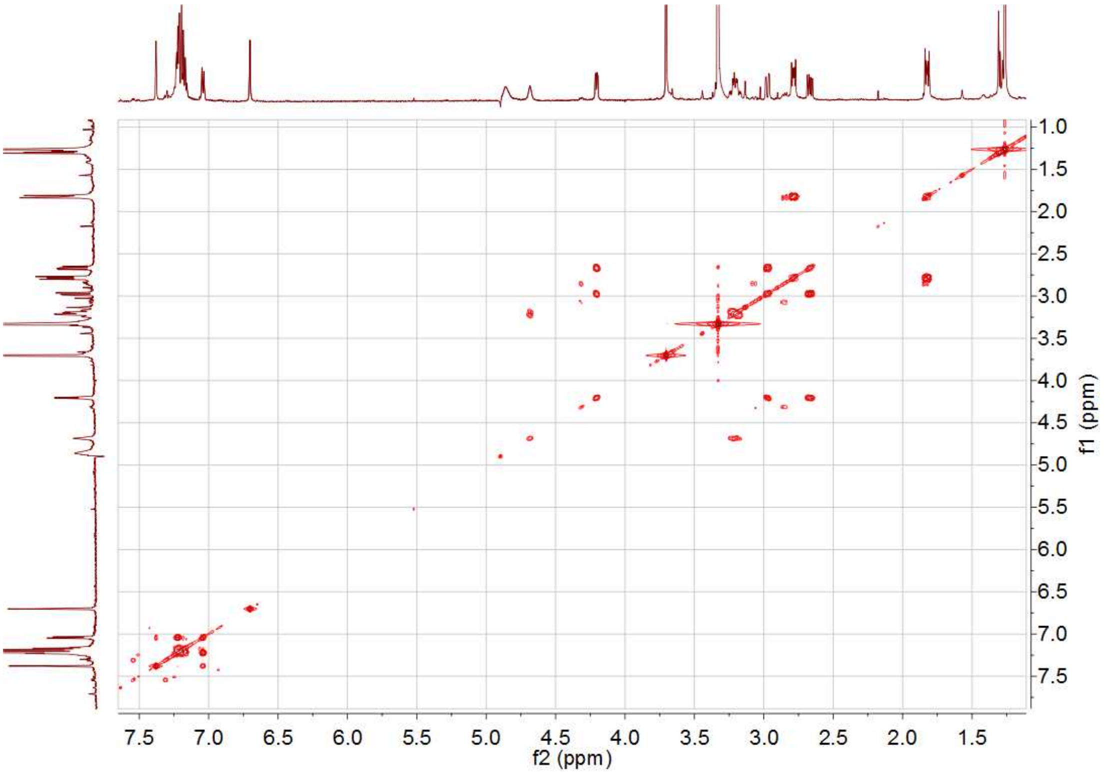

Figure S59. ^{1}H-^{1}H COSY (600 MHz, methanol-d_4) spectrum of **9**

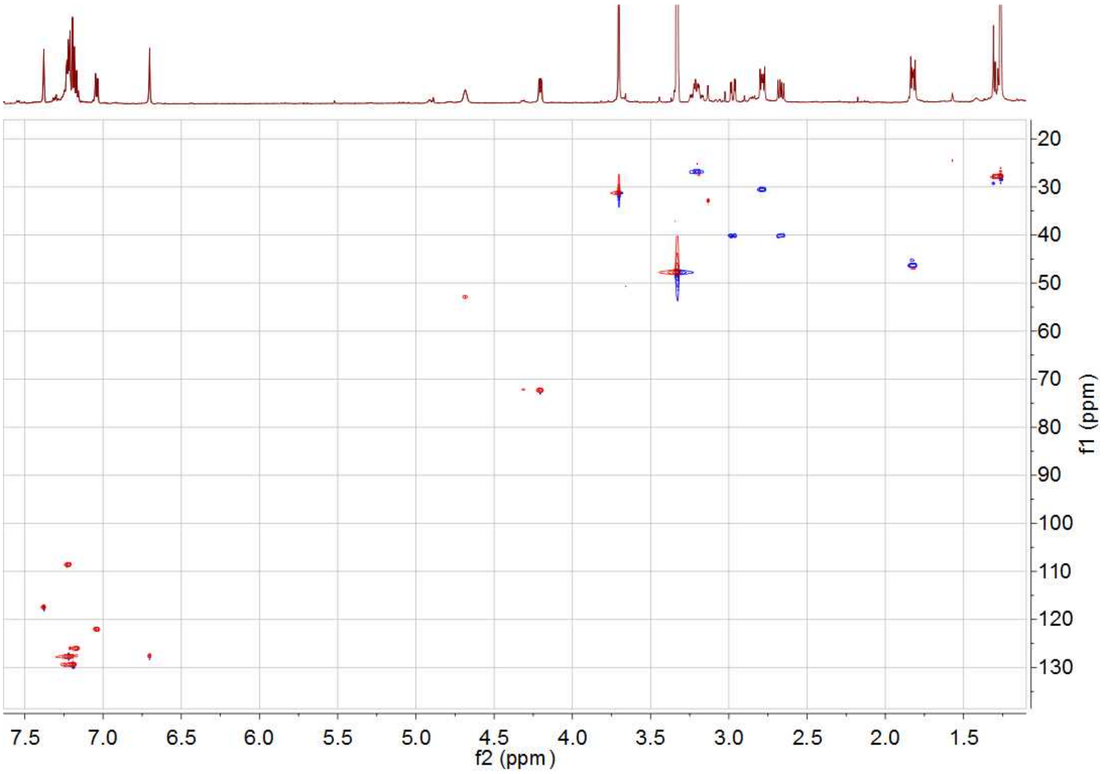

Figure S60. HSQC (600 and 150 MHz, methanol-d_4) spectrum of **9**

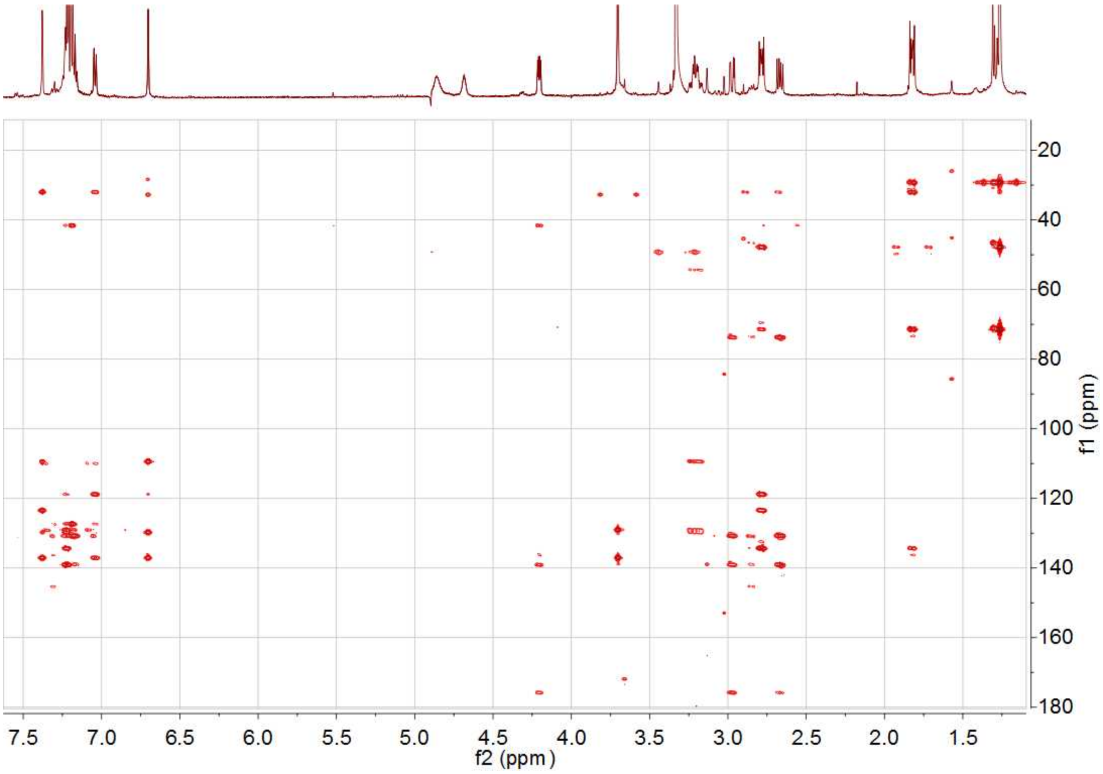

Figure S61. HMBC (600 and 150 MHz, methanol-d_4) spectrum of **9**

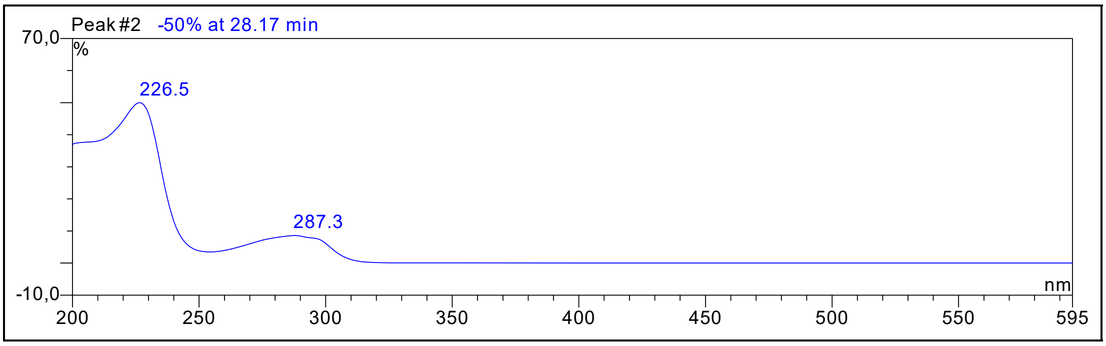

Figure S62. UV spectrum of **10**

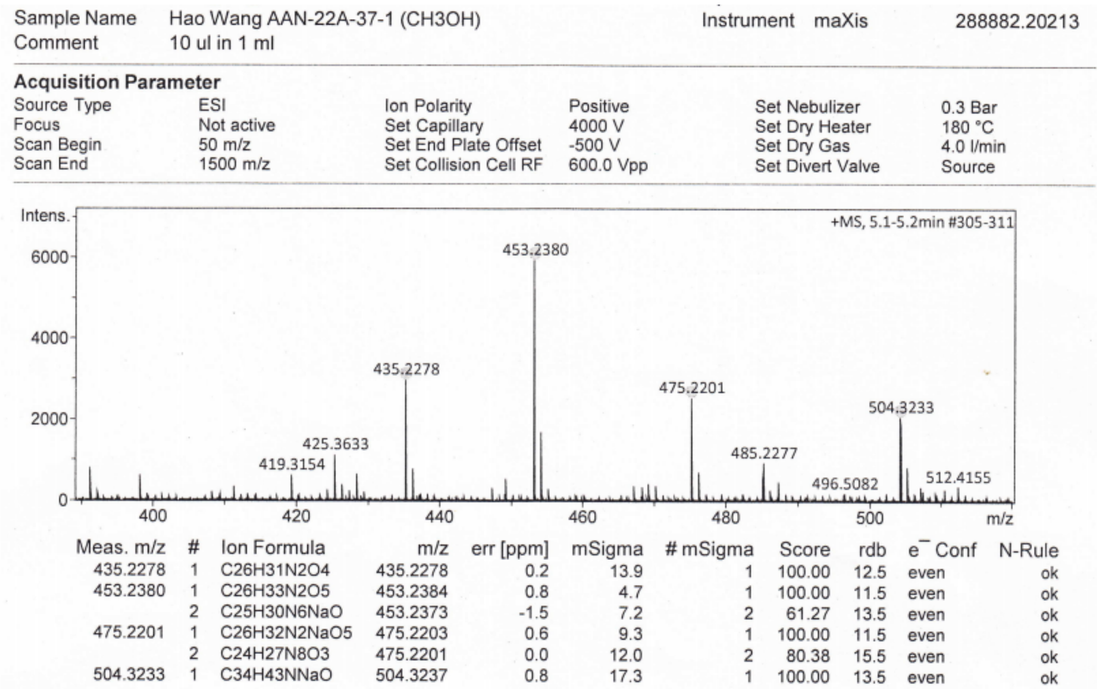

Meas. m/z	#	Ion Formula	m/z	err [ppm]	mSigma	# mSigma	Score	rdb	e¯ Conf	N-Rule
435.2278	1	C26H31N2O4	435.2278	0.2	13.9	1	100.00	12.5	even	ok
453.2380	1	C26H33N2O5	453.2384	0.8	4.7	1	100.00	11.5	even	ok
	2	C25H30N6NaO	453.2373	-1.5	7.2	2	61.27	13.5	even	ok
475.2201	1	C26H32N2NaO5	475.2203	0.6	9.3	1	100.00	11.5	even	ok
	2	C24H27N8O3	475.2201	0.0	12.0	2	80.38	15.5	even	ok
504.3233	1	C34H43NNaO	504.3237	0.8	17.3	1	100.00	13.5	even	ok

Figure S63. HRESIMS of **10**

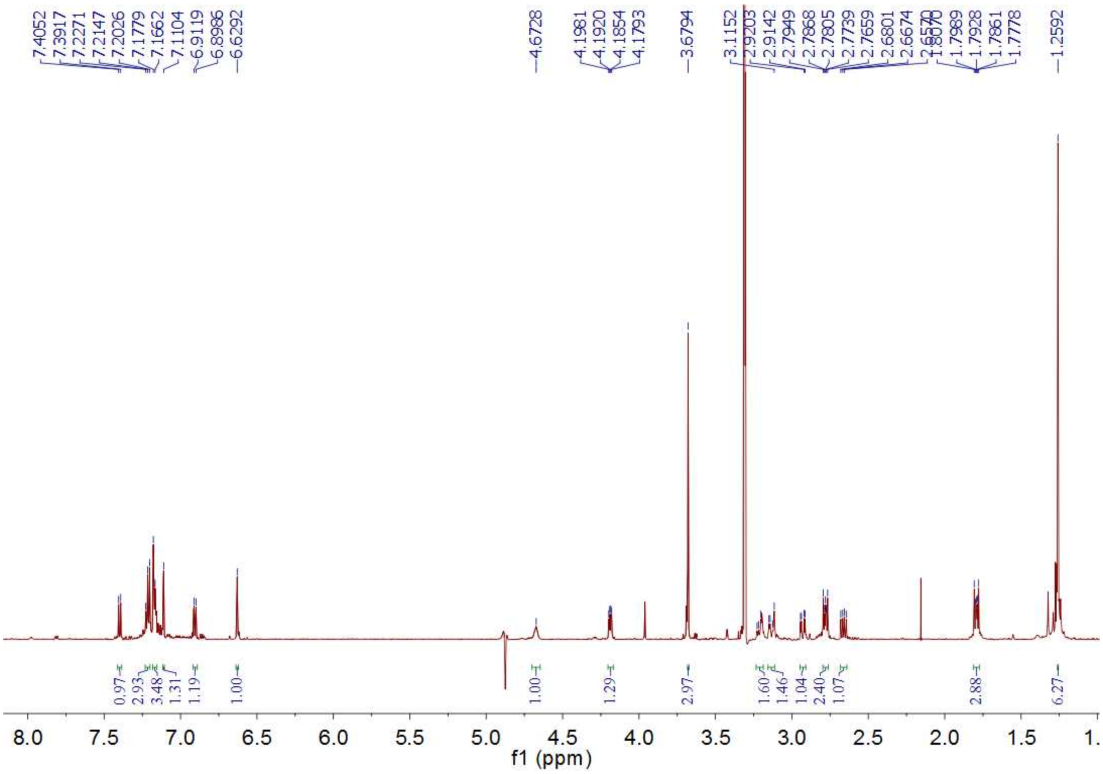

Figure S64. ^{1}H NMR (600 MHz, methanol-d_4) spectrum of **10**

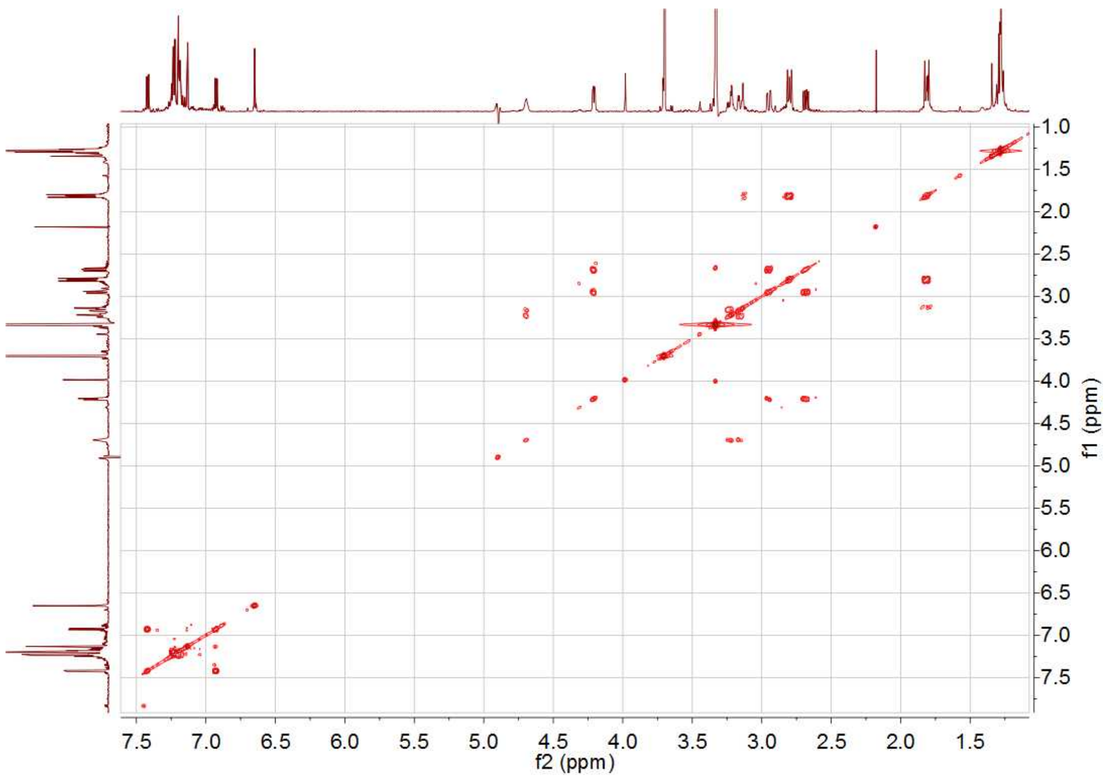

Figure S65. ^{1}H-^{1}H COSY (600 MHz, methanol-d_4) spectrum of **10**

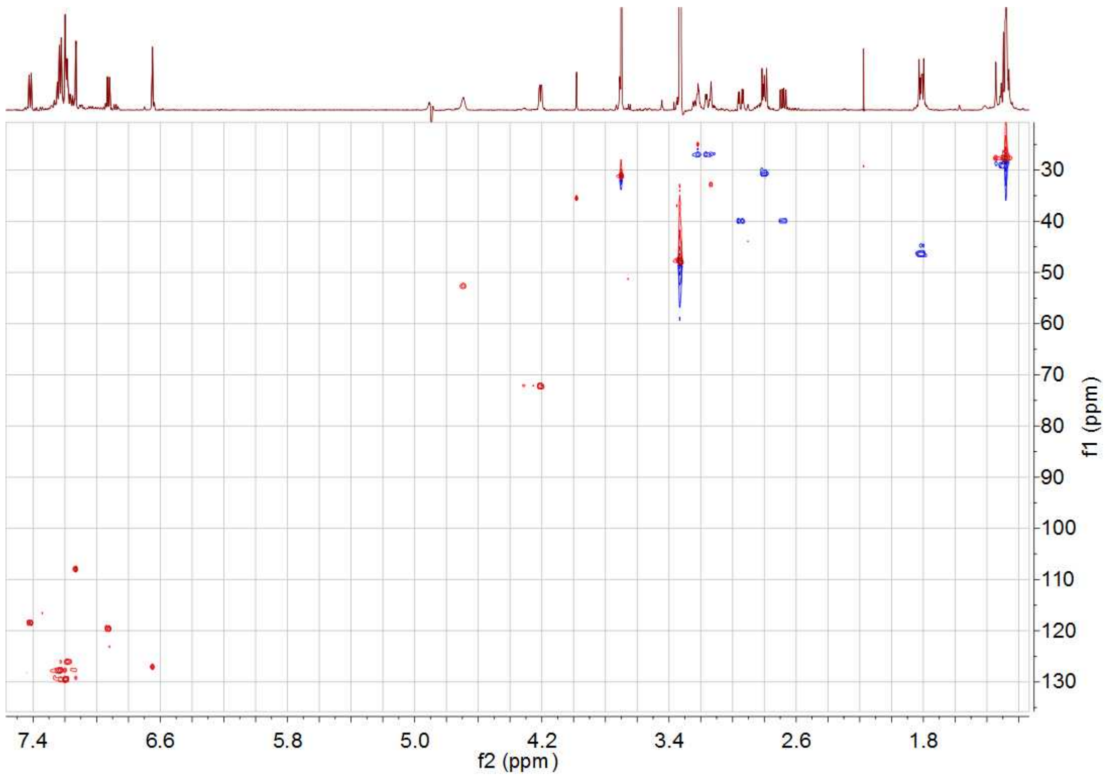

Figure S66. HSQC (600 and 150 MHz, methanol-d_4) spectrum of **10**

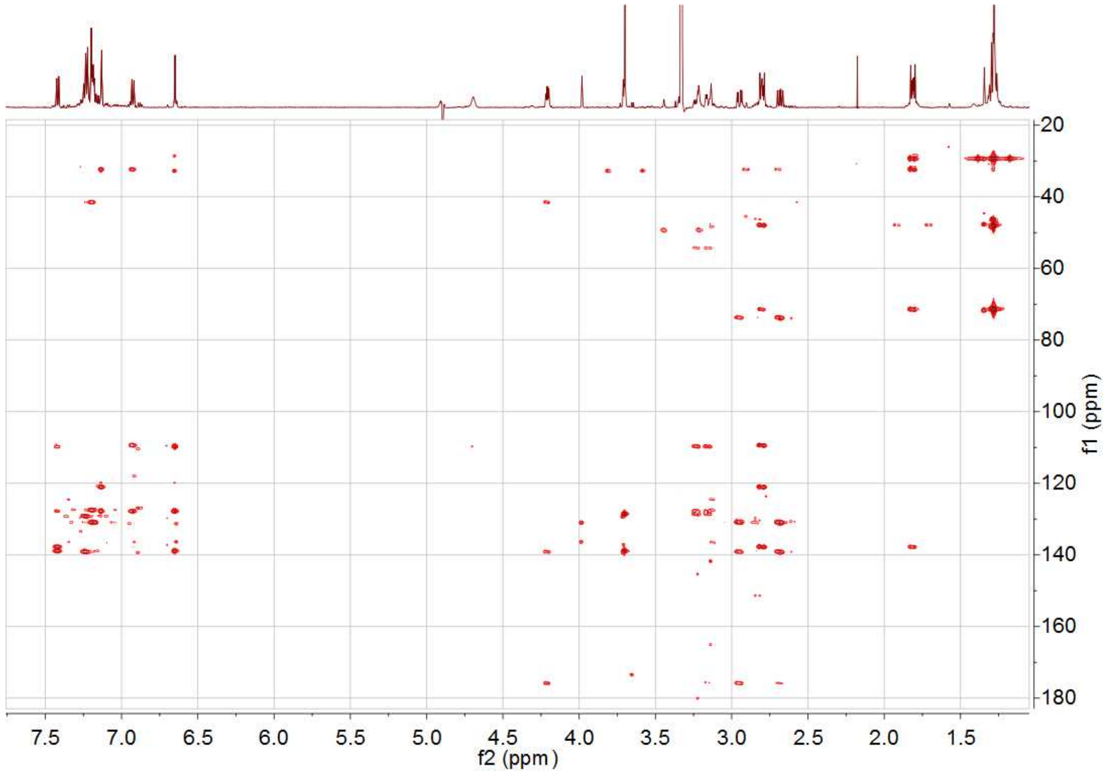

Figure S67. HMBC (600 and 150 MHz, methanol-d_4) spectrum of **10**

Results of X-ray analysis of compound 1

Due to very small crystal sizes and poor crystal quality of **1**, only a very low resolution was obtainable. Crystals of **1** did not diffract beyond $q = 44.9°$ (cf. desired 67.7°) for Cu-Kα radiation, resulting in only 1538 total (1484 observed with $I > 2s(I)$) reflections versus 263 parameters for anisotropic refinement. Therefore the cif does not meet the requirements for publication. The relevance of the following reported analysis of **1** should not be overestimated and interpreted carefully.

Crystal data

$C_{22}H_{24}N_2O_4$	$Z = 4$
$M_r = 380.43$	$F(000) = 808$
Orthorhombic, $P2_12_12_1$	$D_x = 1.305$ Mg m^{-3}
$a = 5.9832$ (4) Å	Cu *K*α radiation, λ = 1.54178 Å
$b = 11.8099$ (7) Å	μ = 0.73 mm^{-1}
$c = 27.4081$ (17) Å	$T = 140$ K
$V = 1936.7$ (2) Å^3	0.12 × 0.03 × 0.03 mm^3

Data collection

16751 measured reflections	$\theta_{max} = 44.9°$, $\theta_{min} = 3.2°$

1538 independent reflections	h = -5→5
1484 reflections with $I > 2\sigma(I)$	k = -10→10
R_{int} = 0.051	l = -25→24

Refinement

Refinement on F^2	Hydrogen site location: mixed
Least-squares matrix: full	H atoms treated by a mixture of independent and constrained refinement
$R[F^2 > 2\sigma(F^2)]$ = 0.022	$w = 1/[\sigma^2(F_o^2) + (0.0353P)^2]$<br>where $P = (F_o^2 + 2F_c^2)/3$
$wR(F^2)$ = 0.053	$(\Delta/\sigma)_{max}$ = 0.109
S = 1.12	$\Delta\rangle_{max}$ = 0.002 e Å^{-3}
1538 reflections	$\Delta\rangle_{min}$ = -0.001 e Å^{-3}
263 parameters	Absolute structure: Flack x determined using 561 quotients [(I+)-(I-)]/[(I+)+(I-)] (Parsons, Flack and Wagner, ActaCryst. B69 (2013) 249-259).
0 restraints	Absolute structure parameter: -0.14 (10)

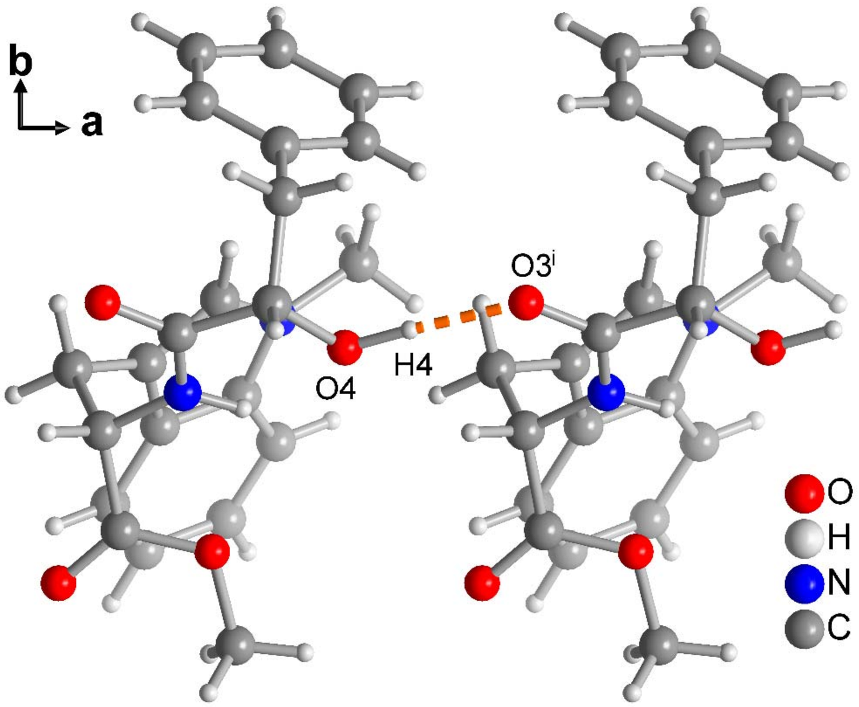

Figure S68. Section of the packing diagram of **1** showing the intermolecular H-bond (orange dashed line), which connects two symmetry equivalent molecules. Details for intermolecular H-bond:O4-H41.00 Å, H4···O3^{i}1.72 Å, O4···O3^{i}2.71 Å, O4-H4···O3^{i} 171 °, Symmetry code: (i) 1+x, y, z.

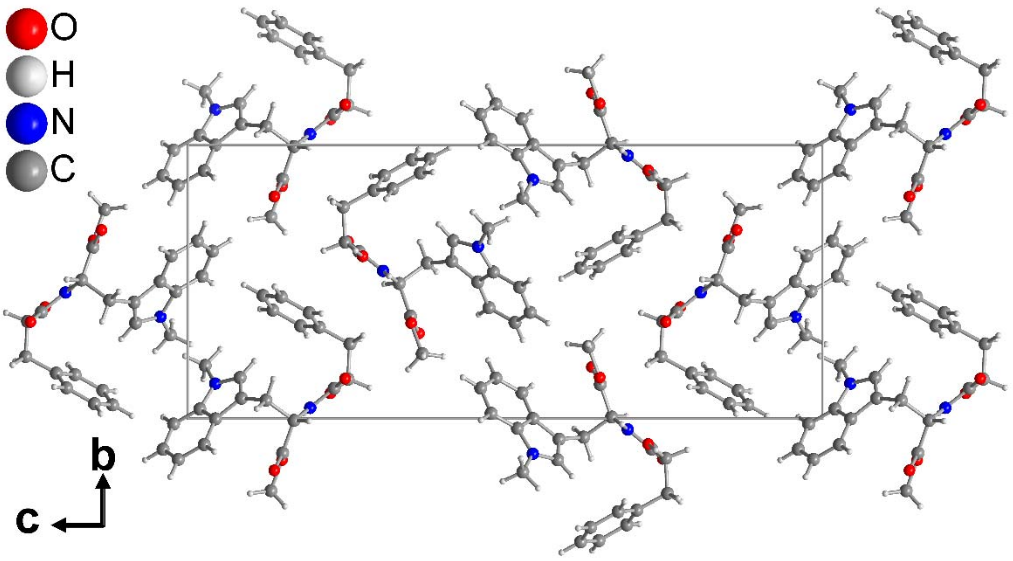

Figure S69. Section of the packing diagram of **1** along the *a*-plane.

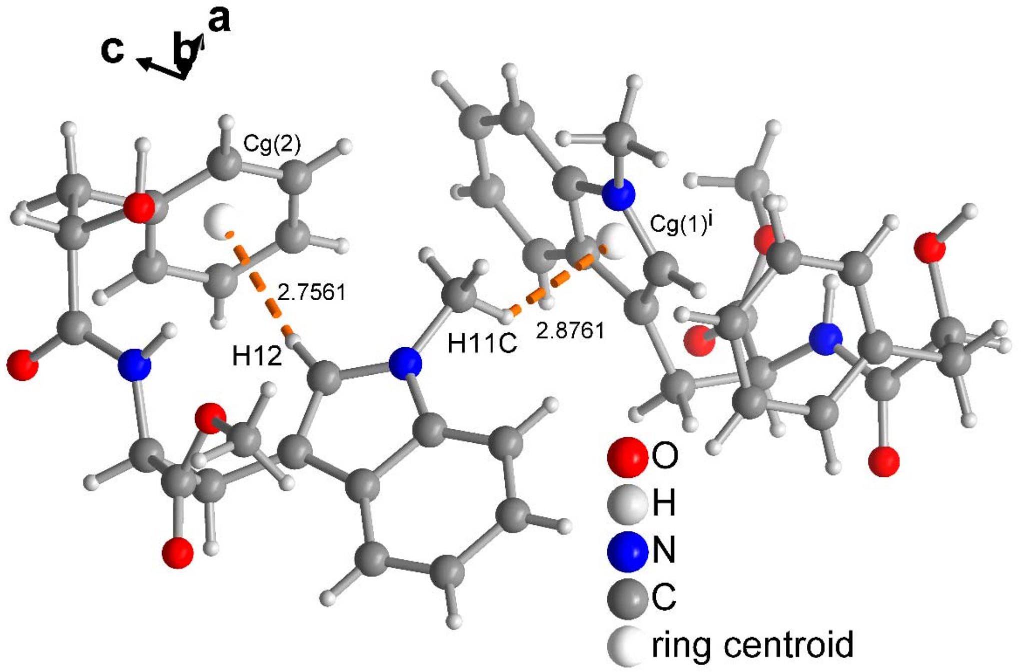

Figure S70. Section of the packing diagram of **1** showing short C-H···π distances (orange dashed lines), Symmetry code: (i) 1/2+x, 3/2–y, 1–z.

Fractional atomic coordinates and isotropic or equivalent isotropic displacement parameters (Å^2) for **1**

	x	*y*	*z*	U_{iso}*/U_{eq}
O1	0.7399 (4)	0.34906 (17)	0.64836 (7)	0.0909 (11)
O2	0.3774 (4)	0.31137 (19)	0.63605 (7)	0.0945 (11)
O3	0.4813 (4)	0.64653 (17)	0.74768 (8)	0.0954 (11)
O4	1.0531 (4)	0.59272 (17)	0.72463 (7)	0.0919 (11)
H4	1.206 (7)	0.614 (3)	0.7363 (12)	0.138*
N1	0.8953 (4)	0.6281 (2)	0.54317 (10)	0.0904 (11)
N2	0.6705 (4)	0.5395 (2)	0.69261 (10)	0.0829 (12)
C1	0.5235 (7)	0.3746 (3)	0.64940 (10)	0.0836 (12)
C1'	0.6583 (6)	0.6107 (3)	0.73038 (12)	0.0816 (12)
C2	0.4785 (5)	0.4938 (2)	0.66719 (10)	0.0836 (12)
H2A	0.349909	0.491273	0.690481	0.100*
C2'	0.8812 (5)	0.6445 (2)	0.75231 (11)	0.0868 (12)
H2'	0.888295	0.613592	0.786246	0.104*
C3	0.4106 (5)	0.5676 (2)	0.62314 (10)	0.0872 (12)
H3A	0.272345	0.536166	0.608656	0.105*
H3B	0.376498	0.644986	0.634843	0.105*
C3'	0.9070 (6)	0.7730 (2)	0.75521 (11)	0.0902 (12)
H3'A	0.785967	0.803460	0.776048	0.108*
H3'B	1.050747	0.790365	0.771485	0.108*
C4	0.5855 (5)	0.5748 (3)	0.58442 (11)	0.0835 (12)
C4'	0.9013 (6)	0.8338 (2)	0.70702 (11)	0.0875 (12)
C5	0.6170 (6)	0.4994 (3)	0.54359 (11)	0.0840 (12)
C5'	0.7087 (6)	0.8873 (3)	0.69095 (15)	0.0965 (13)
H5'	0.577497	0.884189	0.710434	0.116*
C6	0.4996 (6)	0.4054 (3)	0.52583 (12)	0.0917 (12)
H6	0.367778	0.379614	0.541631	0.110*
C6'	0.7037 (7)	0.9451 (3)	0.64715 (17)	0.1066 (14)
H6'	0.570499	0.981659	0.636781	0.128*
C7	0.5797 (7)	0.3513 (3)	0.48497 (13)	0.0979 (13)
H7	0.501918	0.287306	0.472672	0.118*

C7'	0.8941 (9)	0.9495 (3)	0.61842 (13)	0.1069 (13)
H7'	0.891743	0.988855	0.588197	0.128*
C8	0.7730 (7)	0.3883 (3)	0.46108 (12)	0.1003 (13)
H8	0.823619	0.348991	0.432935	0.120*
C8'	1.0863 (7)	0.8967 (3)	0.63383 (14)	0.0998 (13)
H8'	1.217081	0.899732	0.614213	0.120*
C9	0.8907 (6)	0.4804 (3)	0.47759 (13)	0.0952 (12)
H9	1.021989	0.505636	0.461436	0.114*
C9'	1.0901 (6)	0.8393 (3)	0.67758 (14)	0.0928 (12)
H9'	1.223776	0.802880	0.687774	0.111*
C10	0.8101 (6)	0.5353 (3)	0.51892 (12)	0.0859 (12)
C11	1.0860 (5)	0.6943 (3)	0.52737 (12)	0.1017 (13)
H11A	1.217021	0.645014	0.524475	0.153*
H11B	1.116301	0.753975	0.551336	0.153*
H11C	1.053512	0.728786	0.495626	0.153*
C12	0.7584 (6)	0.6502 (3)	0.58223 (12)	0.0876 (12)
H12	0.781007	0.710081	0.604827	0.105*
C13	0.7951 (5)	0.2372 (3)	0.63082 (12)	0.1049 (13)
H13A	0.733066	0.226758	0.598035	0.157*
H13B	0.731790	0.180286	0.652883	0.157*
H13C	0.957931	0.228532	0.629644	0.157*
H2	0.811 (6)	0.520 (3)	0.6831 (12)	0.126*

Atomic displacement parameters ($Å^2$) for **1.**

	U^{11}	U^{22}	U^{33}	U^{12}	U^{13}	U^{23}
O1	0.0855 (19)	0.0889 (17)	0.0982 (17)	0.0025 (11)	-0.0020 (11)	-0.0061 (12)
O2	0.0917 (19)	0.0958 (17)	0.0960 (16)	-0.0110 (12)	-0.0008 (12)	-0.0061 (11)
O3	0.0770 (18)	0.1059 (17)	0.1031 (16)	-0.0009 (12)	0.0050 (11)	-0.0147 (12)
O4	0.0755 (16)	0.0964 (16)	0.1039 (16)	0.0030 (11)	-0.0009 (12)	-0.0090 (10)
N1	0.085 (2)	0.095 (2)	0.091 (2)	-0.0040 (18)	0.0018 (17)	0.0051 (16)
N2	0.074 (2)	0.0886 (18)	0.0865 (19)	-0.0033 (14)	-0.0033 (16)	-0.0085 (16)
C1	0.081 (3)	0.092 (3)	0.079 (2)	-0.005 (2)	0.0023 (17)	0.0034 (18)
C1'	0.078 (3)	0.083 (2)	0.085 (2)	-0.0017 (19)	0.002 (2)	0.0008 (19)

C2	0.081 (2)	0.087 (2)	0.083 (2)	-0.0026 (17)	0.0012 (17)	-0.0048 (18)
C2'	0.077 (3)	0.097 (2)	0.087 (2)	0.0001 (19)	0.0000 (19)	-0.0057 (17)
C3	0.084 (2)	0.088 (2)	0.090 (2)	-0.0010 (17)	0.0008 (19)	-0.0023 (17)
C3'	0.086 (2)	0.090 (2)	0.095 (2)	-0.0037 (17)	-0.0017 (18)	-0.0123 (18)
C4	0.081 (3)	0.084 (2)	0.086 (2)	0.000 (2)	-0.002 (2)	0.0040 (18)
C4'	0.087 (3)	0.080 (2)	0.095 (2)	-0.002 (2)	-0.003 (2)	-0.0084 (17)
C5	0.084 (3)	0.086 (2)	0.082 (2)	0.006 (2)	-0.003 (2)	0.004 (2)
C5'	0.088 (3)	0.087 (2)	0.115 (3)	0.000 (2)	-0.004 (2)	-0.009 (2)
C6	0.096 (3)	0.092 (2)	0.087 (2)	-0.002 (2)	-0.0053 (18)	-0.0007 (19)
C6'	0.100 (3)	0.089 (3)	0.131 (3)	-0.0008 (19)	-0.016 (3)	0.003 (2)
C7	0.114 (3)	0.093 (2)	0.087 (2)	0.001 (2)	-0.005 (2)	-0.002 (2)
C7'	0.115 (4)	0.092 (2)	0.114 (3)	-0.013 (2)	-0.013 (3)	0.0082 (19)
C8	0.111 (3)	0.104 (3)	0.086 (2)	0.016 (2)	0.005 (2)	0.002 (2)
C8'	0.100 (3)	0.097 (2)	0.103 (3)	-0.011 (2)	0.001 (2)	0.002 (2)
C9	0.091 (3)	0.103 (3)	0.092 (3)	0.006 (2)	0.002 (2)	0.006 (2)
C9'	0.087 (3)	0.092 (2)	0.100 (2)	-0.0044 (18)	-0.001 (2)	-0.007 (2)
C10	0.086 (3)	0.088 (3)	0.084 (2)	0.0054 (19)	0.000 (2)	0.003 (2)
C11	0.086 (3)	0.109 (3)	0.111 (2)	-0.011 (2)	0.0017 (18)	0.0147 (19)
C12	0.090 (3)	0.087 (2)	0.085 (2)	0.004 (2)	0.000 (2)	-0.0001 (18)
C13	0.108 (3)	0.089 (3)	0.117 (2)	0.016 (2)	-0.0051 (19)	-0.0199 (19)

Geometric parameters (Å, º) for **1**

O1—C1	1.330 (4)	C4'—C9'	1.390 (4)
O1—C13	1.444 (3)	C5—C6	1.402 (4)
O2—C1	1.207 (3)	C5—C10	1.404 (4)
O3—C1'	1.235 (4)	C5'—C6'	1.381 (4)
O4—C2'	1.417 (3)	C5'—H5'	0.9500
O4—H4	1.00 (4)	C6—C7	1.375 (4)
N1—C12	1.373 (4)	C6—H6	0.9500
N1—C10	1.380 (4)	C6'—C7'	1.386 (5)
N1—C11	1.449 (4)	C6'—H6'	0.9500
N2—C1'	1.336 (4)	C7—C8	1.398 (4)
N2—C2	1.448 (4)	C7—H7	0.9500

N2—H2	0.91 (4)	C7'—C8'	1.375 (5)
C1—C2	1.514 (4)	C7'—H7'	0.9500
C1'—C2'	1.516 (4)	C8—C9	1.372 (4)
C2—C3	1.543 (4)	C8—H8	0.9500
C2—H2A	1.0000	C8'—C9'	1.378 (4)
C2'—C3'	1.528 (4)	C8'—H8'	0.9500
C2'—H2'	1.0000	C9—C10	1.391 (4)
C3—C4	1.493 (4)	C9—H9	0.9500
C3—H3A	0.9900	C9'—H9'	0.9500
C3—H3B	0.9900	C11—H11A	0.9800
C3'—C4'	1.504 (4)	C11—H11B	0.9800
C3'—H3'A	0.9900	C11—H11C	0.9800
C3'—H3'B	0.9900	C12—H12	0.9500
C4—C12	1.366 (4)	C13—H13A	0.9800
C4—C5	1.442 (4)	C13—H13B	0.9800
C4'—C5'	1.387 (4)	C13—H13C	0.9800
C1—O1—C13	115.9 (2)	C10—C5—C4	107.2 (3)
C2'—O4—H4	112 (2)	C6'—C5'—C4'	121.3 (3)
C12—N1—C10	107.8 (3)	C6'—C5'—H5'	119.4
C12—N1—C11	126.9 (3)	C4'—C5'—H5'	119.4
C10—N1—C11	125.1 (3)	C7—C6—C5	118.4 (3)
C1'—N2—C2	124.3 (3)	C7—C6—H6	120.8
C1'—N2—H2	116 (2)	C5—C6—H6	120.8
C2—N2—H2	120 (2)	C5'—C6'—C7'	119.7 (3)
O2—C1—O1	123.9 (3)	C5'—C6'—H6'	120.2
O2—C1—C2	123.0 (3)	C7'—C6'—H6'	120.2
O1—C1—C2	113.1 (3)	C6—C7—C8	121.6 (3)
O3—C1'—N2	124.0 (3)	C6—C7—H7	119.2
O3—C1'—C2'	120.8 (3)	C8—C7—H7	119.2
N2—C1'—C2'	115.1 (3)	C8'—C7'—C6'	119.7 (3)
N2—C2—C1	111.1 (3)	C8'—C7'—H7'	120.1

N2—C2—C3	112.0 (2)	C6'—C7'—H7'	120.1
C1—C2—C3	108.6 (2)	C9—C8—C7	121.1 (3)
N2—C2—H2A	108.3	C9—C8—H8	119.4
C1—C2—H2A	108.3	C7—C8—H8	119.4
C3—C2—H2A	108.3	C7'—C8'—C9'	120.3 (3)
O4—C2'—C1'	108.2 (2)	C7'—C8'—H8'	119.9
O4—C2'—C3'	112.5 (3)	C9'—C8'—H8'	119.9
C1'—C2'—C3'	111.8 (3)	C8—C9—C10	117.4 (3)
O4—C2'—H2'	108.1	C8—C9—H9	121.3
C1'—C2'—H2'	108.1	C10—C9—H9	121.3
C3'—C2'—H2'	108.1	C8'—C9'—C4'	121.0 (3)
C4—C3—C2	113.9 (2)	C8'—C9'—H9'	119.5
C4—C3—H3A	108.8	C4'—C9'—H9'	119.5
C2—C3—H3A	108.8	N1—C10—C9	129.4 (4)
C4—C3—H3B	108.8	N1—C10—C5	108.1 (3)
C2—C3—H3B	108.8	C9—C10—C5	122.5 (3)
H3A—C3—H3B	107.7	N1—C11—H11A	109.5
C4'—C3'—C2'	115.2 (2)	N1—C11—H11B	109.5
C4'—C3'—H3'A	108.5	H11A—C11—H11B	109.5
C2'—C3'—H3'A	108.5	N1—C11—H11C	109.5
C4'—C3'—H3'B	108.5	H11A—C11—H11C	109.5
C2'—C3'—H3'B	108.5	H11B—C11—H11C	109.5
H3'A—C3'—H3'B	107.5	C4—C12—N1	111.3 (3)
C12—C4—C5	105.6 (3)	C4—C12—H12	124.4
C12—C4—C3	126.8 (3)	N1—C12—H12	124.4
C5—C4—C3	127.4 (3)	O1—C13—H13A	109.5
C5'—C4'—C9'	118.0 (3)	O1—C13—H13B	109.5
C5'—C4'—C3'	121.0 (3)	H13A—C13—H13B	109.5
C9'—C4'—C3'	120.9 (3)	O1—C13—H13C	109.5
C6—C5—C10	118.9 (3)	H13A—C13—H13C	109.5
C6—C5—C4	133.9 (3)	H13B—C13—H13C	109.5

Hydrogen-bond geometry (Å, º) for **1**

D—H···A	D—H	H···A	D···A	D—H···A
O4—H4···O3^{i}	1.00 (4)	1.72 (4)	2.714 (3)	171 (3)
C2—H2A···O4^{ii}	1.00	2.34	3.212 (4)	146
C3′ —H3′ A···O2^{iii}	0.99	2.60	3.462 (4)	145
C3′ —H3′ B···O1^{iv}	0.99	2.62	3.500 (4)	148
N2—H2···O4	0.91 (4)	2.03 (3)	2.531 (3)	113 (3)

Symmetry codes: (i) x+1, y, z; (ii) x-1, y, z; (iii) -x+1, y+1/2, -z+3/2; (iv) -x+2, y+1/2, -z+3/2.

Results of X-ray analysis of compound 2

Crystal data

$C_{21}H_{22}N_2O_4·CH_4O$	$F(000) = 848$
$M_r = 398.45$	$D_x = 1.283$ Mg m^{-3}
Monoclinic, $C2$	Cu $K\alpha$ radiation, $\lambda = 1.54178$ Å
$a = 23.52$ (2) Å	Cell parameters from 9974 reflections
$b = 5.994$ (5) Å	$\theta = 5.9–67.5°$
$c = 15.843$ (13) Å	$\mu = 0.75$ mm^{-1}
$\beta = 112.574$ (17)°	$T = 140$ K
$V = 2063$ (3) Å^3	Needle, clear colourless
$Z = 4$	0.20 × 0.10 × 0.05 mm^3

Data collection

Bruker Kappa APEX-II CCD area detector diffractometer	3416 independent reflections
Radiation source: microfocus sealed tube	3283 reflections with $I > 2\sigma(I)$
Multilayer mirror monochromator	$R_{int} = 0.027$
ω scans, ϕ scans	$\theta_{max} = 65.6°$, $\theta_{min} = 5.9°$
Absorption correction: multi-scan (*SADABS*; Sheldrick, 1996)	$h = -27\rightarrow27$
$T_{min} = 0.917$, $T_{max} = 1.000$	$k = -7\rightarrow7$
11796 measured reflections	$l = -15\rightarrow18$

Refinement

Refinement on F^2	Secondary atom site location: difference Fourier map
Least-squares matrix: full	Hydrogen site location: mixed
$R[F^2 > 2\sigma(F^2)] = 0.025$	H atoms treated by a mixture of independent and constrained refinement
$wR(F^2) = 0.067$	$w = 1/[\sigma^2(F_o^2) + (0.0393P)^2 + 0.5585P]$ where $P = (F_o^2 + 2F_c^2)/3$
$S = 1.09$	$(\Delta/\sigma)_{max} = 0.001$
3416 reflections	$\Delta\rangle_{max}$ = 0.19 e Å^{-3}
276 parameters	$\Delta\rangle_{min}$ = -0.20 e Å^{-3}
1 restraint	Absolute structure: Flack x determined using 1433 quotients [(I+)-(I-)]/[(I+)+(I-)] (Parsons, Flack and Wagner, ActaCryst. B69 (2013) 249-259).
Primary atom site location: structure-invariant direct methods	Absolute structure parameter: 0.00 (3)

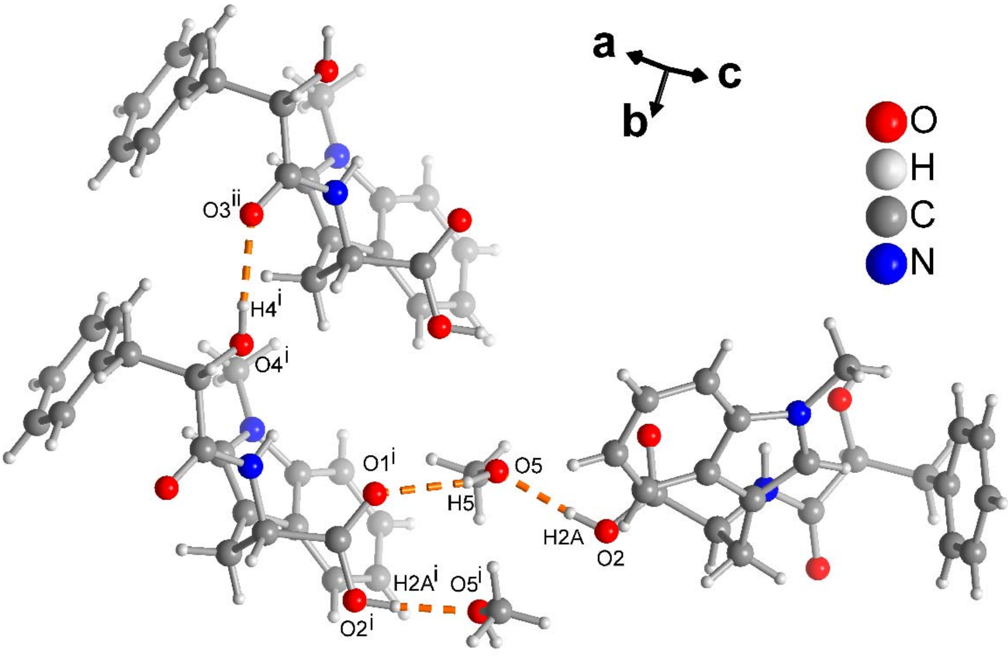

Figure S71. Section of the packing diagram of **2** showing the intermolecular H-bonds (orange dashed line) with methanole, which connect three symmetry equivalent molecules. Details for intermolecular H-bonds:

O2-H2A0.85 Å, H2A···O5 1.76 Å, O2···O52.61 Å, O4-H4···O5 172 °

O5-H5 0.81 Å, H5···O1^{i}2.03 Å, O5···O1^{i}2.80 Å, O5-H5···O1^{i} 158 °

O4^{i}-H4^{i} 0.84 Å, H4^{i}···O3^{ii}1.92 Å, O4^{i}···O3^{ii}2.74 Å, O4^{i}-H4^{i}···O3^{ii} 169 °

Symmetry codes: (i) 3/2–x, 1/2+y, 1–z, (ii) 3/2–x, –1/2+y, 1–z.

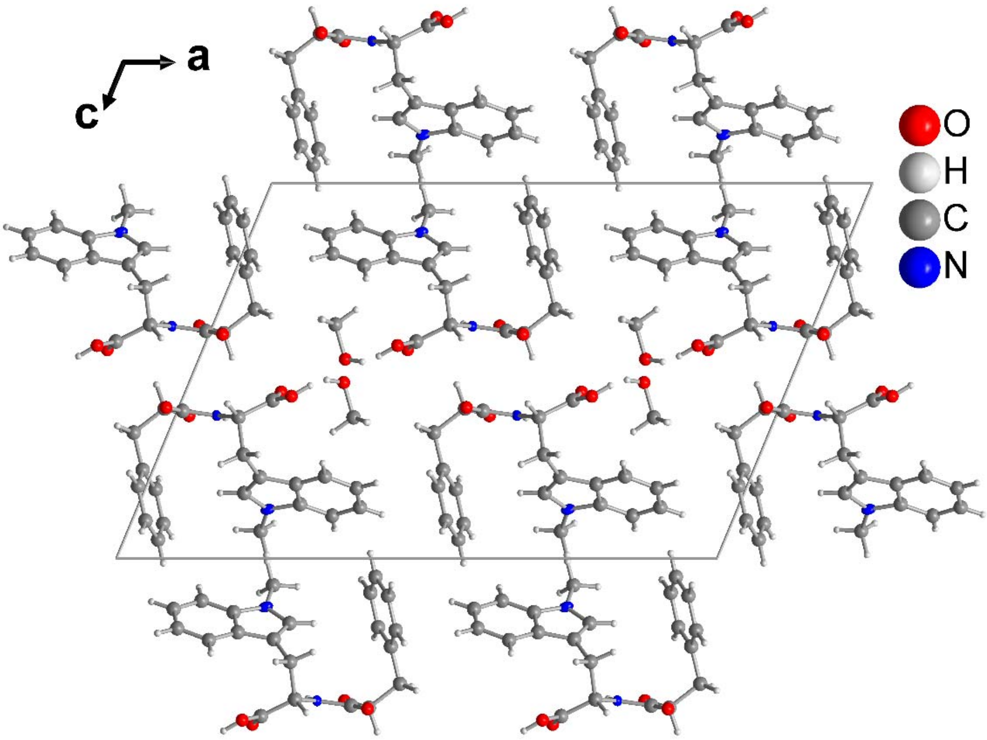

Figure S72. Sections of the packing diagram of **2** along the *b*-plane.

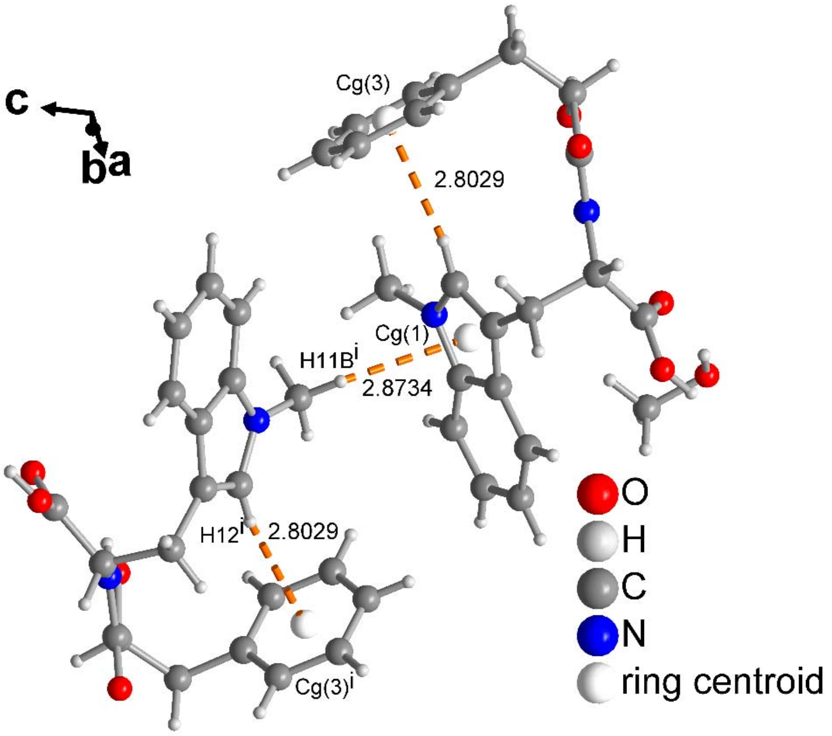

Figure S73. Section of the packing diagram of **2** showing short inter- and intramolecular C-H···π distances (orange dashed lines). Symmetry code: (i) 3/2–x, 1/2+y, 2–z.

Fractional atomic coordinates and isotropic or equivalent isotropic displacement parameters (Å^2) for **2**(Note that the atomic numbering in the deposited cif file is different to avoid the prime notation, that is C1' is C13, C2' is C14 etc.)

	x	*y*	*z*	U_{iso}*/U_{eq}
O1	0.65940 (5)	0.4911 (2)	0.55654 (9)	0.0217 (3)
H1	0.5844 (11)	0.409 (5)	0.6253 (16)	0.033*
C1'	0.50817 (8)	0.5468 (3)	0.60666 (12)	0.0152 (4)
C1	0.65075 (8)	0.6794 (3)	0.57838 (12)	0.0178 (4)
N1	0.71816 (7)	0.3323 (3)	0.86983 (11)	0.0250 (4)
C2'	0.47682 (7)	0.3204 (3)	0.60061 (12)	0.0169 (4)
H2'	0.446506	0.299653	0.536476	0.020*
H4	0.5045 (10)	0.027 (5)	0.6078 (15)	0.025*
C2	0.60559 (8)	0.7325 (3)	0.62335 (13)	0.0165 (4)
H2	0.577918	0.855216	0.587885	0.020*
O2	0.67925 (6)	0.8600 (2)	0.56768 (10)	0.0265 (3)
H2A	0.7028 (12)	0.838 (5)	0.5390 (19)	0.040*
N2	0.56793 (6)	0.5381 (2)	0.61929 (10)	0.0160 (3)
O3	0.47819 (5)	0.7223 (2)	0.59769 (9)	0.0200 (3)
C3'	0.44174 (7)	0.3112 (3)	0.66470 (13)	0.0199 (4)
H3'A	0.409841	0.429202	0.646610	0.024*
H3'B	0.420496	0.165526	0.656917	0.024*
C3	0.63959 (7)	0.8138 (3)	0.72350 (12)	0.0178 (4)
H3A	0.663461	0.949722	0.723412	0.021*
H3B	0.608736	0.854038	0.749306	0.021*
C4'	0.48241 (8)	0.3414 (3)	0.76430 (13)	0.0209 (4)
C4	0.68242 (8)	0.6407 (3)	0.78355 (13)	0.0168 (4)
O4	0.52225 (6)	0.1513 (2)	0.61967 (9)	0.0208 (3)
O5	0.74323 (6)	0.7709 (2)	0.46887 (10)	0.0268 (3)
H5	0.7733 (12)	0.847 (5)	0.4756 (18)	0.040*
C5'	0.48570 (9)	0.5465 (3)	0.80747 (15)	0.0278 (4)
H5'	0.462765	0.669628	0.773458	0.033*
C5	0.74738 (8)	0.6176 (3)	0.80238 (13)	0.0202 (4)
C6'	0.52212 (11)	0.5730 (4)	0.89961 (16)	0.0377 (5)

H6'	0.523658	0.713658	0.928073	0.045*
C6	0.79002 (9)	0.7470 (4)	0.78148 (15)	0.0297 (5)
H6	0.777465	0.879753	0.746484	0.036*
C7'	0.55615 (10)	0.3964 (4)	0.95023 (15)	0.0375 (5)
H7'	0.580928	0.415035	1.013260	0.045*
C7	0.85081 (10)	0.6769 (5)	0.81295 (16)	0.0432 (6)
H7	0.880124	0.763811	0.799645	0.052*
C9	0.82937 (9)	0.3510 (4)	0.88650 (15)	0.0359 (5)
H9	0.842420	0.218217	0.921334	0.043*
C9'	0.51729 (9)	0.1636 (3)	0.81604 (14)	0.0249 (4)
H9'	0.515962	0.022713	0.787889	0.030*
C8	0.86993 (9)	0.4796 (5)	0.86422 (17)	0.0443 (7)
H8	0.911732	0.434395	0.883824	0.053*
C8'	0.55377 (10)	0.1914 (4)	0.90802 (15)	0.0324 (5)
H8'	0.577237	0.069541	0.942298	0.039*
C13	0.70283 (11)	0.7748 (5)	0.37583 (17)	0.0506 (7)
H13A	0.726817	0.769836	0.337146	0.076*
H13B	0.678308	0.911986	0.363240	0.076*
H13C	0.675366	0.645225	0.362641	0.076*
C11	0.72095 (11)	0.1406 (3)	0.92782 (15)	0.0360 (5)
H11A	0.679408	0.104344	0.924008	0.054*
H11B	0.747256	0.175992	0.991249	0.054*
H11C	0.738067	0.012336	0.907200	0.054*
C12	0.66742 (8)	0.4651 (3)	0.82618 (13)	0.0209 (4)
H12	0.627514	0.438347	0.825792	0.025*
C10	0.76795 (8)	0.4230 (3)	0.85592 (13)	0.0239 (4)

Atomic displacement parameters (Å^2) for **2**(Note that the atomic numbering in the deposited cif file is different to avoid the prime notation, that is C1' is C13, C2' is C14 etc.)

	U^{11}	U^{22}	U^{33}	U^{12}	U^{13}	U^{23}
O1	0.0213 (6)	0.0195 (7)	0.0275 (8)	-0.0002 (5)	0.0130 (5)	-0.0035 (5)
C1'	0.0184 (8)	0.0161 (9)	0.0117 (9)	0.0006 (7)	0.0062 (6)	-0.0004 (6)

C1	0.0174 (8)	0.0212 (9)	0.0140 (10)	-0.0016 (7)	0.0048 (7)	0.0003 (7)
N1	0.0295 (8)	0.0200 (8)	0.0196 (9)	0.0003 (6)	0.0027 (6)	0.0021 (6)
C2'	0.0173 (8)	0.0133 (8)	0.0194 (9)	0.0009 (6)	0.0063 (7)	0.0012 (7)
C2	0.0191 (8)	0.0132 (8)	0.0185 (10)	0.0002 (6)	0.0086 (7)	0.0010 (6)
O2	0.0315 (7)	0.0230 (7)	0.0346 (9)	-0.0073 (6)	0.0236 (6)	-0.0033 (6)
N2	0.0166 (7)	0.0130 (7)	0.0193 (8)	0.0012 (6)	0.0080 (6)	0.0006 (6)
O3	0.0188 (6)	0.0148 (6)	0.0278 (8)	0.0018 (5)	0.0105 (5)	0.0015 (5)
C3'	0.0175 (8)	0.0174 (8)	0.0261 (10)	-0.0014 (7)	0.0099 (7)	0.0018 (7)
C3	0.0200 (8)	0.0145 (8)	0.0203 (10)	-0.0002 (7)	0.0092 (7)	-0.0020 (7)
C4'	0.0201 (8)	0.0232 (9)	0.0248 (11)	-0.0044 (7)	0.0145 (7)	0.0022 (8)
C4	0.0170 (8)	0.0164 (8)	0.0167 (9)	-0.0019 (6)	0.0064 (7)	-0.0039 (7)
O4	0.0210 (6)	0.0105 (6)	0.0321 (8)	0.0013 (5)	0.0115 (6)	0.0000 (5)
O5	0.0298 (7)	0.0242 (7)	0.0339 (9)	-0.0046 (5)	0.0205 (6)	0.0015 (6)
C5'	0.0362 (10)	0.0241 (10)	0.0278 (12)	-0.0037 (8)	0.0174 (8)	0.0014 (8)
C5	0.0196 (9)	0.0251 (9)	0.0153 (10)	-0.0022 (7)	0.0061 (7)	-0.0070 (7)
C6'	0.0554 (14)	0.0295 (11)	0.0322 (14)	-0.0120 (10)	0.0213 (11)	-0.0052 (9)
C6	0.0243 (9)	0.0434 (12)	0.0234 (11)	-0.0091 (9)	0.0113 (8)	-0.0080 (9)
C7'	0.0433 (12)	0.0448 (13)	0.0222 (12)	-0.0189 (10)	0.0104 (9)	-0.0002 (10)
C7	0.0221 (10)	0.0788 (18)	0.0320 (13)	-0.0113 (11)	0.0139 (9)	-0.0155 (12)
C9	0.0299 (10)	0.0456 (13)	0.0239 (11)	0.0139 (10)	0.0010 (8)	-0.0102 (10)
C9'	0.0248 (9)	0.0238 (10)	0.0290 (12)	-0.0009 (7)	0.0136 (8)	0.0040 (8)
C8	0.0177 (9)	0.0797 (19)	0.0316 (13)	0.0090 (10)	0.0052 (8)	-0.0190 (13)
C8'	0.0305 (10)	0.0365 (11)	0.0280 (12)	-0.0044 (9)	0.0088 (9)	0.0120 (9)
C13	0.0437 (13)	0.0762 (19)	0.0324 (14)	-0.0214 (13)	0.0149 (11)	-0.0051 (13)
C11	0.0502 (13)	0.0218 (10)	0.0263 (12)	-0.0008 (9)	0.0039 (10)	0.0046 (9)
C12	0.0194 (8)	0.0208 (9)	0.0204 (10)	-0.0023 (7)	0.0053 (7)	-0.0007 (7)
C10	0.0239 (9)	0.0269 (10)	0.0170 (10)	0.0044 (7)	0.0035 (7)	-0.0071 (7)

Geometric parameters (Å, º) for **2**(Note that the atomic numbering in the deposited cif file is different to avoid the prime notation, that is C1' is C13, C2' is C14 etc.)

O1—C1	1.220 (2)	O5—C13	1.415 (3)
C1'—O3	1.244 (2)	O5—H5	0.81 (3)
C1'—N2	1.343 (3)	C5'—C6'	1.389 (3)

C1'—C2'	1.529 (3)	C5'—H5'	0.9500
C1—O2	1.318 (2)	C5—C6	1.404 (3)
C1—C2	1.523 (3)	C5—C10	1.414 (3)
N1—C12	1.379 (3)	C6'—C7'	1.383 (3)
N1—C10	1.383 (3)	C6'—H6'	0.9500
N1—C11	1.457 (3)	C6—C7	1.387 (3)
C2'—O4	1.419 (2)	C6—H6	0.9500
C2'—C3'	1.536 (3)	C7'—C8'	1.390 (4)
C2'—H2'	1.0000	C7'—H7'	0.9500
C2—N2	1.450 (2)	C7—C8	1.407 (4)
C2—C3	1.556 (3)	C7—H7	0.9500
C2—H2	1.0000	C9—C8	1.374 (4)
O2—H2A	0.85 (3)	C9—C10	1.404 (3)
N2—H1	0.85 (3)	C9—H9	0.9500
C3'—C4'	1.510 (3)	C9'—C8'	1.388 (3)
C3'—H3'A	0.9900	C9'—H9'	0.9500
C3'—H3'B	0.9900	C8—H8	0.9500
C3—C4	1.503 (2)	C8'—H8'	0.9500
C3—H3A	0.9900	C13—H13A	0.9800
C3—H3B	0.9900	C13—H13B	0.9800
C4'—C5'	1.395 (3)	C13—H13C	0.9800
C4'—C9'	1.401 (3)	C11—H11A	0.9800
C4—C12	1.368 (3)	C11—H11B	0.9800
C4—C5	1.447 (3)	C11—H11C	0.9800
O4—H4	0.84 (3)	C12—H12	0.9500
O3—C1'—N2	124.38 (16)	C4'—C5'—H5'	119.6
O3—C1'—C2'	120.32 (15)	C6—C5—C10	119.08 (18)
N2—C1'—C2'	115.28 (15)	C6—C5—C4	133.80 (18)
O1—C1—O2	125.08 (17)	C10—C5—C4	107.07 (17)
O1—C1—C2	123.25 (16)	C7'—C6'—C5'	120.5 (2)
O2—C1—C2	111.66 (15)	C7'—C6'—H6'	119.7

C12—N1—C10	108.42 (17)	C5'—C6'—H6'	119.7
C12—N1—C11	126.31 (18)	C7—C6—C5	118.7 (2)
C10—N1—C11	125.05 (18)	C7—C6—H6	120.7
O4—C2'—C1'	108.35 (14)	C5—C6—H6	120.7
O4—C2'—C3'	112.68 (14)	C6'—C7'—C8'	119.4 (2)
C1'—C2'—C3'	111.02 (15)	C6'—C7'—H7'	120.3
O4—C2'—H2'	108.2	C8'—C7'—H7'	120.3
C1'—C2'—H2'	108.2	C6—C7—C8	121.3 (2)
C3'—C2'—H2'	108.2	C6—C7—H7	119.4
N2—C2—C1	109.60 (15)	C8—C7—H7	119.4
N2—C2—C3	111.28 (15)	C8—C9—C10	117.6 (2)
C1—C2—C3	111.40 (15)	C8—C9—H9	121.2
N2—C2—H2	108.1	C10—C9—H9	121.2
C1—C2—H2	108.1	C8'—C9'—C4'	120.7 (2)
C3—C2—H2	108.1	C8'—C9'—H9'	119.7
C1—O2—H2A	114 (2)	C4'—C9'—H9'	119.7
C1'—N2—C2	124.22 (15)	C9—C8—C7	121.4 (2)
C1'—N2—H1	117.1 (16)	C9—C8—H8	119.3
C2—N2—H1	118.7 (16)	C7—C8—H8	119.3
C4'—C3'—C2'	113.64 (15)	C9'—C8'—C7'	120.4 (2)
C4'—C3'—H3'A	108.8	C9'—C8'—H8'	119.8
C2'—C3'—H3'A	108.8	C7'—C8'—H8'	119.8
C4'—C3'—H3'B	108.8	O5—C13—H13A	109.5
C2'—C3'—H3'B	108.8	O5—C13—H13B	109.5
H3'A—C3'—H3'B	107.7	H13A—C13—H13B	109.5
C4—C3—C2	112.74 (15)	O5—C13—H13C	109.5
C4—C3—H3A	109.0	H13A—C13—H13C	109.5
C2—C3—H3A	109.0	H13B—C13—H13C	109.5
C4—C3—H3B	109.0	N1—C11—H11A	109.5
C2—C3—H3B	109.0	N1—C11—H11B	109.5
H3A—C3—H3B	107.8	H11A—C11—H11B	109.5
C5'—C4'—C9'	118.27 (19)	N1—C11—H11C	109.5

C5'—C4'—C3'	120.78 (17)	H11A—C11—H11C	109.5
C9'—C4'—C3'	120.94 (18)	H11B—C11—H11C	109.5
C12—C4—C5	106.08 (16)	C4—C12—N1	110.73 (17)
C12—C4—C3	127.31 (16)	C4—C12—H12	124.6
C5—C4—C3	126.48 (17)	N1—C12—H12	124.6
C2'—O4—H4	108.4 (16)	N1—C10—C9	130.3 (2)
C13—O5—H5	109.2 (19)	N1—C10—C5	107.70 (16)
C6'—C5'—C4'	120.79 (19)	C9—C10—C5	122.0 (2)
C6'—C5'—H5'	119.6		

Hydrogen-bond geometry (Å, º) for **2**

D—H···*A*	*D*—H	H···*A*	*D*···*A*	*D*—H···*A*
O2—H2···O5	0.85 (3)	1.76 (3)	2.609 (2)	172 (3)
O4—H4···O3^{i}	0.85 (3)	1.91 (3)	2.744 (3)	169 (2)
N2—H2*A*···O4	0.84 (3)	2.11 (2)	2.557 (3)	112.6 (18)
C2—H2B···O4^{ii}	1.00	2.37	3.172 (3)	136
C2′—H2′···O1^{iii}	1.00	2.62	3.375 (3)	132
O5—H5···O1^{iv}	0.81 (3)	2.03 (3)	2.803 (3)	158 (3)

Symmetry codes: (i) *x*, *y*-1, *z*; (ii) *x*, *y*+1, *z*; (iii) -*x*+1, *y*, -*z*+1; (iv) -*x*+3/2, *y*+1/2, -*z*+1.
